# A Tri-Direction Guided Moss Growth Optimizer with Adaptive Differential Evolution for Engineering Design Problems

**DOI:** 10.3390/biomimetics11090660

**Published:** 2026-09-14

**Authors:** Changlong Pang, Yukun Wang, Wansheng Cheng

**Affiliations:** School of Electronic and Information Engineering, University of Science and Technology Liaoning, Anshan 114051, China; pcl@stu.ustl.edu.cn (C.P.); cws@ustl.edu.cn (W.C.)

**Keywords:** moss growth optimization, three-directional guidance, differential evolution, adaptive opposition-based learning

## Abstract

This article proposes an improved Moss Growth Optimization (IMGO) algorithm to address the drawbacks of imbalanced exploration and exploitation and susceptibility to local optima in the original MGO. IMGO integrates three-dimensional guidance, elite guidance, adaptive search, and adaptive adversarial learning to achieve a dynamic balance between global exploration and local development. IMGO is compared with eight metaheuristic algorithms on CEC2017 and CEC2022 benchmarks. Results show IMGO ranks first in 30D and 50D CEC2017 with scores of 49 and 64, surpassing second-ranked CFDA (87 and 81). For 20D CEC2022, IMGO takes first place with a score of 27, while original MGO scores 66 and ranks seventh. The Friedman test validates its strong robustness with a statistic of 1.6897, lower than CFDA’s 3.0000. Furthermore, IMGO acquires the optimal average solutions for all six engineering design problems. Experiments verify that IMGO has comprehensive advantages in accuracy and robustness, providing a reliable method for complex optimization problems.

## 1. Introduction

As a core approach to improving production efficiency and reducing resource consumption, optimization technology has been widely applied in scientific research and engineering practice [1]. With the continuous development of engineering technology, optimization problems in typical scenarios such as path planning [2], resource scheduling [3], medical applications [4] and image processing [5] have become more complex, generally exhibiting prominent characteristics such as high dimensionality, nonlinearity, and multiple constraints. From the perspective of mathematical classification, optimization problems can typically be categorized into two types: unconstrained optimization and constrained optimization. Traditional gradient-based optimization algorithms have obvious limitations in addressing such complex problems. They not only impose stringent requirements on the properties of the objective function and are prone to falling into local optimal solutions, but also suffer from slow convergence speed and strong parameter sensitivity. Furthermore, they are susceptible to the “combinatorial explosion” phenomenon in high-dimensional spaces, leading to a sharp increase in computational complexity [6], which makes it difficult to stably obtain high-quality optimization results in practical applications.

Faced with the application bottlenecks of traditional optimization algorithms, metaheuristic algorithms have emerged and attracted widespread attention. Unlike traditional methods, these algorithms do not rely on gradient information; instead, their search strategies are inspired by natural biological behaviors or physical laws, which endows them with superior global search capabilities and robustness. Their core advantage lies in the effective handling of complex optimization problems characterized by non-convexity, non-differentiability, or high noise levels, and they have currently become key technical tools for solving challenging optimization tasks in real-world scenarios. Classic metaheuristic algorithms such as Particle Swarm Optimization (PSO) [7], Genetic Algorithm (GA) [8], and Differential Evolution (DE) [9] have long attracted extensive attention from both academic and engineering communities due to their robust performance across various optimization scenarios, spurring the development of numerous improved algorithms. However, as the complexity of practical optimization problems continues to escalate, these traditional metaheuristic algorithms have gradually revealed their inherent limitations. On the one hand, they are prone to premature convergence, making it difficult to escape local optimal solutions; on the other hand, in the later stages of the iterative process, the convergence rate tends to exhibit a significant declining trend. To break through the aforementioned technical bottlenecks, relevant researchers are focusing on developing advanced metaheuristic algorithms with higher efficiency and stronger environmental adaptability.

Inspired by the growth pattern of moss in nature, an efficient swarm intelligence algorithm named the Moss Growth Optimization (MGO) algorithm is proposed. This algorithm classifies the population based on the current optimal individual and calculates the difference between dominant individuals and the optimal individual to determine the evolutionary direction of the population. MGO achieves the optimal solution through three core strategies: spore diffusion search, dual reproductive search, and cryptobiosis mechanism. With favorable performance, the MGO Algorithm has been successfully applied to engineering design problems. In recent years, many improved optimization algorithms have been proposed to solve different problems. Wang et al. [10] proposed an enhanced Snake Optimizer (EMSO) and applied it to the quantitative structure–activity relationship (QSAR) modeling for environmental toxicity prediction. This algorithm can better configure hyperparameters and select fewer yet more effective features for environmental toxicity prediction models. Chen et al. [11] proposed a multi-strategy enhanced Nutcracker Optimization Algorithm (EMNOA) and applied it to the parameter extraction of solar photovoltaic (PV) cell models, thereby significantly reducing the parameter extraction errors of different PV models and providing a more robust and effective tool for solving this problem. Yang et al. [12] proposed a multi-strategy improved Whale Optimization Algorithm (MSWOA) and applied it to optimize the parameter selection of the Semi-Supervised Extreme Learning Machine (SSELM), which was ultimately used for logging lithology identification. It achieved high accuracy in logging identification applications and was significantly superior to other comparative models. He et al. [13] proposed an improved Butterfly Optimization Algorithm (FDCBOA) and applied it to the Economic Emission Dispatch (EED) problem considering various complex constraints. It significantly enhances the ability to handle complex constraints and escape local optima, demonstrating strong competitiveness across multiple evaluation metrics. Shan et al. [14] proposed an improved Heap-Based Optimizer (MGOHBO) and applied it to the field of medical diagnosis to optimize the Kernel Extreme Learning Machine (KELM) model. The proposed MGOHBO-KELM model achieves better diagnostic performance than other advanced models, demonstrating its effectiveness and practical value in solving real-world medical diagnosis problems. In-depth exploration of this research direction not only provides more efficient and feasible solution pathways for complex engineering optimization problems, but also plays a positive role in further enriching and advancing the optimization theory system.

Inspired by these enhanced algorithms, to improve the performance of the MGO algorithm and expand its applicability to engineering design problems, a series of improved MGO variants have been developed sequentially. Tong et al. [15] improved the original algorithm by integrating the crisscross strategy and dynamic grouping parameters, and successfully applied it to the production optimization problem of three-channel oil reservoirs, achieving a higher Net Present Value (NPV). Hou et al. [16] innovatively incorporated the Alpha evolutionary strategy and an adaptive β parameter to optimize the improved algorithm, then combined this algorithm with Support Vector Machine (SVM) and applied it to Spontaneous Intracerebral Hemorrhage (SICH) prediction. This provides strong support for the prognosis prediction of SICH patients. According to the No Free Lunch (NFL) theorem [17], there is no metaheuristic algorithm universally applicable to solving all optimization problems. Similarly, the implementation mechanisms and variants of MGO still have certain limitations, which can be summarized as follows:

**1.** 
When simulating “turbulent wind”, the algorithm adopts an excessively single diffusion direction with excessive randomness, failing to accurately reflect the multidirectionality and complex dynamic characteristics of real turbulent motion. This flaw results in low global exploration efficiency of the algorithm, making it prone to trapping in local optima during the search process and difficult to escape from them.
**2.** 
Throughout the entire iterative process, the algorithm adopts a fixed and single search behavior pattern, lacking the ability to dynamically adjust the intensities of “exploration” and “exploitation” according to the search progress. This rigid mechanism undermines the effective trade-off between “exploration” and “exploitation”, thereby impairing the overall optimization efficiency of the algorithm.
**3.** 
When spores or moss populations diffuse into non-optimal regions, their motion state tends to fall into stagnation, leading to a rapid loss of spatial distribution diversity of the populations. This issue not only weakens the global exploration potential of the algorithm but also easily triggers the phenomenon of premature convergence, ultimately reducing the global optimality of the obtained solutions.
**4.** 
Existing MGO variants only focus on single-dimensional optimization (such as production optimization and prediction model parameter tuning), fail to systematically solve the “exploration–exploitation” imbalance problem, and do not involve the collaborative design of multi-directional search and adaptive opposition-based learning. Most improved MGO algorithms rely on fixed search modes and lack phased dynamic adjustment mechanisms, resulting in difficulty in balancing convergence speed and solution accuracy in high-dimensional problems.

The limitations faced by the MGO algorithm are prevalent among existing metaheuristic optimization algorithms. To address this prevalent challenge, researchers have dedicated themselves to integrating the strengths of different algorithms to achieve performance breakthroughs. By leveraging the inherent strengths and performance merits of the MGO algorithm, this study proposes a novel three-directional guided moss growth algorithm integrated with adaptive differential evolution (IMGO). This algorithm is applied to addressing practical engineering optimization problems. Four core strategies are adopted to enchance MGO, and the specific optimizations in IMGO are outlined as follows: a three-directional guidance mechanism is introduced, which transforms the original linear turbulent diffusion mode into multidimensional collaborative exploration, thereby expanding the search range and enhancing population diversity; an adaptive parameter is defined to handle the search in phases, realizing the dynamic allocation of search capability and the specialized division of tasks. By focusing on early-stage exploration and late-stage exploitation, the convergence efficiency of the algorithm is improved, and the convergence performance and robustness of the algorithm are significantly enhanced; an elite-guided differential evolution formula based on new dimensions is defined to perform fine-grained search in the second stage, improving the convergence accuracy; By introducing an adaptive opposition-based learning strategy, the algorithm’s ability to escape from local optima is greatly enhanced through systematically exploring the opposite regions of the current search space. These strategies effectively overcome the limitations of the MGO. The main contributions of this paper can be summarized as follows:

**1.** 
An improved moss growth optimization algorithm (IMGO) has been proposed to solve complex numerical optimization problems in real life.
**2.** 
The IMGO integrates improved methods including the three-directional guidance mechanism, elite-guided dimensional differential evolution mechanism, combined search mechanism, and adaptive opposition-based learning mechanism. These innovations collectively address the limitations existing in the MGO, particularly the imbalance between its exploration and exploitation tendencies, as well as the propensity to prematurely converge to local optima.
**3.** 
The IMGO was compared with eight well-known algorithms on the CEC2017 and CEC2022 test suites. The results demonstrate that IMGO performs well in solving problems of different dimensions. Qualitative analysis, quantitative analysis, Friedman’s test, and Wilcoxon signed-rank test confirm the effectiveness of the strategy improvements from various perspectives.
**4.** 
In real-world engineering design problems, the IMGO also demonstrates better performance when compared with eight well-known algorithms and is significantly superior to the MGO. It provides new research ideas and technical approaches for solving real-world complex optimization problems.
**5.** 
This paper applies the MGO and the IMGO to photovoltaic parameter extraction for the first time.

Finally, the rest of this paper is organized as follows: Section 2 introduces the original MGO algorithm. Section 3 elaborates on the proposed IMGO algorithm in detail. Section 4 presents the experimental test results and statistical data analysis. Section 5 applies IMGO to solve eight engineering application problems. Section 6 provides the conclusions and future work.

## 2. An Overview of the MGO Algorithm

The MGO algorithm simulates the growth patterns of moss and incorporates its unique biological mechanisms, including asexual reproduction, sexual reproduction, vegetative propagation, and cryptobiosis. Through the organic integration of wind direction determination, spore diffusion search, dual propagation search, and the cryptobiosis mechanism, the algorithm finally achieves efficient global optimization and precise search capabilities.

### 2.1. Determine of Wind Direction

In the MGO algorithm, wind direction determination is a core mechanism responsible for identifying and guiding the evolutionary direction of the population. Its implementation process is as follows: the algorithm first classifies individuals based on their positional relationship with the current optimal individual. During classification, the algorithm compares the value of each dimension of the remaining individuals with that of the optimal individual, divides the population into two subsets accordingly, and selects and retains the larger subset. After repeating this division process d times, the resulting subset is defined as divX. Ultimately, the value of Wd is determined by calculating the average distance between all individuals in this relatively large multi-subgroup and the current optimal individual, a value denoted as the “wind direction”. This wind direction vector guides the population to evolve toward the global optimal solution, thereby effectively enchancing the algorithm’s ability to escape local optima. The specific calculation formula for Wd is shown in Equation (1).

(1) $$\begin{array}{c} Wd=\frac{1}{n}\sum_{i=1}^{n}X_{i},X_{i}\in dirX \end{array}$$

where dirX is the set of distances between the individuals in the selected subset and the optimal individual, and *n* is the size of the dirX set. The calculation of dirX is shown in Equation (2).

(2) $$\begin{array}{c} dirX=\left\{X_{BT}-X_{i}|X_{i}\in divX\right\} \end{array}$$

where $X_{BT}$ represents the best moss of the current iteration, and divX represents the individual subset obtained after multiple rounds of division.

### 2.2. Spore Dispersal Search (SDS)

The spore dispersal search mechanism effectively enhances the algorithm’s exploration capability in the global scope by simulating the natural phenomenon of moss spores spreading with the wind. The exploration formula is shown in Equation (3).

(3) $$\begin{array}{c} X_{i}^{new}=\left\{\begin{array}{cc} X_{i}+s1\cdot Wd, & r_{1}>d\\ X_{i}+s2\cdot Wd, & r_{1}\leq d \end{array}\right. \end{array}$$

where $X_{i}^{new}$ represents a novel moss by this mechanism, denotes the ith current moss in the population, $r_{1}$ is a random number uniformly distributed within the interval (0, 1), while *d* is a constant parameter that is set to 0.2 in this paper. s1 denotes the spore search step size under stable wind conditions, and s2 denotes the spore search step size under turbulent wind conditions. The calculations of these two step sizes are shown in Equation (4).

(4) $$\begin{array}{c} \left(s1,s2\right)=\left\{\begin{array}{c} s1=w\cdot (r_{2}-0.5)\cdot E\\ s2=0.1\cdot w\cdot \left(r_{3}-0.5\right)\cdot E\cdot \left[1+\frac{E}{2}\cdot \left(1+\tanh\beta \sqrt{1-\beta^{2}}\right)\right] \end{array}\right. \end{array}$$

where *w* is a constant parameter that is set to 2 in this paper, $r_{2}$ and $r_{3}$ are random numbers in the interval (0, 1). In addition, β represents the proportion of the population in divX to the population in *X*. The calculation of *E* is shown in Equation (5).

(5) $$\begin{array}{c} E=1-\frac{Fes}{MaxFes} \end{array}$$

where Fes denotes the present count of function evaluations, and MaxFes signifies the maximum number of iterations.

### 2.3. Dual Propagation Search (DPS)

The Dual Propagation Search is a mechanism designed to improve local exploitation efficiency. This mechanism updates individual positions through two approaches: first, it enables individuals to directly exchange values with the current optimal individual in the corresponding dimension; second, it uses the calculated Wd to correct specific dimensions of the individual. The local exploitation formula is shown in Equation (6).

(6) $$\left\{\begin{array}{cc} X_{i}^{\mathrm{new}}=\left(1-act\right)\cdot X_{i}+act\cdot X_{BT}, & r_{4}>d_{2}\\ X_{i,j}^{\mathrm{new}}=X_{BT,j}+s3\cdot W_{dj}, & r_{4}\leq d_{2} \end{array}\right.$$

where $X_{i}^{new}$ denotes the *i*-th new individual, and $X_{i,j}^{new}$ represents the *j*-th dimension of $X_{i}^{new}$, where *j* is a random number not exceeding the maximum dimension of the individual. $X_{BT,j}$ represents the *j*-th dimension of the optimal individual in the current iteration, and $Wd_{j}$ denotes the *j*-th dimension of Wd. $r_{4}$ is a random number that follows a uniform distribution within the range of (0, 1), and d2 is a constant parameter with a fixed value of 0.5. The calculation of act is shown in Equation (7), and the calculation of s3 is shown in Equation (8).

(7) $$\begin{array}{c} act=\left\{\begin{array}{cc} 1, & \frac{1}{1.5-10\cdot r_{5}}>0.5\\ 0, & \frac{1}{1.5-10\cdot r_{5}}\leq 0.5 \end{array}\right. \end{array}$$

where $r_{5}$ is a random number within the range (0, 1) and has the same dimension as $X_{BT}$.

(8) $$\begin{array}{c} s3=0.1\cdot (r_{6}-0.5)\cdot E \end{array}$$

where $r_{6}$ is a random number within the range (0, 1).

### 2.4. Cryptobiosis Mechanism

The cryptobiosis mechanism records the information of all individuals throughout the iterative process. When specific conditions are triggered, this mechanism is activated and employs the archived historically optimal individual to replace the current individual, thereby preserving the overall quality of the population. According to the original research, population updates are performed once every ten iterations. By enabling multiple explorations of the same position, this strategy not only maintains the high-quality characteristics of the population but also significantly enhances the algorithm’s global search capability. To gain a deeper understanding of the above process, readers are advised to refer to Reference [18] for more detailed information.

## 3. The Proposed IMGO Algorithm

To address the limitations of the standard MGO algorithm, the IMGO algorithm incorporates four improved strategies: 1. The idea of three-directional guidance draws inspiration from the Alpha-evolution operator in Ref. [19], while we reconstruct the sampling logic and embed it into the spore-diffusion framework of original MGO, which is a heavily adapted mechanism rather than direct adoption. 2. Elite-guided dimensional differential evolution is inspired by classical differential evolution, yet it takes the global elite individual as the mutation baseline and executes dimension-wise mutation tailored for MGO, which is our original design. 3. The phased-switch paradigm of adaptive search exists in existing metaheuristics; nevertheless, the nonlinear decreasing probability Pa constructed via the erf function is newly proposed in this paper for balancing exploration-exploitation of MGO. 4. Adaptive opposition-based learning originates from conventional opposition-based learning, but the quadratic-decay trigger probability Pb and the dual-random-vector update formula are our novel modifications. For details, please refer to the following sections.

### 3.1. Three-Directional Guidance

In the Spore dispersal search, the dispersal process is achieved solely by adjusting the corresponding step size, resulting in a unidirectional search direction that limits population diversity and compromises the algorithm’s global exploration capability. To mitigate this limitation, inspired by the Alpha operator [19], this study proposes a three-directional guidance strategy. First, this strategy randomly samples a new random matrix B from the original sample set X (refer to the selection of matrix *E* in Reference [19]) to introduce exploratory behavior and avoid local dependence. Meanwhile, it selects a high-quality sample matrix *R* from *X* to provide the globally optimal direction, as well as a suboptimal sample matrix W to supplement suboptimal regional information and prevent the optimal direction from falling into local optima. The update of the search direction is jointly guided by these three matrices, which not only ensures the correctness of the direction but also avoids path singularity. The pseudocode in Algorithm 1 illustrates how these three matrices are selected. The search formula of the strategy is shown in Equation (9).

(9) $$\begin{array}{c} X_{i}^{new}=\left\{\begin{array}{cc} X_{i}+t1\cdot Wd, & r>d_{1}\\ X_{i}+\theta \cdot \left(R_{i}-X_{i}+B_{i}-W_{i}\right), & r\leq d_{1} \end{array}\right. \end{array}$$

where θ is an uncertain control vector; its value can be a random number between 0 and 2, or a random vector with dimension nd and values ranging from 0 to 1. The random number mode enhances global exploration by expanding the step size, while the random vector mode enhances local exploitation through dimension adaptivity. These two modes switch randomly with equal probability, dynamically balancing exploration and exploitation. $B_{i}$, $R_{i}$, and $W_{i}$ denote the *i*-th individuals selected from the sampling matrix *B*, the high-quality matrix *R*, and the suboptimal matrix *W*, respectively, and satisfy the fitness value relationship: f(Ri)≤f(Bi)≤f(Wi).
**Algorithm 1:** Pseudo-code of IMGO
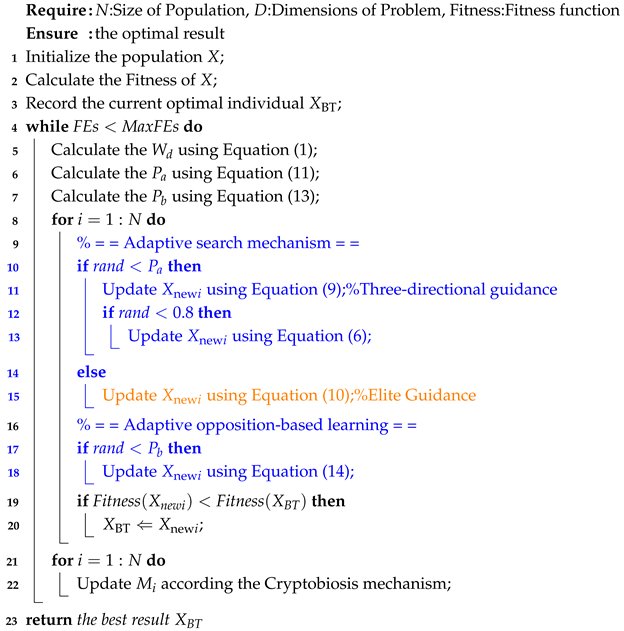


This strategy fuses the directions of three matrices: the high-quality matrix *R* (optimal direction), random matrix *B* (exploratory nature), and suboptimal matrix *W* (suboptimal regional information). Compared with the original algorithm, which relies only on the optimal individual for direction, it breaks the limitation of unidirectional diffusion in the original MGO, expands the search range, enhances population diversity, reduces the probability of converging to local optima, and lays a solid foundation for the global exploration capability of the IMGO algorithm.

### 3.2. Elite-Guided Dimensional Differential Evolution

While dual propagation search markedly enhances the algorithm’s convergence performance in the early iterative stage, it may severely restrict MGO’s ability to explore superior solutions in the later iterative stage. To mitigate this issue, this study formulates a novel update formula to achieve more refined local exploitation in the later iterative stage, the specific form of which is shown in Equation (10).

(10) $$\begin{array}{c} X_{i}^{new}=X_{BT}+r_{7}\cdot \bar{rand}\cdot \left(X_{r1}-X_{r2}\right) \end{array}$$

where $\bar{rand}$ denotes a random vector with the same dimension as the population, $r_{7}$ is a random number within the range (0, 1), and $X_{r1}$ and $X_{r2}$ represent distinct individuals in the population *X* that are different from the current individual $X_{i}$.

This strategy differs from the Differential Evolution (DE) algorithm. In the DE algorithm, two individuals are randomly selected to generate a mutation vector; this vector lacks directional guidance, making the DE algorithm prone to converging to local optima. In contrast, while maintaining the simplicity of the algorithm structure, this strategy takes the optimal individual $X_{BT}$ as the benchmark, uses a random vector (rand) to control the mutation amplitude, and performs differential mutation operations independently for each dimension. Using the optimal single $X_{BT}$ as the guide point accelerates convergence, and IMGO’s unique collaborative mechanism framework can avoid these issues. This strategy takes effect in the later stage, resulting in higher accuracy. This approach not only avoids convergence oscillations caused by excessive mutation but also prevents stagnation in local optima due to insufficient mutation, thereby improving the accuracy of local search.

### 3.3. Adaptive Search Mechanism

In the standard MGO algorithm, the SDS and DPS functions are continuous throughout the entire search process, which may lead to a slow convergence speed. In addition, their iteration strategy is relatively simple, limiting the algorithm’s ability to further explore better solutions. To address this problem, this paper proposes a phased combined search mechanism and introduces an adaptive parameter Pa (its calculation is shown in Equation (11) to achieve smooth switching between different search phases. The mechanism transition is shown in Equation (12).

(11) $$\begin{array}{c} Pa=\mathrm{erf}\left(\frac{-\alpha \cdot (\mathrm{MaxFes}-\mathrm{Fes})}{\mathrm{MaxFes}}\right) \end{array}$$

(12) $$\begin{array}{c} X_{i}^{new}=\left\{\begin{array}{cc} \mathrm{Equation}\;(9), & r<Pa\\ \mathrm{Equation}\;(10), & r\geq Pa \end{array}\right.\; \end{array}$$

where α is an important parameter that affects this mechanism, and its analysis and selection will be conducted in the experimental section. Fes denotes the current number of function evaluations, and MaxFes represents the maximum number of function evaluations.

Based on MaxFes and with Pa (a nonlinearly decreasing curve) as the transition condition, this mechanism focuses on exploration in the early stage and exploitation in the later stage, dynamically balancing the exploration–exploitation rhythm and improving the algorithm’s convergence efficiency.

### 3.4. Adaptive Opposition-Based Learning

In the original MGO algorithm, although the cryptobiosis mechanism is incorporated to facilitate escaping local optima, the algorithm may still stagnate when this mechanism is not activated. To address this issue, this study proposes an adaptive opposition-based learning (OBL) strategy. This strategy dynamically adjusts the trigger probability of opposition-based learning according to the iterative progress and enhances population diversity by exploring the opposition space of the current search region. By incorporating the adaptive parameter Pb, this strategy autonomously initiates OBL operations when specific conditions are satisfied. The calculation of parameter Pb is shown in Equation (13), and the update formula of opposition-based learning is shown in Equation (14).

(13) $$\begin{array}{c} Pb=\delta *{\left(1-\frac{Iter}{Max\_Iter}\right)}^{2} \end{array}$$

where δ is an important parameter that affects this mechanism, and its analysis and selection will be conducted in the experimental section. Iter and Max_Iter correspond to the current number of iterations and the maximum number of iterations of the algorithm, respectively.

(14) $$\begin{array}{c} X_{i}^{new}=X_{i}+\bar{rand}\cdot \left(\bar{rand}\cdot \left(LB+UB-X_{i}\right)-X_{i}\right) \end{array}$$

where $\bar{rand}$ denotes a random vector with the same dimension as the population, UB denotes the upper bound of the population, and LB represents the lower bound of the population.

Two independent $\bar{rand}$ randomize both the search direction and step size, leading to a more uniform coverage of the search space. This strategy modifies the entire individual vector directly, which can enhance population diversity and help the algorithm escape local optima. Frequent activation may disrupt convergence; however, since it is designed to prevent local optima, its advantages outweigh the disadvantages. Moreover, as this strategy is implemented within the entire collaborative framework, it plays an indispensable role in the framework.

**Figure 1:**
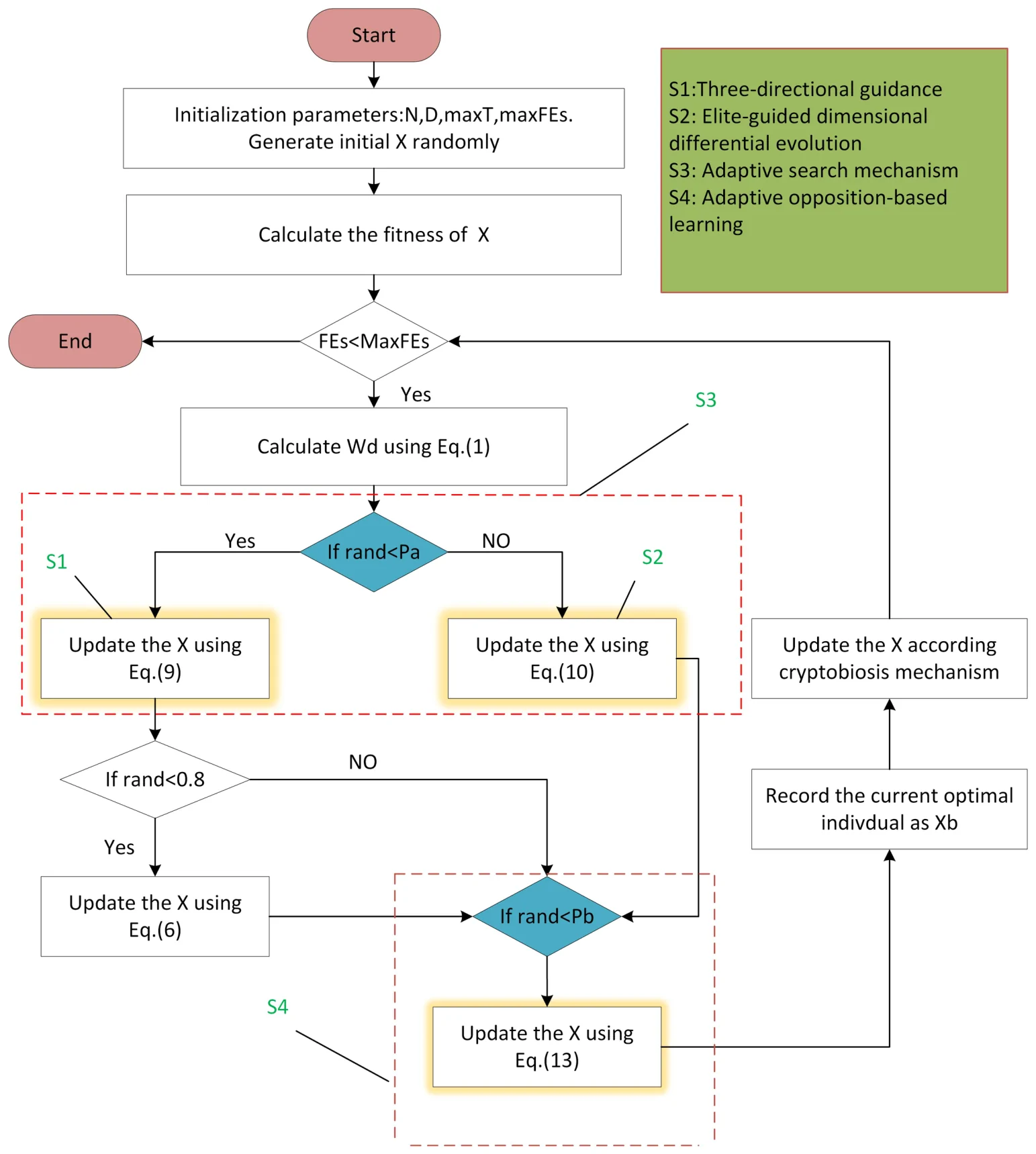
Flowchart of IMGO.

Finally, Table 1 provides the additional parameters of the IMGO for ease of reading and reference. Furthermore, to demonstrate the proposed IMGO framework, the pseudocode is presented in Algorithm 1, and the overall flow chart is shown in Figure 1.

## 4. Experiments and Results

This section mainly focuses on experimental design and result analysis, aiming to verify and analyze the robustness of IMGO through various experiments. To ensure the fairness of the experiment, all these experiments are conducted on the Windows 10 Home Chinese Edition operating system using MATLAB 2024a, with the system equipped with 16 GB RAM and an AMD Ryzen 7 5700U processor (Advanced Micro Devices, Inc., Santa Clara, CA, USA).

### 4.1. Benchmark Function

To validate the performance of the proposed IMGO algorithm, this study employed the CEC2017 benchmark test suite [20] and the CEC2022 benchmark test suite [21] to conduct a systematic evaluation. Comprising real-world optimization problems, mathematical models, and artificially constructed benchmark problems, these test suites exhobit significant complexity and diversity. They comprehensively cover diverse types of optimization scenarios and effectively assess the algorithm’s comprehensive performance in terms of convergence speed, global optimization capability, ability toescape local optima, and capacity to balance exploration and exploitation. The CEC2017 test suite comprises 29 test functions (F2 from the original suite was officially removed due to its instability), which are categorized into unimodal, multimodal, hybrid, and composite functions. The CEC2022 test suite comprises 12 test functions and is designed for research on single-objective real-parameter optimization problems. It features more complex function definitions and evaluation criteria, rendering it well-suited for testing the optimization performance of novel evolutionary algorithms. By comprehensively and efficiently testing these functions, the reliability and effectiveness of the IMGO algorithm are validated.

### 4.2. Parametric Sensitivity Analysis

As noted previously, the values of the two core parameters α and δ in the IMGO algorithm exert significant impacts on the adjustment efficacy of the adaptive opposition-based learning (OBL) mechanism and the optimization performance of the adaptive search mechanism, respectively. To comprehensively assess the optimization performance and robustness of the IMGO algorithm, a systematic sensitivity analysis of the aforementioned parameters is required. In existing literature, only a single or a small number of test functions are typically employed to conduct such analyses. This approach suffers from the limitation of insufficient generalization ability owing to the singular test scenario [22,23]. To mitigate this limitation, this study employs the CEC2017 benchmark test suite as the experimental platform. This test suite encompasses a diverse range of complex optimization problems, enabling a more comprehensive assessment of how parameter variations affect the algorithm’s performance. While controlling computational overhead, the initial value ranges of α and δ are configured as shown in Table 2. Each parameter is assigned 9 candidate values, resulting in a total of 81 parameter combinations for sensitivity testing. The baseline experimental parameters are uniformly configured as follows: population size *N* = 50, $MaxFes=1\times 10^{5}$, Max_Iter = 2000, and Dim = 30. To minimize the impact of randomness on experimental results, each parameter combination is run independently 20 times, and the mean result is adopted as the final performance metric.

**Figure 2:**
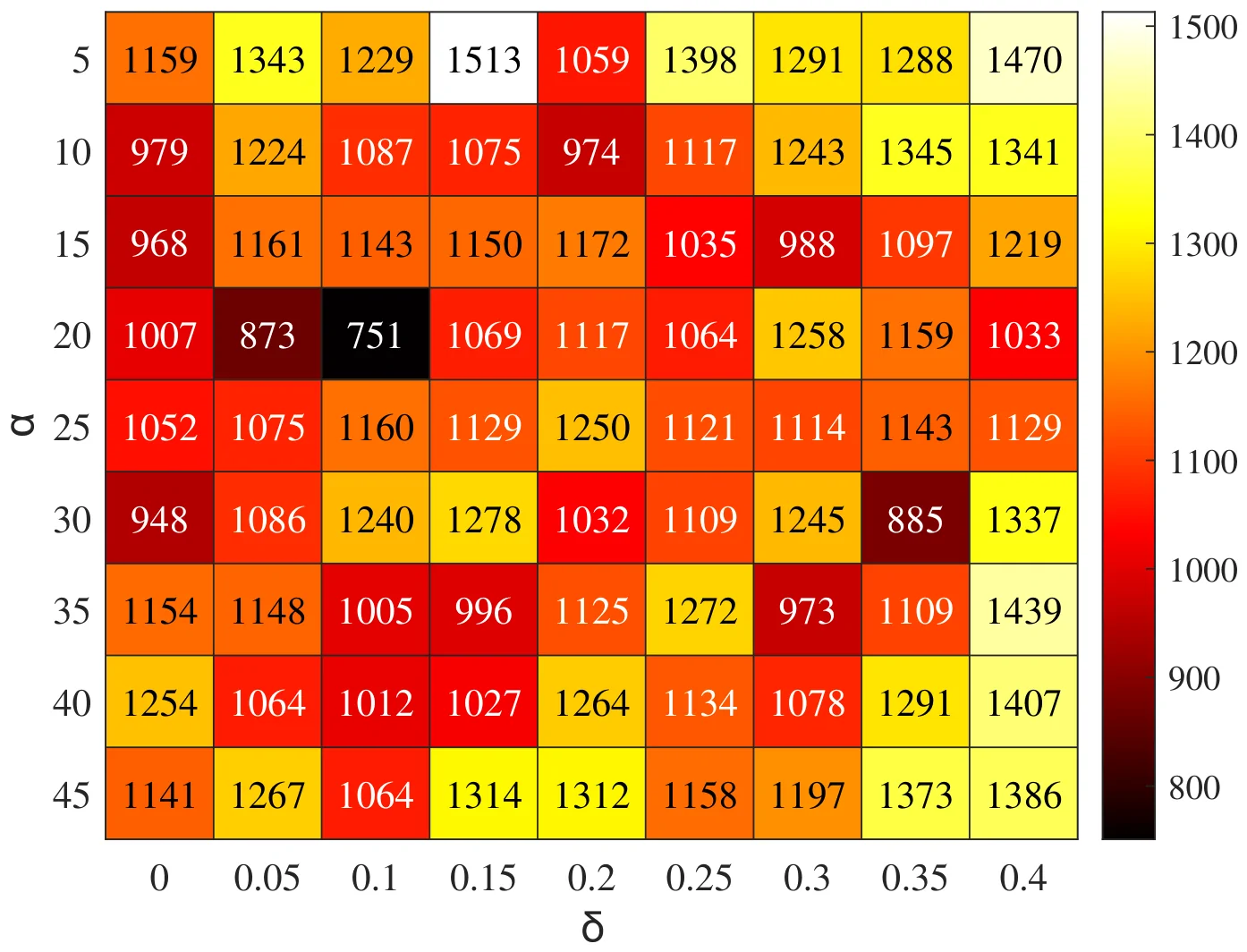
The results of the sensitivity analysis.

As shown in Figure 2, the heatmap intuitively illustrates the performance distribution of the IMGO algorithm across 81 distinct parameter combinations, where each grid cell corresponds to the benchmark test results for a specific parameter configuration. It should be noted that in this experiment, a smaller value denotes superior algorithmic performance. From the heatmap distribution, it can be clearly observed that when δ = 0 (the adaptive opposition-based learning mechanism is not enabled), the algorithm yields generally larger values. However, when δ is held constant and α increases, the algorithm also yields larger values, accompanied by a significant degradation in performance. This phenomenon validates the pivotal role of the adaptive OBL mechanism in IMGO, and its effective activation contributes to enhancing the algorithm’s overall optimization performance. While several parameter combinations also exhibit superior performance, a systematic comparison of the test results across all combinations reveals that the combination of α = 20 and δ = 0.1 enables IMGO to achieve optimal performance. The performance optimum concentrates sharply at a single parameter pair (α=20,δ=0.1), which is relatively uncommon for meta-heuristic optimizers. This characteristic is mainly attributed to the coupling effect of the embedded directional-guidance and adaptive reverse-learning operators in IMGO. Slight deviations of α or δ will break the exploration-exploitation balance, resulting in obvious performance drop. Thus, this parameter combination is tentatively designated as the default configuration for the IMGO algorithm, which serves as the baseline setting for subsequent performance validation and comparative experiments.

### 4.3. Ablation Experiments

**Figure 3:**
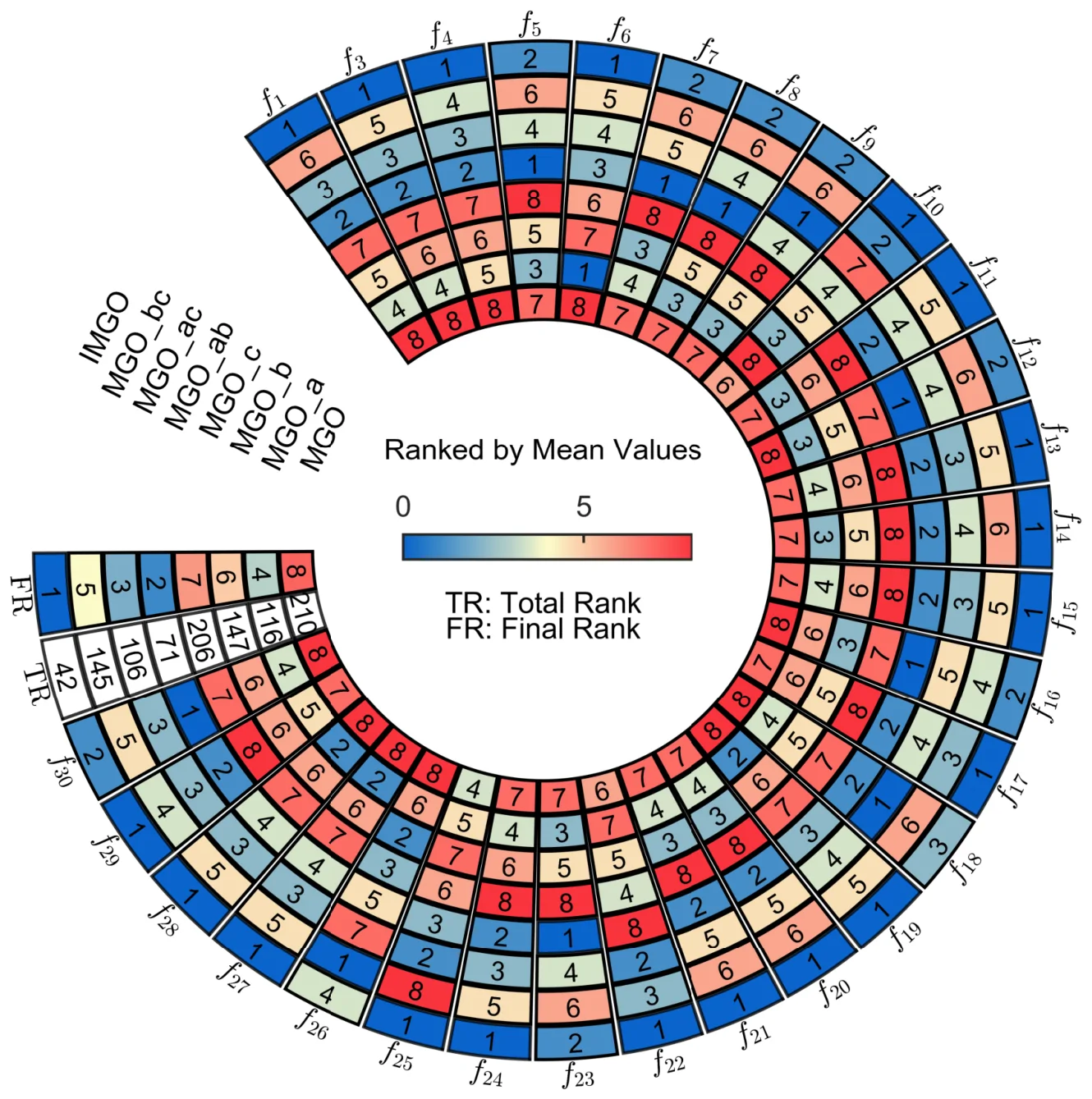
Results of the effectiveness of strategy improvement.

In this study, the four improvement strategies proposed in Section 3 are divided into three mechanisms: a: three-item guidance, b: adaptive search combined with elite-guided differential evolution, and c: adaptive opposition-based learning. These mechanisms are sequentially integrated into the original MGO algorithm to construct six intermediate improved variants, namely MGO_a, MGO_ab, MGO_ac, MGO_b, MGO_bc, and MGO_c. The IMGO algorithm is formed by fully incorporating all three mechanisms, upon which subsequent ablation experiments are carried out. The experiments employ unified parameter settings: population size *N* = 50$,MaxFes=1\times 10^{5}$, Max_Iter = 2000, and problem dimension Dim = 30. Performance evaluation is conducted based on the CEC2017 benchmark test function suite. To minimize the interference of random factors on experimental results, each algorithm is executed independently 30 times, and the mean result is adopted as the benchmark for performance evaluation. Figure 3 illustrates the impact of each improvement strategy on algorithmic performance, presents the rankings of all compared algorithms across all 29 test functions, and provides the statistical results for Total Rank (TR) and Final Rank (FR). The figure is presented as a columnar fan chart, where the value in each cell of the first 29 columns denotes the ascending rank of the mean result of the corresponding algorithm on each test function; TR is defined as the sum of the ranks of the algorithm across all test functions, and FR is derived by sorting TR values in ascending order. It can be observed from the results in Figure 3 that the original MGO achieves a score of 210. All six intermediate variants obtain better scores than MGO, and IMGO yields the optimal score of 42. This indicates that the overall algorithm performance presents a continuous upward trend with the gradual incorporation of improvement mechanisms. Although consistent performance improvement is not observed on individual test functions, the proposed mechanisms exert positive promoting effects on algorithm performance in most cases.

**Figure 4:**
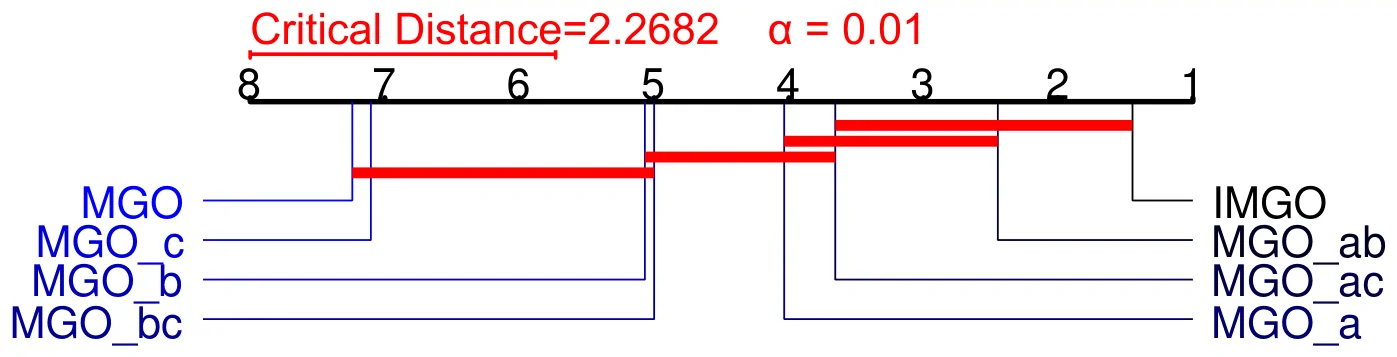
Analysis of strategy.

To conduct a more rigorous evaluation of the effectiveness of the proposed improvement strategies, this study employs the Friedman test [24] to perform statistical significance analysis on the experimental results and plots a critical difference (CD) diagram of the mean ranks of all algorithms (as shown in Figure 4). The Friedman test with a critical-difference (CD=2.2682,α=0.01) is conducted for ablation sensitivity analysis. As shown in Figure 4, variants embedded with mechanism ‘a‘ obtain higher-ranking results. IMGO achieves the best average rank. IMGO has no significant statistical difference compared with MGO_ab, while it exhibits significant superiority against other variants and the original MGO. This diagram intuitively reflects the statistical differences in the mean ranks of different algorithms under the Friedman test. The analysis results demonstrate that with the gradual incorporation of the improvement strategies, the performance of the MGO algorithm exhibits a consistent upward trend; additionally, the finally developed IMGO algorithm is statistically superior to other comparative algorithms, which fully verifies the overall effectiveness of the improvement strategies proposed in this study. In addition, the changes in the average rankings of various algorithms indicate that Mechanism a (three-way guidance) and Mechanism b (adaptive search combined with elite-guided differential evolution) contribute particularly significantly to the performance improvement, demonstrating that the two mechanisms play pivotal roles in the overall improvement framework. The ablation experimental results reveal that each individual mechanism can bring certain performance gains. Nevertheless, neither single-mechanism variants nor partial-combination variants can achieve the performance of the complete IMGO algorithm. Accordingly, Mechanism c (adaptive opposition-based learning) is also indispensable. This confirms that the three mechanisms proposed in this paper can function synergistically and none of them can be omitted for overall performance enhancement.

### 4.4. Qualitative Analysis of IMGO and MGO

**Figure 5:**
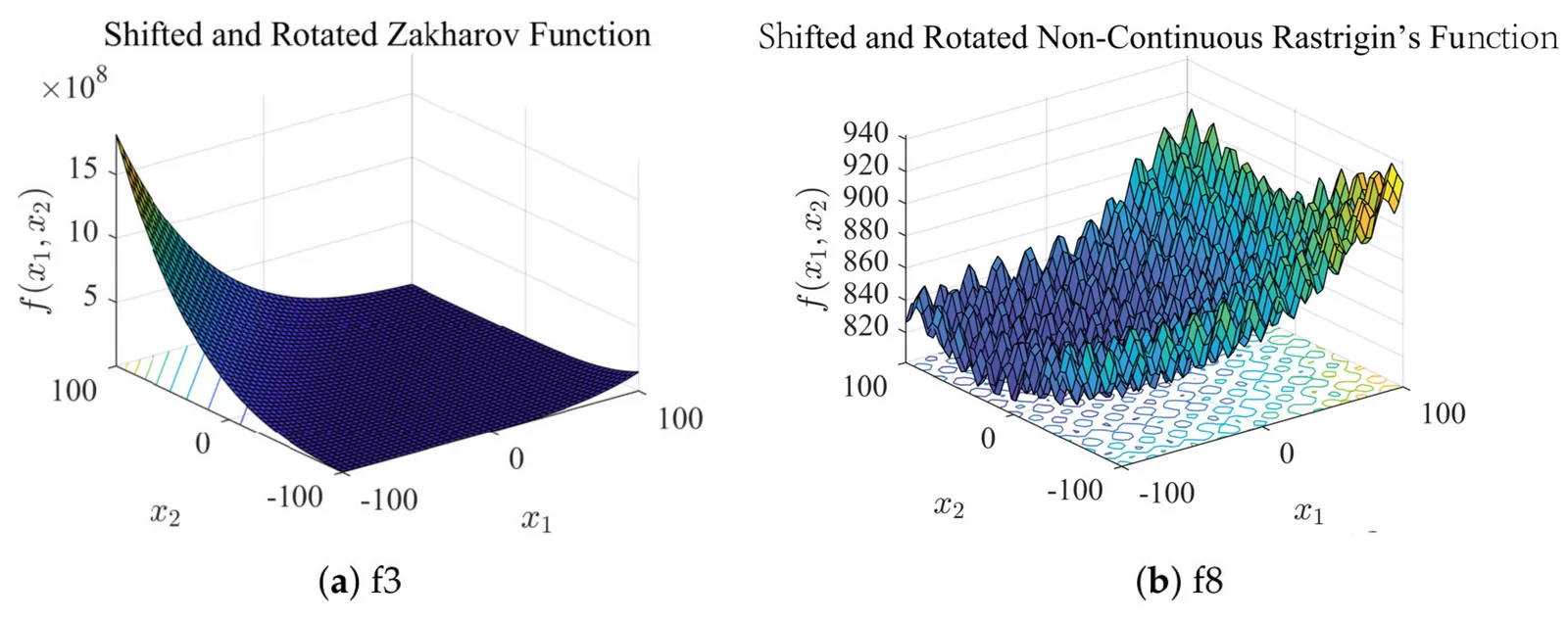
3D figures of f3 and f8.

To more clearly delineate the differences between IMGO and MGO in terms of exploration and exploitation capabilities, this section conducts a qualitative comparison of the two algorithms from the perspectives of search trajectory history, population diversity, and key characteristics of exploration–exploitation balance. Focusing on functional attributes, the analysis employs two benchmark functions from the CEC2017 test suite, namely F3 and F8. F3 is a unimodal function featuring a single global optimum. In contrast, F8 is a multimodal function with a complex multimodal structure and distributed suboptimal regions. This dual-function evaluation framework enables a systematic comparison of algorithm performance across both simple optimization scenarios (represented by F3) and complex optimization scenarios (typified by F8). The images of F3 and F8 are shown in Figure 5.

**Figure 6:**
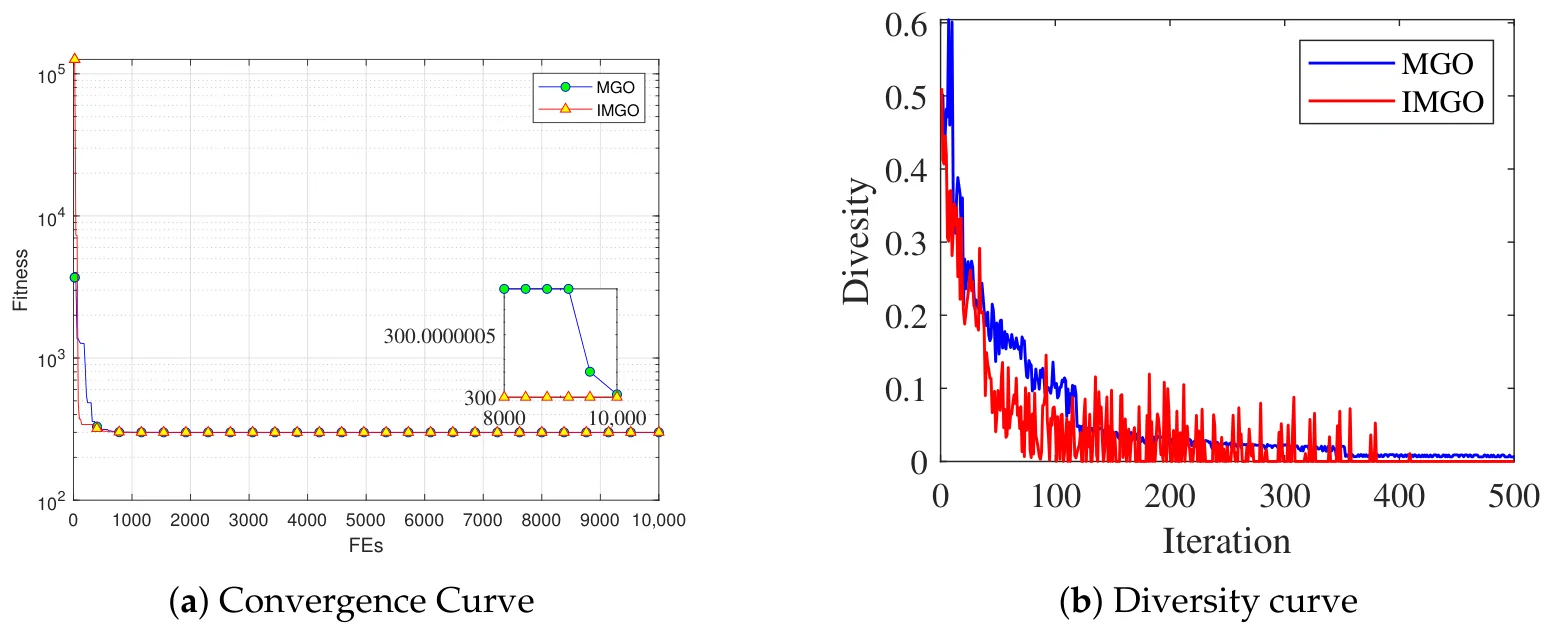

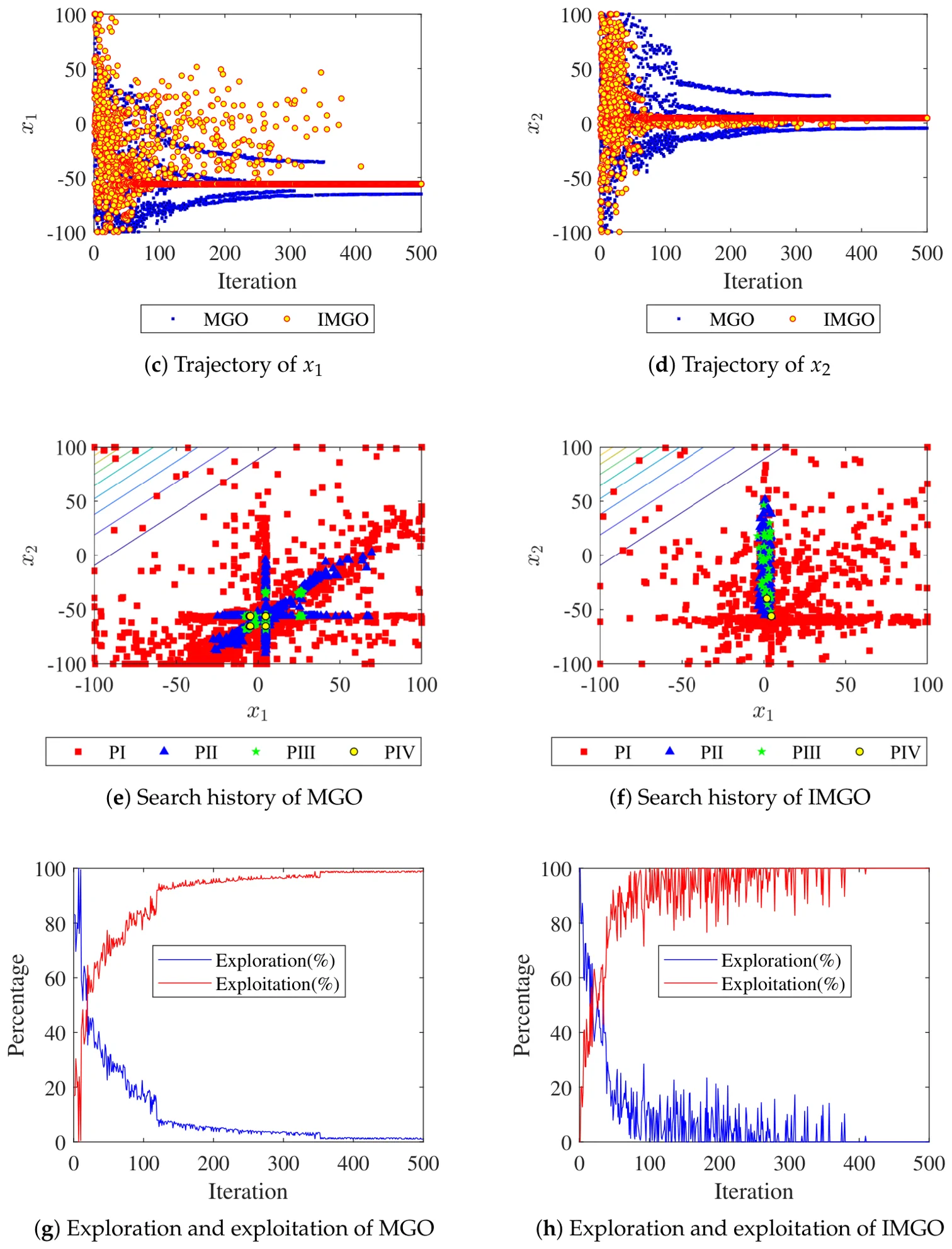
Qualitative comparison on function F3.

**Figure 7:**
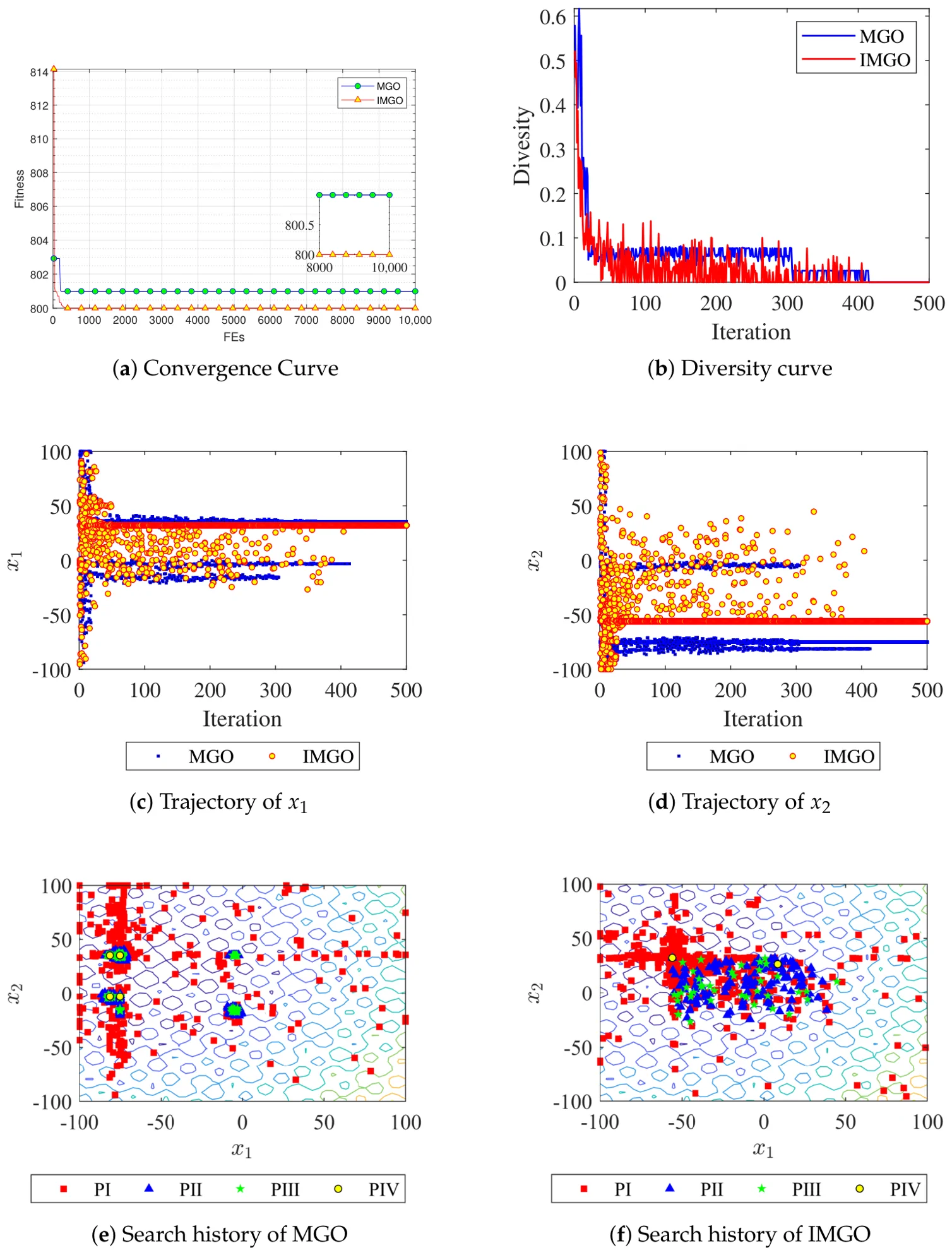

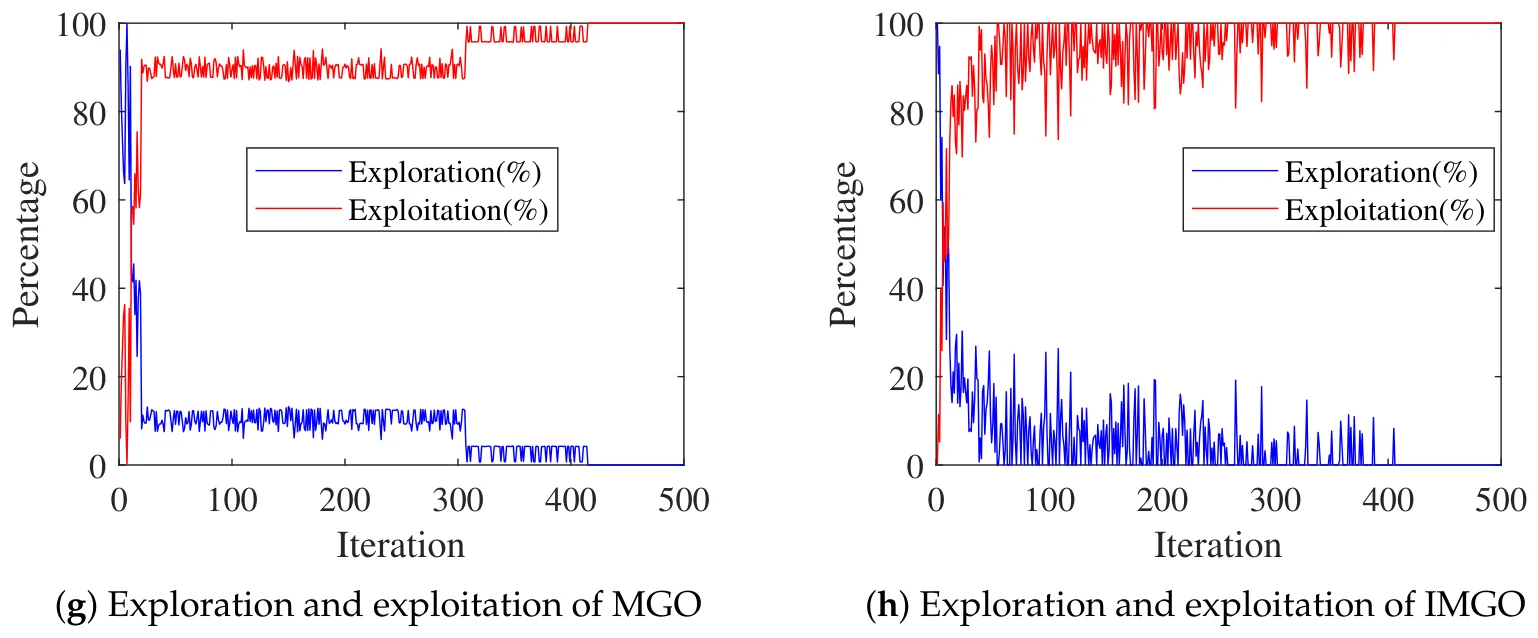
Qualitative comparison on function F8.

To enhance the visualization of results, the dimensions of both F3 and F8 are set to 2. In the experiments, the parameter settings for MGO and IMGO are as follows: *N* = 20, Max_Iter = 500, and $MaxFes=1\times 10^{4}$. Figure 6 and Figure 7 present the comparison results of MGO and IMGO on the F3 and F8 benchmark functions, respectively. The evolutionary process of the algorithms is systematically divided into 4 distinct phases (PI-PIV).

For function F3, as observed in Figure 6a,b, the fitness value of IMGO decreases more rapidly and reaches a lower final value, with both convergence accuracy and speed superior to those of MGO. Meanwhile, IMGO still maintains a certain level of population diversity in the later iterative stage (higher than that of MGO), which is attributed to the OBL mechanism. From Figure 6c,d, IMGO focuses on exploration before 400 iterations and shifts to exploitation after 400 iterations, where the convergence processes of IMGO for X1 and X2 are more compact and stable: it transitions more rapidly from globally scattered exploration to locally stable values, and the final fluctuation is substantially smaller than that of MGO. This demonstrates the precision of the combined search strategy in convergence control and the enhancement of the algorithm’s overall stability. As shown in Figure 6e,f, IMGO enables the population to converge rapidly from the global scope to the optimal region via the three-directional guidance mechanism, characterized by strong concentration and higher search efficiency. In contrast, MGO exhibits low efficiency due to scattered distribution and the absence of rapid focusing capability. From Figure 6g,h, IMGO retains exploration capability continuously and maintains high-intensity local exploitation throughout the entire iterative process. It effectively balances global optimization and local refined search, overcoming the drawback of MGO, which is over-reliance on local exploitation after the completion of early-stage exploration and readily leads to entrapment in local optima. This further confirms IMGO’s comprehensive advantages in convergence performance, population diversity, and variable trajectory control.

For function F8, as illustrated in Figure 7a,b, the IMGO algorithm converges precisely to the target value. In contrast, the MGO algorithm only maintains stability within a fixed interval and fails to reach the optimal solution. Thus, IMGO exhibits higher convergence accuracy while retaining superior population diversity. As observed in Figure 7c,d, during the convergence process of X1 and X2, IMGO achieves wide coverage with scattered distribution, whereas MGO has lower coverage and is prone to converging to local optima. As shown in Figure 7e,f, the historical search trajectories reveal distinct distribution patterns: the search points of IMGO are scattered across multiple regions, whereas those of MGO exhibit high concentration. This broader search scope of IMGO is attributed to its three-directional guidance strategy. In terms of balancing exploration and exploitation, IMGO achieves a dynamic equilibrium between these two core search capabilities. In contrast, MGO prematurely transitions into the exploitation phase, suffers from inadequate exploration capacity, and is thus prone to stagnating in local optima, highlighting the critical role of IMGO’s collaborative search framework. As seen in Figure 7g,h, when MGO undergoes approximately 300 to 400 iterations, its exploitation rate remains above 95% with a relatively low exploration rate. In contrast, IMGO’s exploitation rate exhibits significant fluctuations and is lower than that of MGO; however, its exploration rate is comparatively higher. Owing to the adaptive opposition-based learning strategy, IMGO can still maintain population diversity in the later iterative stages. The aforementioned analysis intuitively illustrates IMGO’s advantages in convergence speed, population diversity maintenance, and the balance between exploration and exploitation performance.

### 4.5. IMGO and Quantitative Analysis of Comparison Algorithms

In this section, the proposed IMGO algorithm is quantitatively compared with eight state-of-the-art optimization algorithms: 1. The original baseline Moss Growth Optimization (MGO) [18]. 2. Recently proposed state-of-the-art metaheuristics published from 2022 to 2025 are adopted: Snake Optimizer(SO) [25], Enterprise Development (ED) [26], Phototropic Growth Algorithm (PGA) [27], Triangulation Topology Aggregation Optimizer (TTAO) [28]. 3. Representative improved variants of classic swarm intelligence algorithms are considered:Efficient Improved Grey Wolf Optimizer (HGWO) [29], Efficient Improved Whale Optimization Algorithm(EIWOA) [30] and Efficient Improved Flow Direction Algorithm (CFDA) [31], to verify the performance superiority of IMGO. All comparative algorithms strictly follow the original recommended parameter configurations from their respective publications to guarantee experimental fairness. as shown in Table 3. In this study, IMGO and the comparative algorithms are configured in accordance with the established methodological guidelines (see Table 3). All comparative algorithms adopt a unified population size of *N* = 50 and a maximum of $1\times 10^{5}$ function evaluations, with 30 independent runs conducted for each algorithm on every benchmark function to ensure high credibility of the experimental results. The experiments include 30-dimensional and 50-dimensional benchmark functions from the CEC2017 test suite, as well as 20-dimensional benchmark functions from the CEC2022 test suite, and systematic comparative results are presented. The implementation results are shown in Table 4, Table 5 and Table 6. Four primary quantitative metrics are recorded for performance evaluation: *Mean* (average fitness value over 30 runs), *Std* (standard deviation reflecting result stability), *Best* (optimal fitness), and *Worst* (worst fitness). The reported auxiliary performance metrics are as follows: the “Rank” column indicates the algorithm ranking based on the improved performance values; the “Overall Rank” summarizes the individual rankings across all functions; and the “Final Rank” is determined by summing the “Overall Rank” values. Note that Rank, Overall Rank and Final Rank serve only as auxiliary reference indicators. Our major performance analysis is built upon the raw numerical results including Mean, Std, Best and Worst, rather than aggregate rank scores.

In this experiment, a fixed-population method was used for comparison to ensure that the initial populations were identical, and subsequent experiments were then carried out. The data in Table 4 and Table 5 clearly illustrate that the IMGO algorithm ranks first in both 30-dimensional and 50-dimensional test functions, indicating that IMGO outperforms all comparative algorithms in overall performance. Additionally, IMGO’s “Total Rank” value is significantly lower than that of competing algorithms, further validating its superior performance across different dimensional scenarios. The “Mean” metric, which serves as an indicator of comprehensive algorithm effectiveness, shows that IMGO achieves 19 best results and 4 s-best results in 30 dimensions, and 13 best results and 6 s-best results in 50 dimensions. In contrast, CFDA (the second-ranked algorithm in the 30-dimensional scenario) achieved only 2 best results and 11 s-best results, while CFDA (the second-ranked method in the 50-dimensional scenario) yielded 4 optimal results and 10 suboptimal results. The “Std” metric, which is used to evaluate algorithm stability, reveals that IMGO achieves 17 best results and 4 s-best results in 30 dimensions, and 6 best results and 6 s-best results in 50 dimensions. Similarly, the data in Table 6 clearly illustrate that the IMGO algorithm ranks first among the 20-dimensional test functions in the CEC2022 suite, demonstrating its overall performance superiority over all comparative algorithms. Specifically, IMGO’s “Total Rank” value is 27, which is markedly lower than that of competing algorithms, while the MGO algorithm scores 66 and ranks five. For the mean value metric, IMGO achieves 4 best results and 5 s-best results with no worst results, whereas MGO only secures one second-best result and 3 worst results. Collectively, these findings demonstrate that the IMGO algorithm is significantly superior to other comparative algorithms in both optimization effectiveness and stability.

### 4.6. Statistical Analysis

As can be seen from the experiments in Section 4.5, this experiment employs a fixed-population method for analysis and solves the same test problems, thereby ensuring that the initial population positions of all algorithms are identical. On this basis, the Wilcoxon signed-rank test and the Friedman test [24] are adopted to further verify the superior performance of the IMGO algorithm from a statistical perspective.

#### 4.6.1. Friedman Test

**Figure 8:**
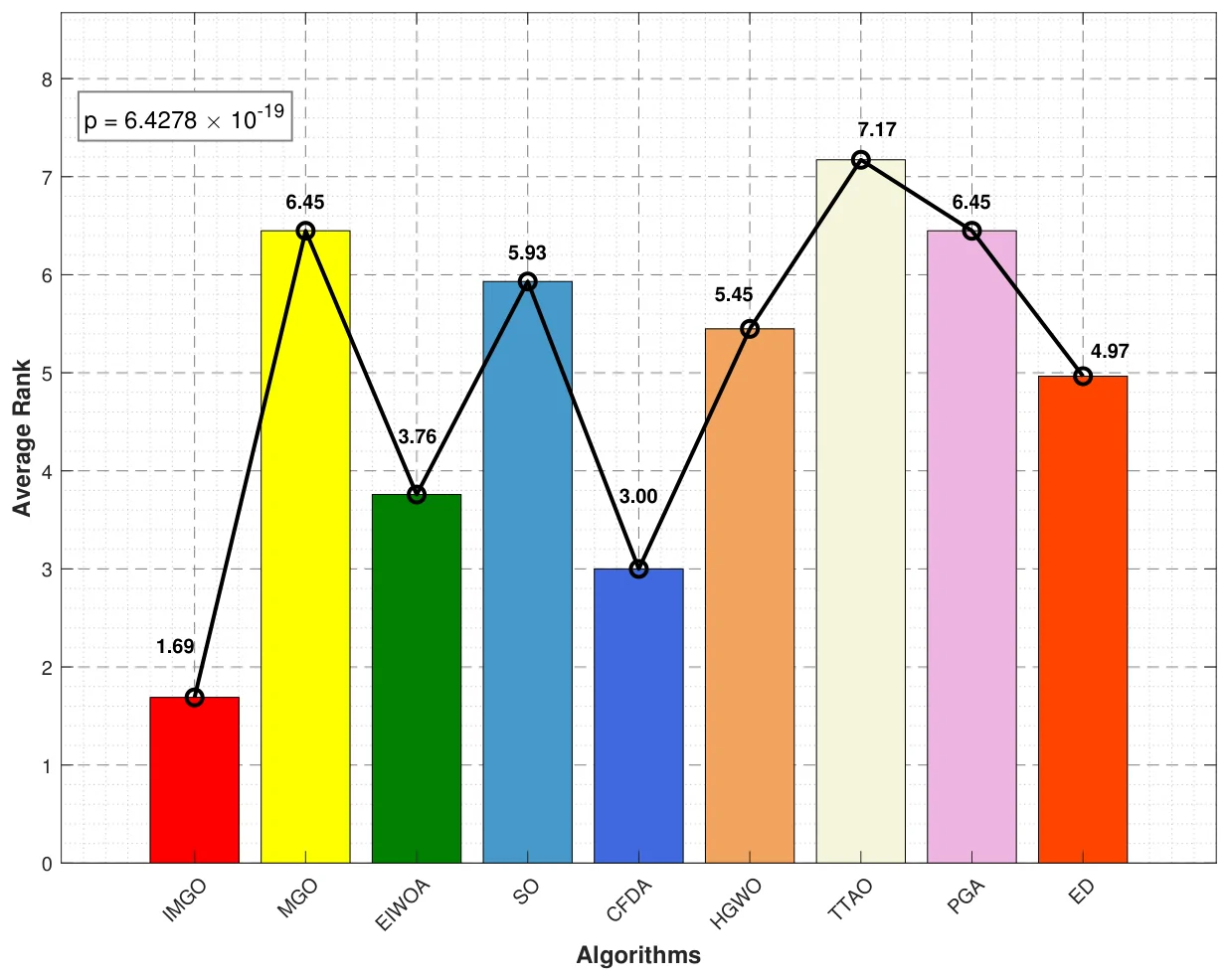
Ranking of average rank results on CEC2017.

First, the Friedman test was performed on the CEC2017 and CEC2022 benchmark suites to assess the statistical performance of all comparative algorithms. Table 7 reports the statistical results for the “Mean”, “Std” (Standard Deviation), and “Best” metrics derived from the Friedman test. All *p*-values are far below the 0.05 significance level (as observed in Table 7), confirming the statistical significance of the experimental results. Further, in the 30-dimensional and 50-dimensional CEC2017 test scenarios, IMGO yielded mean values of 1.6897 and 2.2069, CFDA yielded mean values of 3.0000 and 2.7931, respectively, whereas MGO recorded 6.4483 and 7.3793; for the 20-dimensional CEC2022 scenario, the mean value of IMGO is 2.1364, that of CFDA is 3.8636, and that of MGO is 5.7273. These results clearly demonstrate that the mean value of IMGO is far lower than that of MGO and other variants, and IMGO ranks first in all three metrics, which confirms its comprehensive performance advantages over MGO and other comparative algorithms. Additionally, to enhance the clarity and conciseness of the test results, the average ranks of the nine algorithms across all test functions were calculated to facilitate multiple comparative analyses. IMGO attained an average rank of 1.69, a result that underscores its exceptional performance, with detailed results presented in Figure 8.

#### 4.6.2. Wilcoxon Signed Rank Test

**Figure 9:**
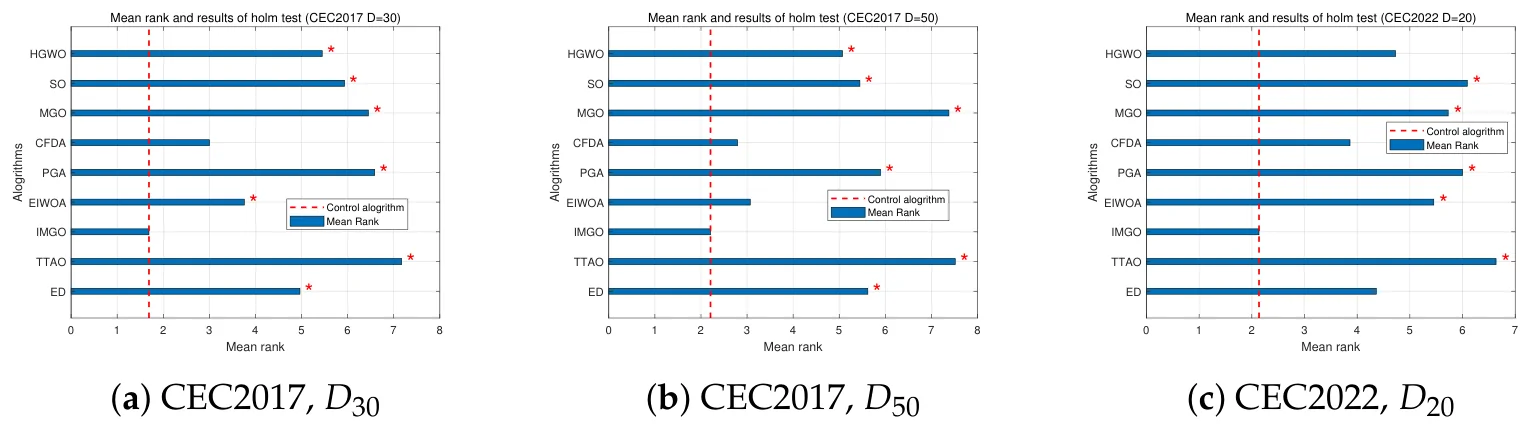
Holm test results.

To further evaluate the statistical significance of the algorithm performance, this study conducted the Wilcoxon signed-rank test (WRST) with a significance level α set to 0.05. Table 8 presents the detailed results of this test across all test functions. Here, ‘R+’ and ‘R−’ denote the sum of ranks for cases where the IMGO algorithm outperforms or underperforms the comparative algorithm, respectively. In the test results, the symbol ‘+’ identifies cases where IMGO is significantly superior to the comparative algorithm, ‘−’ identifies cases where IMGO is significantly inferior, and ‘=’ indicates no significant performance difference between the two algorithms on the given test function. As observed in Table 8, most of the *p*-values from the algorithm comparisons are far below the 0.05 significance level, confirming the statistical validity of the results. Specifically, on the CEC2017 test suite, the 30-dimensional comparison between IMGO and MGO yielded “R+” and “R−” values of 446.00 and 19.00, respectively: IMGO outperformed MGO on 29 benchmark functions did not underperform MGO on any function. In the 50-dimensional scenario, the “R+” and “R−” values were 427.66 and 37.34, respectively, with IMGO outperforming MGO across all 28 benchmark functions. For the CEC2022 test suite, the 20-dimensional comparison between IMGO and MGO resulted in “R+” and “R−” values of 368.58 and 96.42, respectively: IMGO outperformed MGO on 11 benchmark functions and underperformed MGO on only 1 function. Collectively, IMGO demonstrated superior performance to MGO and other comparative algorithms on both the CEC2017 and CEC2022 test functions. The Wilcoxon signed-rank test is only used as auxiliary post-hoc reference rather than sole statistical conclusion. To further validate the IMGO algorithm, a Holm post-hoc test was additionally conducted, with the results shown in Figure 9, the asterisk (*) indicates a statistically significant difference between the corresponding algorithm and the control algorithm. As can be seen from the figure, on the CEC2017 and CEC2022 benchmark suites, the six comparison algorithms exhibit significant performance differences from IMGO, among which the TTAO algorithm performs the worst, further confirming the superiority of IMGO.

Collectively, these findings confirm that the proposed IMGO exhibits statistically significant superiority over MGO and other comparative algorithms in overall performance.

### 4.7. Complexity Analysis

Computational complexity is an important performance metric for optimization algorithms. Thus, we analyze and compare the complexities of MGO and IMGO. The time complexities of the two algorithms are respectively as follows:

$$\begin{array}{cc} O(\mathrm{MGO}) & =O(\mathrm{Initialization})+O(\mathrm{Fitness}\;\mathrm{evaluation})+O(\mathrm{Wind}\;\mathrm{calculation})\\ \; & \;+O(\mathrm{Agent}\;\mathrm{update})+O(\mathrm{Cryptobiosis}\;\mathrm{update})+O(\mathrm{Fitness}\;\mathrm{comparison})\\ \; & =O(ND)+O(C\cdot N)+O(T\cdot D)+O(T\cdot ND)+O(T\cdot ND)+O(T\cdot N)\\ \; & =O\left(ND+C\cdot N+T\cdot D+2T\cdot ND+T\cdot N\right)\\ \; & =O\left((2T+1)ND+(T+C)N+T\cdot D\right)\\ \; & \approx O\left(T\cdot ND+T\cdot C\cdot N\right)\\ \; & =O\left(TN(D+C)\right) \end{array}$$

$$\begin{array}{cc} O(\mathrm{IMGO}) & =O(\mathrm{Initialization})+O(\mathrm{Fitness}\;\mathrm{evaluation})\\ \; & \;+O(\mathrm{Loop}\;\mathrm{iterations}\times (\mathrm{Parameter}\;\mathrm{cal}.+\mathrm{Main}\;\mathrm{updates}\\ \; & \;+\mathrm{Cryptobiosis}\;\mathrm{updates}+\mathrm{Fitness}\;\mathrm{comparisons}))\\ \; & =O\left(ND\right)+O\left(C\cdot N\right)+T\times \left[O(D)+O(ND)+O(ND)\right]\\ \; & =O\left(ND+C\cdot N+T\cdot D+2T\cdot ND+T\cdot N\right)\\ \; & =O\left((2T+1)ND+(T+C)N+T\cdot D\right)\\ \; & \approx O\left(T\cdot ND+T\cdot C\cdot N\right)\\ \; & =O\left(TN(D+C)\right) \end{array}$$

where *N* is the number of mosses, *D* is the dimension, *C* represents the cost of function evaluation, and *T* is the maximum number of iterations. The calculation results of time complexity show that IMGO has the same time complexity O(T×N×(D+C)) as the basic MGO. Although IMGO introduces Pa/Pb probability parameters and multi-strategy update mechanisms, it provides better search performance while maintaining the same complexity level. It is a more sophisticated improved version in algorithm design with equivalent computational efficiency.

### 4.8. Convergence Analysis

**Figure 10:**
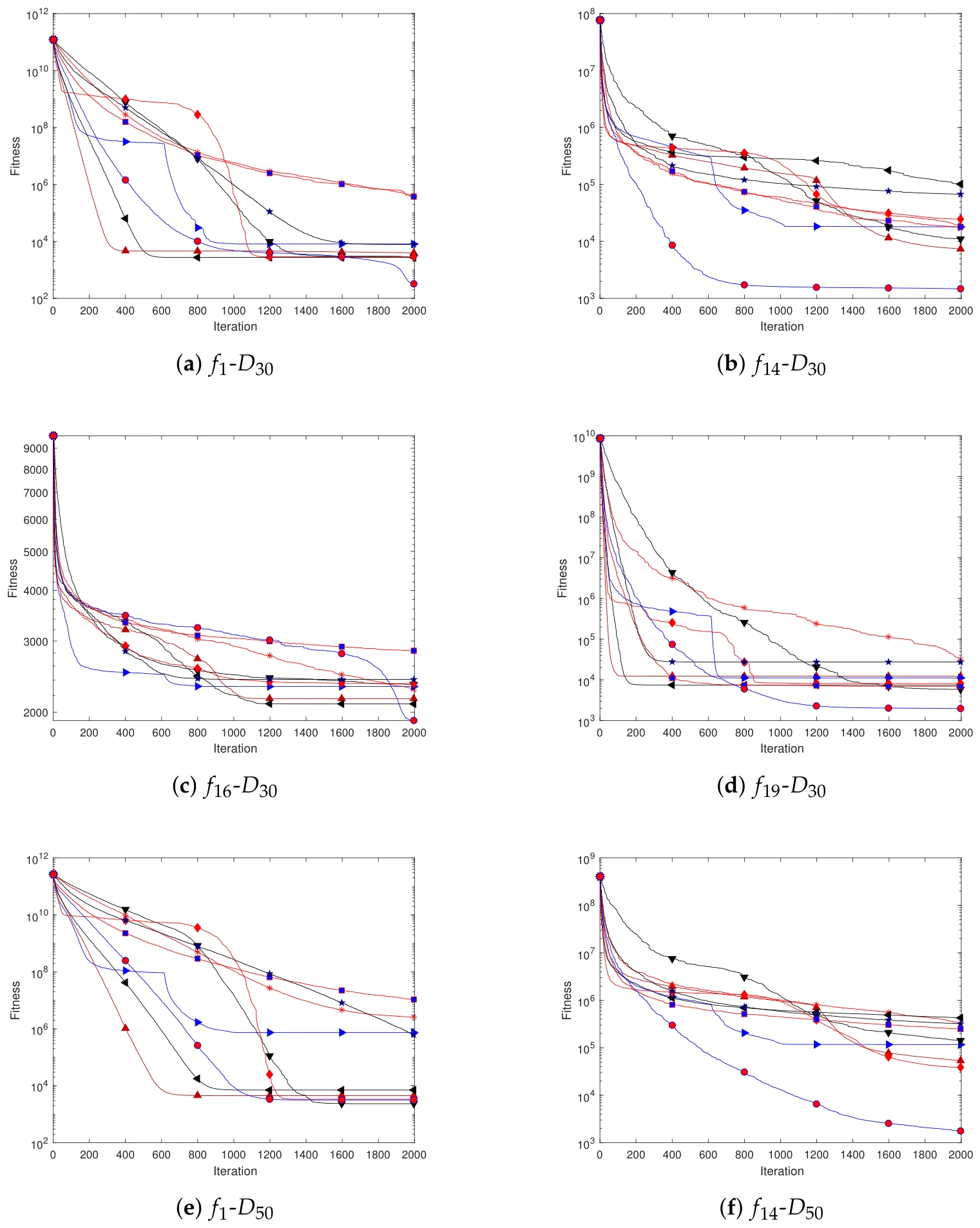

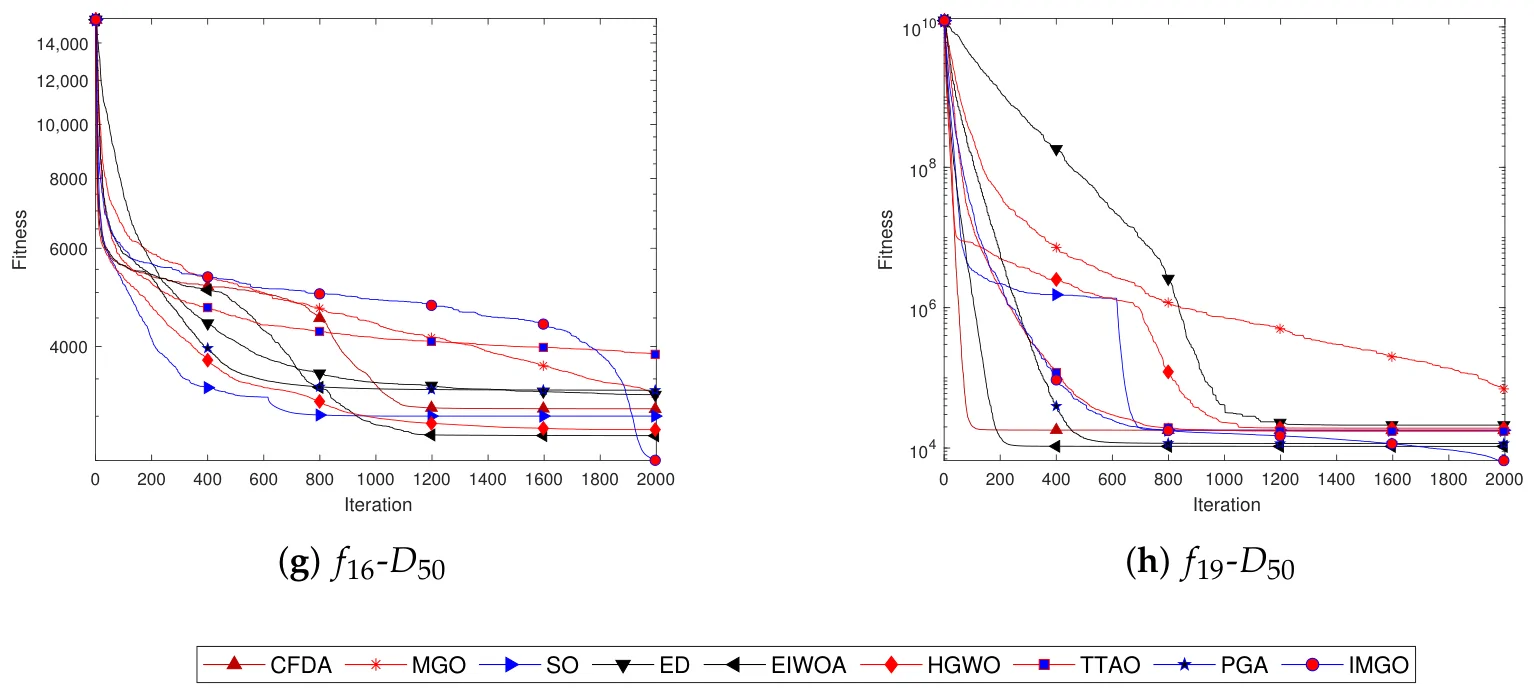
Convergence curves of comparative algorithm on CEC2017.

To compare and analyze the convergence performance of the algorithms, this study selects representative cases randomly from test functions of different types and dimensions, and plots the corresponding convergence curves as shown in Figure 10.

As shown in Figure 10, all algorithms share the same starting point, which demonstrates that under the condition of identical initial populations for all algorithms, IMGO achieves optimal convergence accuracy on most test functions, a finding that aligns fully with the numerical experiment results. Notably for certain 30-dimensional functions, IMGO’s final convergence accuracy is significantly superior to that of MGO and other comparative algorithms, which benefits from the elite-guided dimensional differential evolution strategy. Beyond final accuracy, IMGO also demonstrates distinct advantages in the initial optimization phase: during the early search stage, it achieves a rapid decline in fitness value, with a convergence speed markedly faster than MGO and its improved variants. Furthermore, as observed in Figure 10a,c,h, IMGO maintains continuous convergence behavior even when most comparative algorithms stagnate, owing to the adaptive direction learning strategy, which highlights its robustness against convergence stagnation.

## 5. Application of IMGO

Engineering optimization problems can effectively verify the ability of algorithms to solve practical application problems in real-world scenarios [32]. Constrained optimization problems in the real world are defined as shown in Equation (15).

(15) $$\begin{array}{cc} \mathrm{Minimize}\; & f\left(x\right),\;x=\left(x_{1},x_{2},\ldots ,x_{k}\right)\\ s.t.\; & g_{j}\left(x\right)\leq 0,\;j=1,\ldots ,k\\ \; & h_{p}\left(x\right)=0,\;p=k+1,\ldots ,m \end{array}$$

To comprehensively evaluate the performance of the IMGO algorithm in tackling real-world engineering optimization problems, six representative engineering design problems are chosen as test cases in this section: cantilever stepped beam, stepped conical pulley, planetary gear train design, mixing-pooling-separation, three-level inverter synchronous optimal pulse width modulation design, and solar photovoltaic cell parameter extraction. By leveraging these benchmark problems involving continuous variables, the efficacy and practical applicability of IMGO for solving complex engineering optimization tasks are further validated.

### 5.1. Application Cases

#### 5.1.1. Cantilever Beam Design

**Figure 11:**
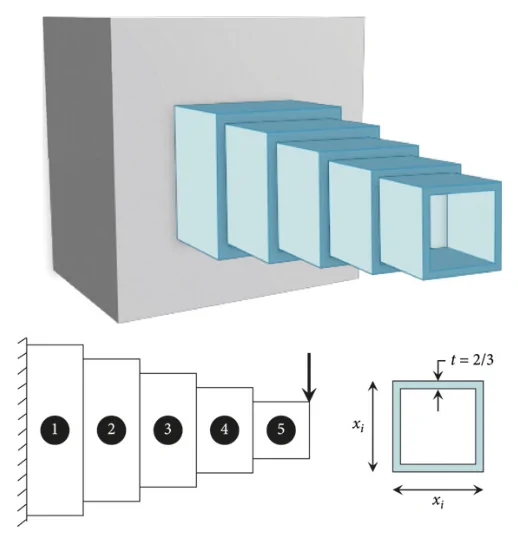
Model structure of Cantilever beam design.

This problem is a structural engineering design example involving the weight optimization of a cantilever beam with a square cross-section. As shown in Figure 11, the structure features the beam with one end rigidly supported, and the free end node of the cantilever subjected to a vertical force. The beam consists of five hollow square elements with constant thickness (①–⑤); the height (or width) of each element is a decision variable, while the thickness remains fixed (valued at 2/3 here). The objective function of this problem is shown in Equation (16); for the specific mathematical model, refer to reference [33].

(16) $$\begin{array}{c} f\left(\bar{x}\right)=0.0624\left(x_{1}+x_{2}+x_{3}+x_{4}+x_{5}\right) \end{array}$$

#### 5.1.2. Step-Cone Pulley

This optimization problem aims to minimize the mass of the four-step conical pulley through five design variables. Among them, four variables represent the diameter of each step respectively, and the other variable is the width of the pulley. The problem includes a total of 11 nonlinear constraints, which are used to ensure that the transmission power is stably maintained at 0.75 horsepower. The objective function of this problem is shown in Equation (17); for the specific mathematical model, refer to reference [34].

(17) $$\begin{array}{c} f\left(\bar{x}\right)=\rho \omega [d_{1}^{2}\left\{11+{\left(\frac{N_{1}}{N}\right)}^{2}\right\}+d_{2}^{2}\left\{1+{\left(\frac{N_{2}}{N}\right)}^{2}\right\}\\ +d_{3}^{2}\left\{1+{\left(\frac{N_{3}}{N}\right)}^{2}\right\}+d_{4}^{2}\left\{1+{\left(\frac{N_{4}}{N}\right)}^{2}\right\}] \end{array}$$

#### 5.1.3. Planetary Gear Train Design

The core objective of this optimization problem is to minimize the maximum error of the vehicle transmission ratio, and based on this objective function, solve for the optimal total tooth number configuration of the automatic planetary transmission system. The objective function of this problem is shown in Equation (18); for the specific mathematical model, refer to reference [35].

(18) $$\begin{array}{c} f\left(\bar{x}\right)=\max\left|i_{k}-i_{0k}\right|,\;k=\left\{1,2,\ldots ,R\right\} \end{array}$$

#### 5.1.4. Blending-Pooling-Separation

This problem involves a feed mixture with three components, which is separated into two multi-component outputs using separators, as well as separation/mixing/confluence units. The operating cost of each separator depends linearly on the separator’s flow rate, with constraints based on mass balance relationships of individual separators, splitters, and mixers. The objective function of this problem is shown in Equation (19); for the specific mathematical model, refer to reference [36].

(19) $$\begin{array}{c} f\left(\bar{x}\right)=0.9979+0.00432x_{5}+0.01517x_{13} \end{array}$$

#### 5.1.5. SOPWM for 3-Level Inverters

**Figure 12:**
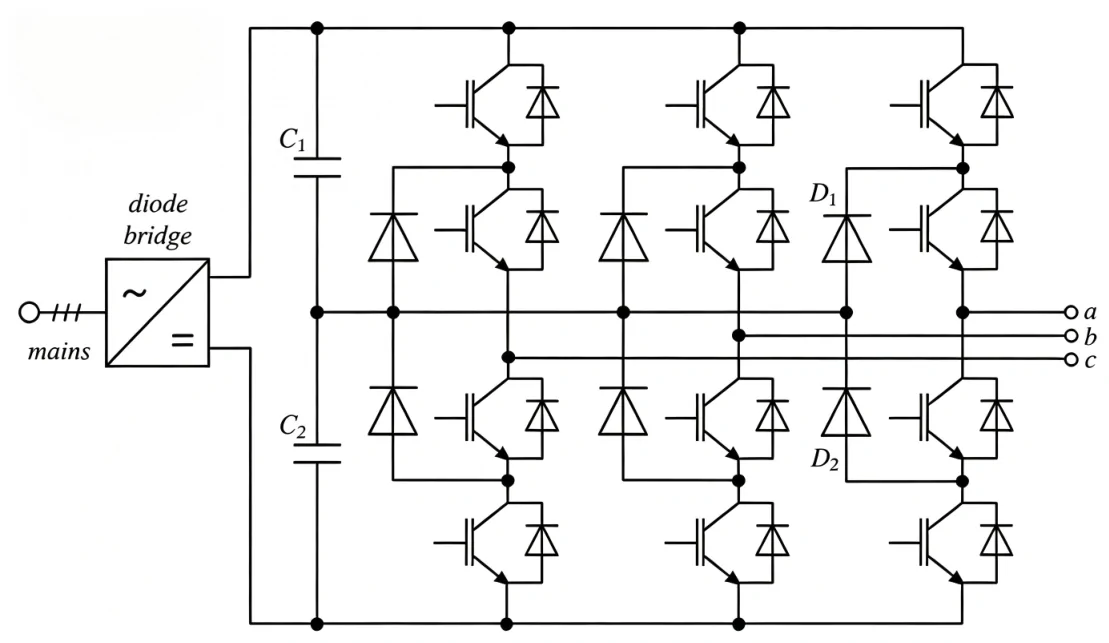
Model structure of SOPWM.

Synchronous Optimal Pulse Width Modulation (SOPWM) is an advanced modulation strategy suitable for medium-voltage drives. This method significantly reduces the switching frequency without increasing the waveform distortion. This characteristic effectively reduces switching losses, thereby improving the overall operating efficiency of the inverter. The SOPWM technology can complete the optimal calculation of switching angles within a single fundamental cycle, and while minimizing current distortion, it exhibits technical characteristics that can be used as a Scalable Carrier Optimization Policy (COP). The objective function of the SOPWM problem is shown in Equation (20), and its structure is shown in Figure 12; for the specific mathematical model, refer to reference [37].

(20) $$\begin{array}{c} f=\frac{\sqrt{\sum_{k}k^{-4}\sum_{l=1}^{M}{\left(i\cos(ka_{l})\right)}^{2}}}{\sqrt{\sum_{k}k^{-4}}} \end{array}$$

#### 5.1.6. Photovoltaic Parameter Extraction (SDM)

**Figure 13:**
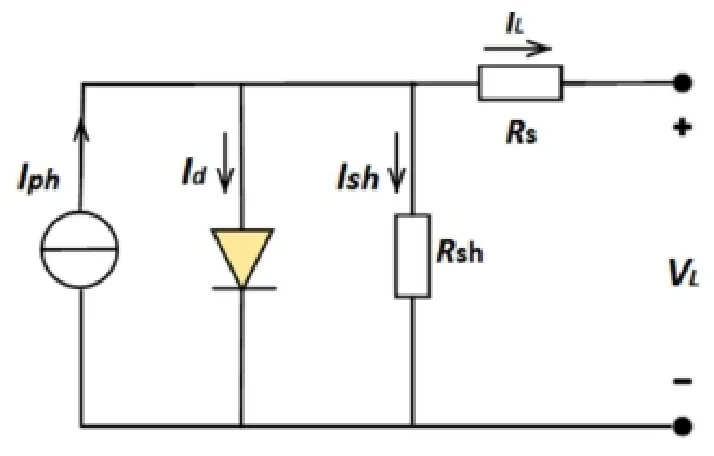
Model structure of SDM.

Parameter extraction of solar photovoltaic cells is a highly nonlinear and complex real-world problem. In general industrial applications, the single-diode model (SDM) is simple in calculation and widely used. The equivalent circuit of the SDM is shown in Figure 13, which consists of a current source in parallel with a diode, a shunt resistor representing leakage current, the arrows indicate the directions of the currents in the single-diode equivalent circuit model, and a series resistor accounting for losses related to load current [38]. The objective function of this problem is shown in Equation (21); for the specific mathematical model, refer to Reference [39].

(21) $$\begin{array}{c} \mathrm{RMSE}\left(X\right)=\sqrt{\frac{1}{M}\sum_{i=1}^{M}f_{k}{\left(V_{L},I,X\right)}^{2}} \end{array}$$

Table 9 outlines the key characteristics of the aforementioned optimization problem: D denotes the total number of decision variables, g represents the number of inequality constraints, and h denotes the number of equality constraints.

### 5.2. Analysis and Discussion of the Experimental Results

For the comparative evaluation of algorithm performance across these eight engineering problems, the initial parameter configurations of all algorithms matched those employed in the prior experiments.

#### 5.2.1. Numerical Analysis

Experimental results from 30 independent runs of each algorithm are tabulated in Table 10. As observed in Table 10, across tests on different engineering design problems, IMGO exhibits excellent global optimization capability. Its optimization accuracy consistently ranks among the top of all comparative algorithms, achieving optimal performance in 6 test cases. Additionally, the ‘std’ value of IMGO across multiple independent runs is lower than that of other algorithms, indicating smaller result fluctuations than most comparative algorithms and demonstrating outstanding stability, enabling it to reliably output high-quality solutions in practical applications. Notably, IMGO’s performance advantages are not dependent on specific types of test functions; it maintains leading performance in optimization problems with distinct characteristics, reflecting the algorithm’s excellent generalization ability and comprehensive performance, which are significantly superior to those of other comparative algorithms. IMGO ranks first in comprehensive performance, while the ED and SO algorithms also demonstrate strong capabilities in addressing engineering optimization problems.

**Figure 14:**
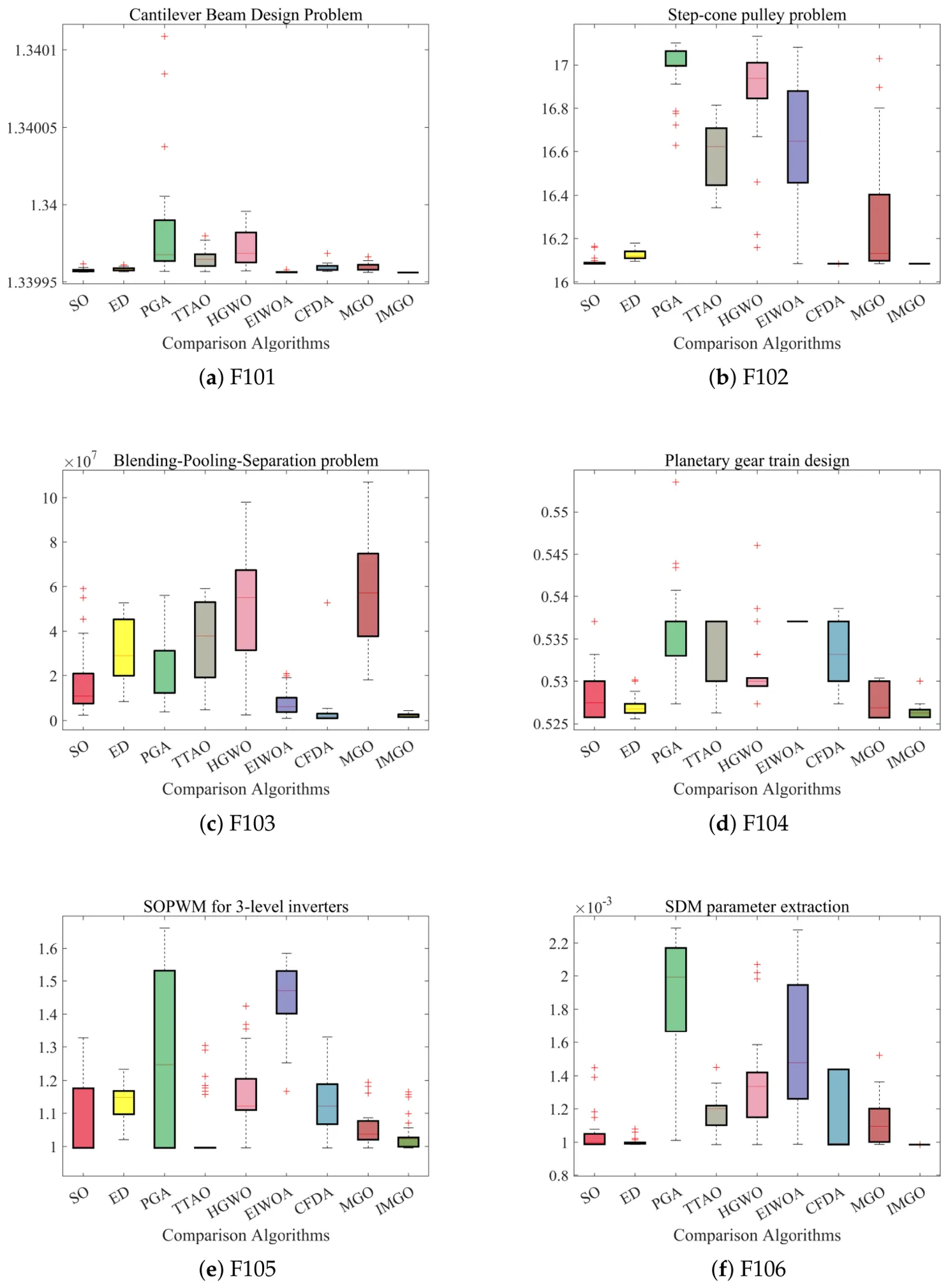
Box plot of engineering case results.

To more intuitively demonstrate algorithm performance in engineering cases, the corresponding box plot results are presented in Figure 14. The dashed whiskers represent the data range, and the red plus signs (+) indicate outliers. A box plot appearing as a straight line indicates low result volatility, meaning the algorithm achieved the theoretical optimal value across 30 independent runs. It can be clearly observed that IMGO achieves theoretically optimal performance in most application scenarios and exhibits significant performance advantages over the MGO algorithm and other comparative algorithms.

#### 5.2.2. Statistical Analysis of the Engineering Cases

To further quantitatively evaluate the performance differences among all competitors on real-world engineering optimization problems, we carry out the Friedman omnibus test and Wilcoxon signed-rank pairwise test, as summarized in Table 11 and Table 12.

As shown in Table 11, the Friedman test yields a *p*-value of $4.1116\times 10^{-4}$, which is far below the significance threshold of 0.05. This result confirms that there exist statistically significant performance distinctions across these algorithms. The proposed IMGO achieves the best average rank of 4.9447 and obtains the first rank among all compared algorithms including the original MGO. The pairwise Wilcoxon signed-rank test outcomes are given in Table 12. IMGO exhibits statistically significant superiority over SO, ED, PGA, TTAO, HGWO, EIWOA and the original MGO (p<0.05). When compared with CFDA, the *p*-value equals 0.163, higher than 0.05, which indicates no statistically significant difference between IMGO and CFDA for the selected real-life engineering benchmarks. Nevertheless, IMGO still possesses a better average Friedman rank. From the +/=/− statistics in Table 12, IMGO achieves the largest count of winning cases with very few losing instances. These observations further demonstrate that the improved mechanisms embedded in IMGO are effective for practical engineering-oriented optimization tasks.

## 6. Conclusions and Future Work

To mitigate the critical limitations premature convergence and easy trapping in local optima of the MGO algorithm, this paper proposes an IMGO algorithm. IMGO integrates four core enhancement strategies to address these limitations: the three-directional guidance strategy expands the search space via multi-matrix collaboration, establishing a robust foundation for global exploration; elite-guided dimensional differential evolution enhances local exploitation precision, forming a synergistic complement to the three-directional guidance strategy; the adaptive search mechanism dynamically balances the exploration-exploitation trade-off to improve convergence efficiency; and adaptive opposition-based learning augments population diversity, enabling the algorithm to escape local optima and refine IMGO’s overall optimization framework. Together, these strategies achieve an effective balance between exploration and exploitation, reduce premature convergence by increasing population diversity, and facilitate escape from local optima, thereby elevating search accuracy. A comprehensive performance evaluation was conducted by comparing IMGO with the original MGO and seven representative state-of-the-art algorithms. Extensive experiments on the CEC2017 and CEC2022 benchmark suites demonstrate that IMGO outperforms the eight comparative algorithms significantly in both solution quality and convergence accuracy, across varying test function dimensions and convergence curve analyses. Further statistical validation using the Friedman test and Wilcoxon signed-rank test confirms that IMGO exhibits statistically significant superiority over all comparative algorithms. Validation on eight real-world engineering design problems demonstrates that the proposed IMGO algorithm is highly effective for solving complex design optimization problems. The strategy proposed in this paper can provide useful references for the design of other algorithms and lay a foundation for the development of more efficient metaheuristic algorithms or variants of existing algorithms.

While the IMGO algorithm delivers strong overall performance and shows promise for addressing optimization tasks, it still presents certain inherent limitations. Specifically, it lacks superior convergence speed in the early iterative phase, which necessitates further refinement to satisfy the demand for enhanced computational efficiency. Furthermore, IMGO’s validation has been confined to single-objective optimization problems, and its applicability and efficacy in multi-objective optimization contexts remain unexamined. In future research, leveraging the core characteristics of the IMGO algorithm, we will develop more efficient MGO variants and extend their application to diverse domains, including image processing, path planning, machine learning model parameter tuning, and multi-objective optimization, to augment their practical utility. In future research, we will transplant the four enhanced strategies (three-directional guidance, elite-guided dimensional differential evolution, adaptive search mechanism, and adaptive opposition-based learning) to other baseline meta-heuristic algorithms, so as to further verify the universality and generalizability of these improved components. Moreover, further comparative experiments with classic CEC competition algorithms such as jSO will be carried out in follow-up work to enrich the benchmark comparison pool.

## Figures and Tables

**Table 1:** Key parameters of IMGO.

Parameters	Feasible Possible Intervals	Explanation
α	5~50	Control the value of Pa size
θ	0~2 or vector from 0 to 1	Three directional guidance step size control
δ	0~0.5	Control the value of Pb size
*Pa*	0~1	Adaptive search control parameters
*Pb*	0~1	Adaptive reverse learning control parameters

**Table 2:** Specific values for parameters α and δ.

Parameter	Specific Values								
δ	0	0.05	0.10	0.15	0.20	0.25	0.30	0.35	0.40
α	5	10	15	20	25	30	35	40	45

**Table 3:** Parameters settings of compared algorithms.

No	Algorithm	Parameters	Reference and Year
1	SO	*NM* = *NF* = 0.5*N*, *C*1 = 0.5, Threshold(food) = 0.25, Threshold(temp) = 0.25	[25], 2022
2	ED	*P*1 = 0.1	[26], 2024
3	PGA	α = exp(−t/T)	[27], 2025
4	TTAO	*l* = 9 × exp(−t/T)	[28], 2024
5	HGWO	*S* = 25, *L* = 5	[29], 2023
6	EIWOA	α = 1.5, β = 0.025	[30], 2023
7	CFDA	λ = 1.35, μ = 4	[31], 2024
8	MGO	rec_num = 10, *w* = 2, *d*1 = 0.2.	[18], 2024
9	IMGO	δ = 0.1, α = 20	Present

**Table 4:** Statistical Results of the 30-Dimensional Scenario (CEC2017).

f(x)	Index	SO	ED	PGA	TTAO	HGWO	EIWOA	CFDA	MGO	IMGO
f1	Mean	$8.1237\times 10^{3}$	$2.8365\times 10^{3}$	$7.8149\times 10^{3}$	$1.0705\times 10^{4}$	$2.8955\times 10^{3}$	$2.7367\times 10^{3}$	$3.9630\times 10^{3}$	$3.8513\times 10^{5}$	$3.2483\times 10^{2}$
	Std	$7.4343\times 10^{3}$	$3.6191\times 10^{3}$	$7.5582\times 10^{3}$	$1.8820\times 10^{4}$	$2.8095\times 10^{3}$	$2.0596\times 10^{3}$	$3.9384\times 10^{3}$	$3.5251\times 10^{5}$	$4.9777\times 10^{2}$
	Best	$1.2116\times 10^{2}$	$1.0131\times 10^{2}$	$2.0725\times 10^{2}$	$1.8333\times 10^{2}$	$1.0634\times 10^{2}$	$1.0287\times 10^{2}$	$1.0565\times 10^{2}$	$2.6237\times 10^{4}$	$1.0005\times 10^{2}$
	worst	$2.3657\times 10^{4}$	$1.5324\times 10^{4}$	$2.0934\times 10^{4}$	$7.6185\times 10^{4}$	$9.4206\times 10^{3}$	$5.5041\times 10^{3}$	$1.4445\times 10^{4}$	$1.2667\times 10^{6}$	$2.4012\times 10^{3}$
	Rank	7	3	6	8	4	2	5	9	1
f3	Mean	$3.8948\times 10^{4}$	$5.0915\times 10^{4}$	$4.9154\times 10^{4}$	$1.6911\times 10^{4}$	$3.2661\times 10^{2}$	$1.7759\times 10^{4}$	$3.3079\times 10^{2}$	$7.1190\times 10^{4}$	$1.4502\times 10^{3}$
	Std	$1.0292\times 10^{4}$	$1.1801\times 10^{4}$	$1.1736\times 10^{4}$	$4.8641\times 10^{3}$	$5.2715\times 10^{1}$	$5.3771\times 10^{3}$	$7.0493\times 10^{1}$	$1.2619\times 10^{4}$	$6.9875\times 10^{2}$
	Best	$1.9889\times 10^{4}$	$2.6471\times 10^{4}$	$2.0656\times 10^{4}$	$8.9185\times 10^{3}$	$3.0003\times 10^{2}$	$1.1707\times 10^{4}$	$3.0002\times 10^{2}$	$4.2812\times 10^{4}$	$6.0533\times 10^{2}$
	worst	$6.5149\times 10^{4}$	$7.7986\times 10^{4}$	$7.1985\times 10^{4}$	$2.8138\times 10^{4}$	$4.9685\times 10^{2}$	$3.5051\times 10^{4}$	$6.1745\times 10^{2}$	$1.0108\times 10^{5}$	$3.1207\times 10^{3}$
	Rank	6	8	7	4	1	5	2	9	3
f4	Mean	$4.8666\times 10^{2}$	$4.6155\times 10^{2}$	$4.9405\times 10^{2}$	$4.9189\times 10^{2}$	$5.1299\times 10^{2}$	$4.9059\times 10^{2}$	$4.7913\times 10^{2}$	$4.9460\times 10^{2}$	$4.5364\times 10^{2}$
	Std	$1.7955\times 10^{1}$	$2.6760\times 10^{1}$	$1.3181\times 10^{1}$	$1.9311\times 10^{1}$	$2.1843\times 10^{1}$	$2.7395\times 10^{1}$	$1.9333\times 10^{1}$	$1.9694\times 10^{1}$	$2.8801\times 10^{1}$
	Best	$4.4340\times 10^{2}$	$4.0399\times 10^{2}$	$4.7165\times 10^{2}$	$4.6811\times 10^{2}$	$4.6812\times 10^{2}$	$4.0046\times 10^{2}$	$4.0073\times 10^{2}$	$4.3203\times 10^{2}$	$4.0009\times 10^{2}$
	worst	$5.1928\times 10^{2}$	$4.9949\times 10^{2}$	$5.2316\times 10^{2}$	$5.3949\times 10^{2}$	$5.7669\times 10^{2}$	$5.7464\times 10^{2}$	$5.1343\times 10^{2}$	$5.2567\times 10^{2}$	$4.8875\times 10^{2}$
	Rank	4	2	7	6	9	5	3	8	1
f5	Mean	$5.5646\times 10^{2}$	$6.0088\times 10^{2}$	$5.8074\times 10^{2}$	$6.6154\times 10^{2}$	$5.4302\times 10^{2}$	$5.3701\times 10^{2}$	$5.3868\times 10^{2}$	$5.7613\times 10^{2}$	$5.3425\times 10^{2}$
	Std	$1.3095\times 10^{1}$	$1.4689\times 10^{1}$	$2.6194\times 10^{1}$	$3.1258\times 10^{1}$	$1.1901\times 10^{1}$	$1.0306\times 10^{1}$	$1.1014\times 10^{1}$	$1.2931\times 10^{1}$	$8.8258\times 10^{0}$
	Best	$5.3188\times 10^{2}$	$5.5445\times 10^{2}$	$5.4179\times 10^{2}$	$5.9117\times 10^{2}$	$5.2288\times 10^{2}$	$5.1890\times 10^{2}$	$5.2288\times 10^{2}$	$5.5371\times 10^{2}$	$5.2191\times 10^{2}$
	worst	$5.9056\times 10^{2}$	$6.2789\times 10^{2}$	$6.4128\times 10^{2}$	$7.4216\times 10^{2}$	$5.6965\times 10^{2}$	$5.6066\times 10^{2}$	$5.5956\times 10^{2}$	$6.0466\times 10^{2}$	$5.5876\times 10^{2}$
	Rank	5	8	7	9	4	2	3	6	1
f6	Mean	$6.0119\times 10^{2}$	$6.0008\times 10^{2}$	$6.0010\times 10^{2}$	$6.2859\times 10^{2}$	$6.0731\times 10^{2}$	$6.0000\times 10^{2}$	$6.0009\times 10^{2}$	$6.0000\times 10^{2}$	$6.0000\times 10^{2}$
	Std	$1.0136\times 10^{0}$	$8.6228\times 10^{-2}$	$3.8664\times 10^{-1}$	$1.0159\times 10^{1}$	$2.3997\times 10^{0}$	$7.6117\times 10^{-14}$	$7.1813\times 10^{-2}$	$2.5908\times 10^{-3}$	$1.8848\times 10^{-4}$
	Best	$6.0023\times 10^{2}$	$6.0000\times 10^{2}$	$6.0000\times 10^{2}$	$6.1244\times 10^{2}$	$6.0353\times 10^{2}$	$6.0000\times 10^{2}$	$6.0001\times 10^{2}$	$6.0000\times 10^{2}$	$6.0000\times 10^{2}$
	worst	$6.0486\times 10^{2}$	$6.0047\times 10^{2}$	$6.0211\times 10^{2}$	$6.5337\times 10^{2}$	$6.1287\times 10^{2}$	$6.0000\times 10^{2}$	$6.0022\times 10^{2}$	$6.0001\times 10^{2}$	$6.0000\times 10^{2}$
	Rank	7	4	6	9	8	1	5	3	2
f7	Mean	$8.1118\times 10^{2}$	$8.3337\times 10^{2}$	$8.0195\times 10^{2}$	$8.5938\times 10^{2}$	$7.8970\times 10^{2}$	$7.7051\times 10^{2}$	$7.6518\times 10^{2}$	$8.1619\times 10^{2}$	$7.6504\times 10^{2}$
	Std	$2.6526\times 10^{1}$	$1.2420\times 10^{1}$	$2.4184\times 10^{1}$	$3.5743\times 10^{1}$	$2.2918\times 10^{1}$	$1.5595\times 10^{1}$	$1.0248\times 10^{1}$	$1.2949\times 10^{1}$	$9.0151\times 10^{0}$
	Best	$7.6753\times 10^{2}$	$8.0463\times 10^{2}$	$7.6351\times 10^{2}$	$8.0172\times 10^{2}$	$7.5917\times 10^{2}$	$7.5121\times 10^{2}$	$7.4865\times 10^{2}$	$7.7712\times 10^{2}$	$7.5212\times 10^{2}$
	worst	$8.7413\times 10^{2}$	$8.6273\times 10^{2}$	$8.6498\times 10^{2}$	$9.2367\times 10^{2}$	$8.7903\times 10^{2}$	$8.3205\times 10^{2}$	$7.9609\times 10^{2}$	$8.4356\times 10^{2}$	$7.8284\times 10^{2}$
	Rank	6	8	5	9	4	3	2	7	1
f8	Mean	$8.5758\times 10^{2}$	$9.0228\times 10^{2}$	$8.7884\times 10^{2}$	$9.3662\times 10^{2}$	$8.4195\times 10^{2}$	$8.3984\times 10^{2}$	$8.4019\times 10^{2}$	$8.8163\times 10^{2}$	$8.3628\times 10^{2}$
	Std	$1.1444\times 10^{1}$	$1.3735\times 10^{1}$	$2.3222\times 10^{1}$	$2.9015\times 10^{1}$	$1.4946\times 10^{1}$	$9.3019\times 10^{0}$	$1.1344\times 10^{1}$	$1.5423\times 10^{1}$	$1.0470\times 10^{1}$
	Best	$8.3287\times 10^{2}$	$8.7096\times 10^{2}$	$8.3781\times 10^{2}$	$8.9280\times 10^{2}$	$8.1890\times 10^{2}$	$8.1990\times 10^{2}$	$8.1592\times 10^{2}$	$8.5582\times 10^{2}$	$8.1994\times 10^{2}$
	worst	$8.8360\times 10^{2}$	$9.2507\times 10^{2}$	$9.2735\times 10^{2}$	$9.9790\times 10^{2}$	$8.8557\times 10^{2}$	$8.6368\times 10^{2}$	$8.6794\times 10^{2}$	$9.1548\times 10^{2}$	$8.6170\times 10^{2}$
	Rank	5	8	6	9	4	2	3	7	1
f9	Mean	$1.1480\times 10^{3}$	$9.8538\times 10^{2}$	$9.1424\times 10^{2}$	$2.0236\times 10^{3}$	$1.1418\times 10^{3}$	$9.0000\times 10^{2}$	$9.0019\times 10^{2}$	$9.6749\times 10^{2}$	$9.0031\times 10^{2}$
	Std	$1.7558\times 10^{2}$	$1.0433\times 10^{2}$	$3.1745\times 10^{1}$	$9.9971\times 10^{2}$	$9.5179\times 10^{1}$	$9.9020\times 10^{-14}$	$4.0546\times 10^{-1}$	$5.6267\times 10^{1}$	$1.0105\times 10^{0}$
	Best	$9.0525\times 10^{2}$	$9.0817\times 10^{2}$	$9.0009\times 10^{2}$	$1.0044\times 10^{3}$	$9.9319\times 10^{2}$	$9.0000\times 10^{2}$	$9.0000\times 10^{2}$	$9.1010\times 10^{2}$	$9.0000\times 10^{2}$
	worst	$1.5667\times 10^{3}$	$1.3896\times 10^{3}$	$1.0697\times 10^{3}$	$5.1256\times 10^{3}$	$1.3303\times 10^{3}$	$9.0000\times 10^{2}$	$9.0182\times 10^{2}$	$1.1524\times 10^{3}$	$9.0554\times 10^{2}$
	Rank	8	6	4	9	7	1	2	5	3
f10	Mean	$3.0555\times 10^{3}$	$3.8540\times 10^{3}$	$4.6220\times 10^{3}$	$6.0116\times 10^{3}$	$4.1335\times 10^{3}$	$3.4363\times 10^{3}$	$4.0721\times 10^{3}$	$5.1866\times 10^{3}$	$3.8827\times 10^{3}$
	Std	$4.2657\times 10^{2}$	$2.5117\times 10^{2}$	$7.7086\times 10^{2}$	$3.4847\times 10^{2}$	$7.7699\times 10^{2}$	$6.0756\times 10^{2}$	$5.0450\times 10^{2}$	$3.4618\times 10^{2}$	$6.3959\times 10^{2}$
	Best	$2.1372\times 10^{3}$	$3.1705\times 10^{3}$	$2.5909\times 10^{3}$	$5.0012\times 10^{3}$	$2.6620\times 10^{3}$	$2.1821\times 10^{3}$	$2.8972\times 10^{3}$	$4.4779\times 10^{3}$	$2.4773\times 10^{3}$
	worst	$4.0361\times 10^{3}$	$4.3232\times 10^{3}$	$5.8107\times 10^{3}$	$6.8219\times 10^{3}$	$5.4780\times 10^{3}$	$4.5751\times 10^{3}$	$4.9555\times 10^{3}$	$6.1540\times 10^{3}$	$5.1300\times 10^{3}$
	Rank	1	3	7	9	6	2	5	8	4
f11	Mean	$1.2126\times 10^{3}$	$1.1664\times 10^{3}$	$1.1641\times 10^{3}$	$1.2099\times 10^{3}$	$1.2001\times 10^{3}$	$1.1540\times 10^{3}$	$1.1422\times 10^{3}$	$1.2088\times 10^{3}$	$1.1380\times 10^{3}$
	Std	$4.7980\times 10^{1}$	$3.1028\times 10^{1}$	$3.2624\times 10^{1}$	$3.6991\times 10^{1}$	$3.3884\times 10^{1}$	$3.0146\times 10^{1}$	$2.9552\times 10^{1}$	$2.4560\times 10^{1}$	$2.6928\times 10^{1}$
	Best	$1.1360\times 10^{3}$	$1.1245\times 10^{3}$	$1.1197\times 10^{3}$	$1.1513\times 10^{3}$	$1.1570\times 10^{3}$	$1.1085\times 10^{3}$	$1.1029\times 10^{3}$	$1.1482\times 10^{3}$	$1.1057\times 10^{3}$
	worst	$1.3380\times 10^{3}$	$1.2431\times 10^{3}$	$1.2276\times 10^{3}$	$1.2828\times 10^{3}$	$1.2881\times 10^{3}$	$1.1884\times 10^{3}$	$1.1831\times 10^{3}$	$1.2582\times 10^{3}$	$1.1882\times 10^{3}$
	Rank	9	5	4	8	6	3	2	7	1
f12	Mean	$3.5481\times 10^{5}$	$6.9378\times 10^{5}$	$9.1678\times 10^{5}$	$6.8494\times 10^{5}$	$1.0987\times 10^{5}$	$4.7630\times 10^{5}$	$4.6500\times 10^{4}$	$1.7146\times 10^{6}$	$1.7498\times 10^{4}$
	Std	$4.2853\times 10^{5}$	$3.5574\times 10^{5}$	$9.6466\times 10^{5}$	$5.9389\times 10^{5}$	$1.7462\times 10^{5}$	$3.3451\times 10^{5}$	$4.1200\times 10^{4}$	$1.2950\times 10^{6}$	$1.0773\times 10^{4}$
	Best	$3.0580\times 10^{4}$	$7.6091\times 10^{4}$	$2.3475\times 10^{4}$	$6.8869\times 10^{4}$	$1.2732\times 10^{4}$	$5.8654\times 10^{4}$	$6.9645\times 10^{3}$	$2.5025\times 10^{5}$	$2.6442\times 10^{3}$
	worst	$2.1711\times 10^{6}$	$1.2807\times 10^{6}$	$3.4329\times 10^{6}$	$1.9972\times 10^{6}$	$9.3687\times 10^{5}$	$1.5463\times 10^{6}$	$2.1802\times 10^{5}$	$5.9825\times 10^{6}$	$3.9917\times 10^{4}$
	Rank	4	7	8	6	3	5	2	9	1
f13	Mean	$1.7171\times 10^{4}$	$1.5680\times 10^{4}$	$1.9591\times 10^{4}$	$1.2302\times 10^{4}$	$1.8495\times 10^{4}$	$2.7935\times 10^{4}$	$1.5388\times 10^{4}$	$7.5058\times 10^{4}$	$5.1697\times 10^{3}$
	Std	$1.3562\times 10^{4}$	$9.8156\times 10^{3}$	$1.8234\times 10^{4}$	$9.0357\times 10^{3}$	$1.7476\times 10^{4}$	$1.3578\times 10^{4}$	$1.6979\times 10^{4}$	$5.7697\times 10^{4}$	$4.0168\times 10^{3}$
	Best	$2.9987\times 10^{3}$	$1.7882\times 10^{3}$	$1.4322\times 10^{3}$	$2.0815\times 10^{3}$	$3.4508\times 10^{3}$	$7.2813\times 10^{3}$	$1.4438\times 10^{3}$	$1.2917\times 10^{4}$	$1.5683\times 10^{3}$
	worst	$5.5580\times 10^{4}$	$4.6164\times 10^{4}$	$5.1031\times 10^{4}$	$2.9671\times 10^{4}$	$7.2310\times 10^{4}$	$5.9944\times 10^{4}$	$6.2336\times 10^{4}$	$2.5618\times 10^{5}$	$1.5785\times 10^{4}$
	Rank	5	4	7	2	6	8	3	9	1
f14	Mean	$1.8020\times 10^{4}$	$1.1028\times 10^{4}$	$6.7377\times 10^{4}$	$1.2221\times 10^{4}$	$2.4635\times 10^{4}$	$1.0151\times 10^{5}$	$7.3378\times 10^{3}$	$1.8569\times 10^{4}$	$1.4747\times 10^{3}$
	Std	$1.4731\times 10^{4}$	$1.2944\times 10^{4}$	$5.4893\times 10^{4}$	$9.3591\times 10^{3}$	$4.2888\times 10^{4}$	$1.1687\times 10^{5}$	$4.8674\times 10^{3}$	$1.3911\times 10^{4}$	$2.0684\times 10^{1}$
	Best	$1.9054\times 10^{3}$	$2.0419\times 10^{3}$	$5.7483\times 10^{3}$	$2.4587\times 10^{3}$	$2.6000\times 10^{3}$	$2.9314\times 10^{3}$	$1.8163\times 10^{3}$	$2.9831\times 10^{3}$	$1.4407\times 10^{3}$
	worst	$5.4752\times 10^{4}$	$5.5095\times 10^{4}$	$1.8392\times 10^{5}$	$4.2275\times 10^{4}$	$2.0223\times 10^{5}$	$4.8437\times 10^{5}$	$1.9010\times 10^{4}$	$5.6807\times 10^{4}$	$1.5144\times 10^{3}$
	Rank	5	3	8	4	7	9	2	6	1
f15	Mean	$9.2225\times 10^{3}$	$2.6065\times 10^{3}$	$1.8951\times 10^{4}$	$4.4106\times 10^{3}$	$5.0214\times 10^{3}$	$3.6030\times 10^{3}$	$1.0179\times 10^{4}$	$4.0489\times 10^{4}$	$1.6405\times 10^{3}$
	Std	$5.8758\times 10^{3}$	$1.5892\times 10^{3}$	$1.4564\times 10^{4}$	$3.4146\times 10^{3}$	$3.6926\times 10^{3}$	$3.7677\times 10^{3}$	$8.6991\times 10^{3}$	$2.9997\times 10^{4}$	$6.0829\times 10^{1}$
	Best	$2.0585\times 10^{3}$	$1.5604\times 10^{3}$	$1.5476\times 10^{3}$	$1.6993\times 10^{3}$	$1.6183\times 10^{3}$	$1.5476\times 10^{3}$	$1.5718\times 10^{3}$	$4.1485\times 10^{3}$	$1.5492\times 10^{3}$
	worst	$2.4462\times 10^{4}$	$8.2937\times 10^{3}$	$4.2756\times 10^{4}$	$1.4569\times 10^{4}$	$1.6987\times 10^{4}$	$2.0285\times 10^{4}$	$3.4628\times 10^{4}$	$1.2345\times 10^{5}$	$1.7732\times 10^{3}$
	Rank	6	2	8	4	5	3	7	9	1
f16	Mean	$2.3149\times 10^{3}$	$2.3428\times 10^{3}$	$2.4100\times 10^{3}$	$2.7209\times 10^{3}$	$2.3538\times 10^{3}$	$2.0966\times 10^{3}$	$2.1601\times 10^{3}$	$2.2740\times 10^{3}$	$1.9067\times 10^{3}$
	Std	$2.1015\times 10^{2}$	$1.5598\times 10^{2}$	$3.0322\times 10^{2}$	$2.5941\times 10^{2}$	$2.3744\times 10^{2}$	$2.0326\times 10^{2}$	$2.2342\times 10^{2}$	$1.4083\times 10^{2}$	$1.3773\times 10^{2}$
	Best	$1.8426\times 10^{3}$	$1.9958\times 10^{3}$	$1.6591\times 10^{3}$	$2.1169\times 10^{3}$	$1.7257\times 10^{3}$	$1.7327\times 10^{3}$	$1.6325\times 10^{3}$	$1.9656\times 10^{3}$	$1.6385\times 10^{3}$
	worst	$2.8185\times 10^{3}$	$2.6255\times 10^{3}$	$2.9155\times 10^{3}$	$3.1987\times 10^{3}$	$2.8196\times 10^{3}$	$2.4918\times 10^{3}$	$2.5999\times 10^{3}$	$2.4822\times 10^{3}$	$2.1445\times 10^{3}$
	Rank	5	6	8	9	7	2	3	4	1
f17	Mean	$2.0467\times 10^{3}$	$1.9395\times 10^{3}$	$2.0599\times 10^{3}$	$2.0020\times 10^{3}$	$1.8647\times 10^{3}$	$1.9155\times 10^{3}$	$1.8340\times 10^{3}$	$1.9124\times 10^{3}$	$1.7866\times 10^{3}$
	Std	$1.5732\times 10^{2}$	$8.3187\times 10^{1}$	$1.4132\times 10^{2}$	$1.1030\times 10^{2}$	$8.3147\times 10^{1}$	$1.5181\times 10^{2}$	$1.2112\times 10^{2}$	$4.7556\times 10^{1}$	$2.2397\times 10^{1}$
	Best	$1.7623\times 10^{3}$	$1.7834\times 10^{3}$	$1.7529\times 10^{3}$	$1.7513\times 10^{3}$	$1.7635\times 10^{3}$	$1.7289\times 10^{3}$	$1.7254\times 10^{3}$	$1.8338\times 10^{3}$	$1.7453\times 10^{3}$
	worst	$2.3304\times 10^{3}$	$2.0774\times 10^{3}$	$2.3456\times 10^{3}$	$2.2698\times 10^{3}$	$2.0167\times 10^{3}$	$2.2784\times 10^{3}$	$2.1151\times 10^{3}$	$1.9996\times 10^{3}$	$1.8256\times 10^{3}$
	Rank	8	6	9	7	3	5	2	4	1
f18	Mean	$2.3658\times 10^{5}$	$3.6810\times 10^{5}$	$7.5395\times 10^{5}$	$2.2590\times 10^{5}$	$8.2321\times 10^{4}$	$6.3678\times 10^{5}$	$5.1991\times 10^{4}$	$3.2904\times 10^{5}$	$1.2659\times 10^{4}$
	Std	$2.0940\times 10^{5}$	$1.7788\times 10^{5}$	$8.1572\times 10^{5}$	$1.2584\times 10^{5}$	$4.1580\times 10^{4}$	$5.1614\times 10^{5}$	$2.4044\times 10^{4}$	$1.6078\times 10^{5}$	$1.0751\times 10^{4}$
	Best	$2.8966\times 10^{4}$	$7.9930\times 10^{4}$	$7.8622\times 10^{4}$	$4.0158\times 10^{4}$	$2.4845\times 10^{4}$	$1.1072\times 10^{5}$	$2.0123\times 10^{4}$	$5.5249\times 10^{4}$	$2.3868\times 10^{3}$
	worst	$9.8737\times 10^{5}$	$7.2696\times 10^{5}$	$3.4855\times 10^{6}$	$5.0543\times 10^{5}$	$1.7812\times 10^{5}$	$1.9189\times 10^{6}$	$1.2874\times 10^{5}$	$7.8729\times 10^{5}$	$4.6293\times 10^{4}$
	Rank	5	7	9	4	3	8	2	6	1
f19	Mean	$1.1179\times 10^{4}$	$5.8324\times 10^{3}$	$2.7452\times 10^{4}$	$6.8449\times 10^{3}$	$8.1174\times 10^{3}$	$7.4422\times 10^{3}$	$1.2241\times 10^{4}$	$2.9107\times 10^{4}$	$1.9737\times 10^{3}$
	Std	$1.2132\times 10^{4}$	$2.8810\times 10^{3}$	$2.2535\times 10^{4}$	$6.4115\times 10^{3}$	$7.6625\times 10^{3}$	$5.8191\times 10^{3}$	$1.2303\times 10^{4}$	$2.3876\times 10^{4}$	$3.3915\times 10^{1}$
	Best	$2.0179\times 10^{3}$	$2.0032\times 10^{3}$	$1.9181\times 10^{3}$	$1.9712\times 10^{3}$	$2.2256\times 10^{3}$	$1.9482\times 10^{3}$	$1.9502\times 10^{3}$	$3.4886\times 10^{3}$	$1.9288\times 10^{3}$
	worst	$5.3844\times 10^{4}$	$1.3996\times 10^{4}$	$5.6419\times 10^{4}$	$3.3137\times 10^{4}$	$3.6784\times 10^{4}$	$2.6904\times 10^{4}$	$4.3611\times 10^{4}$	$1.0813\times 10^{5}$	$2.0581\times 10^{3}$
	Rank	6	2	8	3	5	4	7	9	1
f20	Mean	$2.3397\times 10^{3}$	$2.3059\times 10^{3}$	$2.3781\times 10^{3}$	$2.4819\times 10^{3}$	$2.2653\times 10^{3}$	$2.1805\times 10^{3}$	$2.2577\times 10^{3}$	$2.2894\times 10^{3}$	$2.1483\times 10^{3}$
	Std	$1.4883\times 10^{2}$	$8.5412\times 10^{1}$	$2.3854\times 10^{2}$	$1.2890\times 10^{2}$	$9.2824\times 10^{1}$	$9.9865\times 10^{1}$	$1.3433\times 10^{2}$	$6.8375\times 10^{1}$	$4.6216\times 10^{1}$
	Best	$2.0669\times 10^{3}$	$2.1334\times 10^{3}$	$2.0386\times 10^{3}$	$2.3088\times 10^{3}$	$2.1010\times 10^{3}$	$2.0229\times 10^{3}$	$2.1407\times 10^{3}$	$2.1167\times 10^{3}$	$2.0784\times 10^{3}$
	worst	$2.6588\times 10^{3}$	$2.4655\times 10^{3}$	$3.0165\times 10^{3}$	$2.8542\times 10^{3}$	$2.5060\times 10^{3}$	$2.5140\times 10^{3}$	$2.5830\times 10^{3}$	$2.3889\times 10^{3}$	$2.2433\times 10^{3}$
	Rank	7	6	8	9	4	2	3	5	1
f21	Mean	$2.3582\times 10^{3}$	$2.3772\times 10^{3}$	$2.3807\times 10^{3}$	$2.4293\times 10^{3}$	$2.3436\times 10^{3}$	$2.3377\times 10^{3}$	$2.3414\times 10^{3}$	$2.3792\times 10^{3}$	$2.3334\times 10^{3}$
	Std	$1.1083\times 10^{1}$	$6.4273\times 10^{1}$	$2.2399\times 10^{1}$	$2.7447\times 10^{1}$	$1.1635\times 10^{1}$	$1.0031\times 10^{1}$	$1.4954\times 10^{1}$	$2.4296\times 10^{1}$	$9.3320\times 10^{0}$
	Best	$2.3288\times 10^{3}$	$2.2226\times 10^{3}$	$2.3504\times 10^{3}$	$2.3812\times 10^{3}$	$2.3201\times 10^{3}$	$2.3209\times 10^{3}$	$2.3245\times 10^{3}$	$2.2926\times 10^{3}$	$2.3144\times 10^{3}$
	worst	$2.3787\times 10^{3}$	$2.4368\times 10^{3}$	$2.4413\times 10^{3}$	$2.4877\times 10^{3}$	$2.3679\times 10^{3}$	$2.3644\times 10^{3}$	$2.3913\times 10^{3}$	$2.4212\times 10^{3}$	$2.3564\times 10^{3}$
	Rank	5	6	8	9	4	2	3	7	1
f22	Mean	$3.1116\times 10^{3}$	$2.9423\times 10^{3}$	$4.1845\times 10^{3}$	$3.7512\times 10^{3}$	$2.8927\times 10^{3}$	$3.1656\times 10^{3}$	$2.3000\times 10^{3}$	$2.6383\times 10^{3}$	$2.3000\times 10^{3}$
	Std	$1.0923\times 10^{3}$	$1.3069\times 10^{3}$	$2.1278\times 10^{3}$	$2.2574\times 10^{3}$	$1.3662\times 10^{3}$	$1.4950\times 10^{3}$	$6.5574\times 10^{-12}$	$1.0212\times 10^{3}$	$1.3829\times 10^{-11}$
	Best	$2.3001\times 10^{3}$	$2.3000\times 10^{3}$	$2.3000\times 10^{3}$	$2.3001\times 10^{3}$	$2.3000\times 10^{3}$	$2.3000\times 10^{3}$	$2.3000\times 10^{3}$	$2.3100\times 10^{3}$	$2.3000\times 10^{3}$
	worst	$4.9813\times 10^{3}$	$5.7698\times 10^{3}$	$7.8422\times 10^{3}$	$7.5237\times 10^{3}$	$6.3416\times 10^{3}$	$6.7753\times 10^{3}$	$2.3000\times 10^{3}$	$6.5764\times 10^{3}$	$2.3000\times 10^{3}$
	Rank	6	5	9	8	4	7	2	3	1
f23	Mean	$2.7437\times 10^{3}$	$2.6453\times 10^{3}$	$2.7224\times 10^{3}$	$2.8057\times 10^{3}$	$2.7012\times 10^{3}$	$2.6943\times 10^{3}$	$2.6928\times 10^{3}$	$2.7329\times 10^{3}$	$2.6826\times 10^{3}$
	Std	$2.3626\times 10^{1}$	$1.4801\times 10^{2}$	$2.0928\times 10^{1}$	$4.1426\times 10^{1}$	$1.6061\times 10^{1}$	$1.5507\times 10^{1}$	$1.7885\times 10^{1}$	$1.4321\times 10^{1}$	$1.2114\times 10^{1}$
	Best	$2.6984\times 10^{3}$	$2.4250\times 10^{3}$	$2.6962\times 10^{3}$	$2.7252\times 10^{3}$	$2.6751\times 10^{3}$	$2.6688\times 10^{3}$	$2.6662\times 10^{3}$	$2.7035\times 10^{3}$	$2.6672\times 10^{3}$
	worst	$2.7895\times 10^{3}$	$2.8082\times 10^{3}$	$2.7892\times 10^{3}$	$2.8827\times 10^{3}$	$2.7373\times 10^{3}$	$2.7257\times 10^{3}$	$2.7352\times 10^{3}$	$2.7580\times 10^{3}$	$2.7121\times 10^{3}$
	Rank	8	1	6	9	5	4	3	7	2
f24	Mean	$2.8945\times 10^{3}$	$2.9242\times 10^{3}$	$2.8899\times 10^{3}$	$2.9587\times 10^{3}$	$2.8783\times 10^{3}$	$2.8698\times 10^{3}$	$2.8723\times 10^{3}$	$2.9160\times 10^{3}$	$2.8524\times 10^{3}$
	Std	$1.8203\times 10^{1}$	$7.4464\times 10^{1}$	$1.9416\times 10^{1}$	$3.0886\times 10^{1}$	$1.6419\times 10^{1}$	$1.5820\times 10^{1}$	$1.3311\times 10^{1}$	$1.6922\times 10^{1}$	$9.0929\times 10^{0}$
	Best	$2.8648\times 10^{3}$	$2.6421\times 10^{3}$	$2.8556\times 10^{3}$	$2.8938\times 10^{3}$	$2.8484\times 10^{3}$	$2.8380\times 10^{3}$	$2.8477\times 10^{3}$	$2.8758\times 10^{3}$	$2.8347\times 10^{3}$
	worst	$2.9475\times 10^{3}$	$2.9977\times 10^{3}$	$2.9306\times 10^{3}$	$3.0429\times 10^{3}$	$2.9128\times 10^{3}$	$2.9018\times 10^{3}$	$2.9059\times 10^{3}$	$2.9429\times 10^{3}$	$2.8703\times 10^{3}$
	Rank	6	8	5	9	4	2	3	7	1
f25	Mean	$2.8874\times 10^{3}$	$2.8878\times 10^{3}$	$2.8878\times 10^{3}$	$2.9102\times 10^{3}$	$2.9127\times 10^{3}$	$2.8890\times 10^{3}$	$2.8868\times 10^{3}$	$2.8880\times 10^{3}$	$2.8880\times 10^{3}$
	Std	$2.0346\times 10^{0}$	$2.2654\times 10^{0}$	$1.8076\times 10^{0}$	$1.6794\times 10^{1}$	$1.6043\times 10^{1}$	$6.7684\times 10^{0}$	$1.9182\times 10^{0}$	$1.2213\times 10^{0}$	$7.1630\times 10^{0}$
	Best	$2.8836\times 10^{3}$	$2.8836\times 10^{3}$	$2.8834\times 10^{3}$	$2.8887\times 10^{3}$	$2.8885\times 10^{3}$	$2.8868\times 10^{3}$	$2.8835\times 10^{3}$	$2.8869\times 10^{3}$	$2.8834\times 10^{3}$
	worst	$2.8924\times 10^{3}$	$2.8936\times 10^{3}$	$2.8921\times 10^{3}$	$2.9438\times 10^{3}$	$2.9389\times 10^{3}$	$2.9247\times 10^{3}$	$2.8884\times 10^{3}$	$2.8929\times 10^{3}$	$2.9241\times 10^{3}$
	Rank	2	3	4	8	9	7	1	5	6
f26	Mean	$4.8381\times 10^{3}$	$2.9217\times 10^{3}$	$4.3825\times 10^{3}$	$4.7867\times 10^{3}$	$4.2190\times 10^{3}$	$4.0473\times 10^{3}$	$4.0019\times 10^{3}$	$4.3525\times 10^{3}$	$3.3936\times 10^{3}$
	Std	$2.8548\times 10^{2}$	$5.1398\times 10^{2}$	$2.1686\times 10^{2}$	$1.1671\times 10^{3}$	$2.0856\times 10^{2}$	$1.0241\times 10^{2}$	$1.0756\times 10^{2}$	$3.3938\times 10^{2}$	$5.2977\times 10^{2}$
	Best	$4.2131\times 10^{3}$	$2.8000\times 10^{3}$	$4.0227\times 10^{3}$	$2.8002\times 10^{3}$	$3.8451\times 10^{3}$	$3.8691\times 10^{3}$	$3.7106\times 10^{3}$	$3.6234\times 10^{3}$	$2.8000\times 10^{3}$
	worst	$5.5615\times 10^{3}$	$5.6328\times 10^{3}$	$4.8631\times 10^{3}$	$7.0417\times 10^{3}$	$4.5909\times 10^{3}$	$4.2294\times 10^{3}$	$4.2172\times 10^{3}$	$5.0131\times 10^{3}$	$4.1277\times 10^{3}$
	Rank	9	1	7	8	5	4	3	6	2
f27	Mean	$3.2604\times 10^{3}$	$3.2339\times 10^{3}$	$3.2176\times 10^{3}$	$3.2645\times 10^{3}$	$3.2311\times 10^{3}$	$3.2233\times 10^{3}$	$3.2102\times 10^{3}$	$3.2167\times 10^{3}$	$3.2014\times 10^{3}$
	Std	$1.4476\times 10^{1}$	$1.0826\times 10^{1}$	$1.1150\times 10^{1}$	$2.7515\times 10^{1}$	$1.4438\times 10^{1}$	$1.3727\times 10^{1}$	$5.3119\times 10^{0}$	$6.4606\times 10^{0}$	$9.8784\times 10^{0}$
	Best	$3.2272\times 10^{3}$	$3.2141\times 10^{3}$	$3.1929\times 10^{3}$	$3.2233\times 10^{3}$	$3.2110\times 10^{3}$	$3.2032\times 10^{3}$	$3.2023\times 10^{3}$	$3.2023\times 10^{3}$	$3.1697\times 10^{3}$
	worst	$3.2834\times 10^{3}$	$3.2546\times 10^{3}$	$3.2393\times 10^{3}$	$3.3588\times 10^{3}$	$3.2663\times 10^{3}$	$3.2591\times 10^{3}$	$3.2231\times 10^{3}$	$3.2305\times 10^{3}$	$3.2166\times 10^{3}$
	Rank	8	7	4	9	6	5	2	3	1
f28	Mean	$3.2338\times 10^{3}$	$3.1905\times 10^{3}$	$3.2343\times 10^{3}$	$3.2426\times 10^{3}$	$3.2385\times 10^{3}$	$3.2189\times 10^{3}$	$3.1461\times 10^{3}$	$3.2317\times 10^{3}$	$3.1747\times 10^{3}$
	Std	$2.1219\times 10^{1}$	$4.7066\times 10^{1}$	$1.9581\times 10^{1}$	$2.4656\times 10^{1}$	$6.8980\times 10^{1}$	$2.8981\times 10^{1}$	$5.5657\times 10^{1}$	$1.2704\times 10^{1}$	$4.7475\times 10^{1}$
	Best	$3.1947\times 10^{3}$	$3.1000\times 10^{3}$	$3.2106\times 10^{3}$	$3.2050\times 10^{3}$	$3.1000\times 10^{3}$	$3.1617\times 10^{3}$	$3.1000\times 10^{3}$	$3.2122\times 10^{3}$	$3.1000\times 10^{3}$
	worst	$3.2730\times 10^{3}$	$3.2605\times 10^{3}$	$3.2808\times 10^{3}$	$3.2817\times 10^{3}$	$3.3647\times 10^{3}$	$3.2668\times 10^{3}$	$3.2620\times 10^{3}$	$3.2723\times 10^{3}$	$3.2498\times 10^{3}$
	Rank	6	3	7	9	8	4	1	5	2
f29	Mean	$3.7784\times 10^{3}$	$3.6295\times 10^{3}$	$3.6168\times 10^{3}$	$3.9634\times 10^{3}$	$3.7831\times 10^{3}$	$3.3944\times 10^{3}$	$3.4394\times 10^{3}$	$3.6680\times 10^{3}$	$3.4786\times 10^{3}$
	Std	$1.9176\times 10^{2}$	$7.5927\times 10^{1}$	$1.4096\times 10^{2}$	$2.2009\times 10^{2}$	$1.7698\times 10^{2}$	$9.5887\times 10^{1}$	$9.8610\times 10^{1}$	$1.0708\times 10^{2}$	$5.9457\times 10^{1}$
	Best	$3.4498\times 10^{3}$	$3.4762\times 10^{3}$	$3.4082\times 10^{3}$	$3.5607\times 10^{3}$	$3.5100\times 10^{3}$	$3.2679\times 10^{3}$	$3.3164\times 10^{3}$	$3.5029\times 10^{3}$	$3.4036\times 10^{3}$
	worst	$4.2220\times 10^{3}$	$3.7513\times 10^{3}$	$3.9511\times 10^{3}$	$4.3686\times 10^{3}$	$4.2122\times 10^{3}$	$3.6876\times 10^{3}$	$3.6779\times 10^{3}$	$3.8905\times 10^{3}$	$3.6644\times 10^{3}$
	Rank	7	5	4	9	8	1	2	6	3
f30	Mean	$1.2570\times 10^{4}$	$1.6974\times 10^{4}$	$1.2126\times 10^{4}$	$8.7501\times 10^{3}$	$8.6029\times 10^{5}$	$7.5364\times 10^{3}$	$1.0327\times 10^{4}$	$1.2397\times 10^{5}$	$8.9384\times 10^{3}$
	Std	$4.9305\times 10^{3}$	$1.1755\times 10^{4}$	$4.6105\times 10^{3}$	$1.9884\times 10^{3}$	$1.5432\times 10^{6}$	$2.2927\times 10^{3}$	$4.1831\times 10^{3}$	$7.5402\times 10^{4}$	$1.4998\times 10^{3}$
	Best	$7.1742\times 10^{3}$	$6.0652\times 10^{3}$	$5.4129\times 10^{3}$	$6.1312\times 10^{3}$	$1.4831\times 10^{4}$	$5.2840\times 10^{3}$	$5.6045\times 10^{3}$	$3.5019\times 10^{4}$	$6.9808\times 10^{3}$
	worst	$2.6964\times 10^{4}$	$4.9550\times 10^{4}$	$2.1013\times 10^{4}$	$1.3885\times 10^{4}$	$6.2901\times 10^{6}$	$1.5252\times 10^{4}$	$1.9118\times 10^{4}$	$3.8128\times 10^{5}$	$1.2719\times 10^{4}$
	Rank	6	7	5	2	9	1	4	8	3
Total Rank		172	144	191	208	158	109	87	187	49
Final Rank		6	4	8	9	5	3	2	7	1

**Table 5:** Statistical Results of the 50-Dimensional Scenario (CEC2017).

f(x)	Index	SO	ED	PGA	TTAO	HGWO	EIWOA	CFDA	MGO	IMGO
f1	Mean	$7.3900\times 10^{5}$	$2.3363\times 10^{3}$	$5.8319\times 10^{5}$	$2.2402\times 10^{6}$	$3.3723\times 10^{3}$	$7.0692\times 10^{3}$	$4.5129\times 10^{3}$	$2.5815\times 10^{6}$	$3.1687\times 10^{3}$
	Std	$9.1919\times 10^{5}$	$3.0985\times 10^{3}$	$2.7803\times 10^{5}$	$3.0257\times 10^{6}$	$4.9439\times 10^{3}$	$2.7684\times 10^{3}$	$5.4852\times 10^{3}$	$4.4520\times 10^{6}$	$4.1509\times 10^{3}$
	Best	$3.0628\times 10^{4}$	$1.0022\times 10^{2}$	$1.4824\times 10^{5}$	$2.1568\times 10^{5}$	$1.1385\times 10^{2}$	$5.8861\times 10^{2}$	$1.0117\times 10^{2}$	$4.4407\times 10^{4}$	$1.0158\times 10^{2}$
	worst	$3.7418\times 10^{6}$	$1.4821\times 10^{4}$	$1.4102\times 10^{6}$	$1.5971\times 10^{7}$	$1.8061\times 10^{4}$	$9.8785\times 10^{3}$	$2.6466\times 10^{4}$	$2.3629\times 10^{7}$	$1.5250\times 10^{4}$
	Rank	7	1	6	8	3	5	4	9	2
f3	Mean	$1.2608\times 10^{5}$	$1.6374\times 10^{5}$	$1.9364\times 10^{5}$	$8.8839\times 10^{4}$	$1.6013\times 10^{4}$	$1.0229\times 10^{5}$	$1.4870\times 10^{4}$	$2.3565\times 10^{5}$	$3.9169\times 10^{4}$
	Std	$1.4016\times 10^{4}$	$1.7367\times 10^{4}$	$2.6766\times 10^{4}$	$1.4962\times 10^{4}$	$4.3182\times 10^{3}$	$2.3746\times 10^{4}$	$7.4038\times 10^{3}$	$2.4157\times 10^{4}$	$8.1078\times 10^{3}$
	Best	$9.9920\times 10^{4}$	$1.3434\times 10^{5}$	$1.5441\times 10^{5}$	$6.0878\times 10^{4}$	$7.7719\times 10^{3}$	$5.2296\times 10^{4}$	$4.3589\times 10^{3}$	$1.8459\times 10^{5}$	$2.0746\times 10^{4}$
	worst	$1.4636\times 10^{5}$	$2.0342\times 10^{5}$	$2.6429\times 10^{5}$	$1.1889\times 10^{5}$	$2.6155\times 10^{4}$	$1.4042\times 10^{5}$	$3.9459\times 10^{4}$	$2.8506\times 10^{5}$	$5.4587\times 10^{4}$
	Rank	6	7	8	4	2	5	1	9	3
f4	Mean	$5.6728\times 10^{2}$	$4.9188\times 10^{2}$	$5.6951\times 10^{2}$	$6.1755\times 10^{2}$	$6.0560\times 10^{2}$	$4.6085\times 10^{2}$	$5.1392\times 10^{2}$	$5.8030\times 10^{2}$	$5.1098\times 10^{2}$
	Std	$3.6996\times 10^{1}$	$4.7567\times 10^{1}$	$3.9376\times 10^{1}$	$4.4937\times 10^{1}$	$7.9303\times 10^{1}$	$4.1226\times 10^{1}$	$5.2288\times 10^{1}$	$3.4919\times 10^{1}$	$4.5897\times 10^{1}$
	Best	$5.0115\times 10^{2}$	$4.0305\times 10^{2}$	$4.6320\times 10^{2}$	$5.2302\times 10^{2}$	$4.8052\times 10^{2}$	$4.2183\times 10^{2}$	$4.2851\times 10^{2}$	$5.2186\times 10^{2}$	$4.2852\times 10^{2}$
	worst	$6.4287\times 10^{2}$	$6.1939\times 10^{2}$	$6.2566\times 10^{2}$	$6.8558\times 10^{2}$	$7.4654\times 10^{2}$	$6.0274\times 10^{2}$	$6.0330\times 10^{2}$	$6.4416\times 10^{2}$	$6.1696\times 10^{2}$
	Rank	5	2	6	9	8	1	4	7	3
f5	Mean	$6.3028\times 10^{2}$	$7.6628\times 10^{2}$	$6.7453\times 10^{2}$	$8.5854\times 10^{2}$	$5.9263\times 10^{2}$	$5.7765\times 10^{2}$	$5.8441\times 10^{2}$	$7.4661\times 10^{2}$	$5.9866\times 10^{2}$
	Std	$1.5993\times 10^{1}$	$2.2686\times 10^{1}$	$4.1917\times 10^{1}$	$5.4075\times 10^{1}$	$2.1694\times 10^{1}$	$1.5214\times 10^{1}$	$1.8670\times 10^{1}$	$3.1663\times 10^{1}$	$3.1400\times 10^{1}$
	Best	$5.9977\times 10^{2}$	$7.0156\times 10^{2}$	$5.7263\times 10^{2}$	$7.5685\times 10^{2}$	$5.5970\times 10^{2}$	$5.4776\times 10^{2}$	$5.5671\times 10^{2}$	$6.5739\times 10^{2}$	$5.4943\times 10^{2}$
	worst	$6.6179\times 10^{2}$	$8.0172\times 10^{2}$	$7.7958\times 10^{2}$	$9.6355\times 10^{2}$	$6.3034\times 10^{2}$	$6.0349\times 10^{2}$	$6.4356\times 10^{2}$	$8.1083\times 10^{2}$	$7.2533\times 10^{2}$
	Rank	5	8	6	9	3	1	2	7	4
f6	Mean	$6.0478\times 10^{2}$	$6.0589\times 10^{2}$	$6.0209\times 10^{2}$	$6.4752\times 10^{2}$	$6.1624\times 10^{2}$	$6.0000\times 10^{2}$	$6.0057\times 10^{2}$	$6.0043\times 10^{2}$	$6.0001\times 10^{2}$
	Std	$2.5110\times 10^{0}$	$2.0570\times 10^{0}$	$2.3613\times 10^{0}$	$9.6149\times 10^{0}$	$3.0461\times 10^{0}$	$1.5866\times 10^{-2}$	$3.0520\times 10^{-1}$	$1.7842\times 10^{-1}$	$2.7869\times 10^{-3}$
	Best	$6.0085\times 10^{2}$	$6.0042\times 10^{2}$	$6.0024\times 10^{2}$	$6.2699\times 10^{2}$	$6.0999\times 10^{2}$	$6.0000\times 10^{2}$	$6.0010\times 10^{2}$	$6.0020\times 10^{2}$	$6.0000\times 10^{2}$
	worst	$6.1119\times 10^{2}$	$6.0879\times 10^{2}$	$6.0968\times 10^{2}$	$6.7050\times 10^{2}$	$6.2266\times 10^{2}$	$6.0009\times 10^{2}$	$6.0114\times 10^{2}$	$6.0107\times 10^{2}$	$6.0001\times 10^{2}$
	Rank	6	7	5	9	8	1	4	3	2
f7	Mean	$8.9899\times 10^{2}$	$1.0255\times 10^{3}$	$9.4571\times 10^{2}$	$1.0896\times 10^{3}$	$9.2886\times 10^{2}$	$8.5359\times 10^{2}$	$8.2185\times 10^{2}$	$1.0222\times 10^{3}$	$8.5050\times 10^{2}$
	Std	$4.3858\times 10^{1}$	$2.9124\times 10^{1}$	$5.0337\times 10^{1}$	$6.9742\times 10^{1}$	$3.7347\times 10^{1}$	$5.0710\times 10^{1}$	$1.3464\times 10^{1}$	$2.8739\times 10^{1}$	$2.3014\times 10^{1}$
	Best	$8.3976\times 10^{2}$	$9.5083\times 10^{2}$	$8.5675\times 10^{2}$	$9.8132\times 10^{2}$	$8.7649\times 10^{2}$	$8.0635\times 10^{2}$	$8.0130\times 10^{2}$	$9.5648\times 10^{2}$	$8.1112\times 10^{2}$
	worst	$1.0125\times 10^{3}$	$1.0847\times 10^{3}$	$1.0709\times 10^{3}$	$1.2285\times 10^{3}$	$1.0123\times 10^{3}$	$1.0124\times 10^{3}$	$8.5162\times 10^{2}$	$1.0744\times 10^{3}$	$8.9661\times 10^{2}$
	Rank	4	8	6	9	5	3	1	7	2
f8	Mean	$9.3055\times 10^{2}$	$1.0662\times 10^{3}$	$9.5682\times 10^{2}$	$1.1257\times 10^{3}$	$8.9389\times 10^{2}$	$8.8011\times 10^{2}$	$8.8149\times 10^{2}$	$1.0360\times 10^{3}$	$9.0168\times 10^{2}$
	Std	$1.8727\times 10^{1}$	$2.2780\times 10^{1}$	$4.4478\times 10^{1}$	$5.8903\times 10^{1}$	$1.6617\times 10^{1}$	$2.0143\times 10^{1}$	$1.6651\times 10^{1}$	$2.5197\times 10^{1}$	$2.3593\times 10^{1}$
	Best	$8.8423\times 10^{2}$	$1.0205\times 10^{3}$	$8.6766\times 10^{2}$	$1.0198\times 10^{3}$	$8.6368\times 10^{2}$	$8.3288\times 10^{2}$	$8.5472\times 10^{2}$	$9.9055\times 10^{2}$	$8.6416\times 10^{2}$
	worst	$9.6722\times 10^{2}$	$1.1143\times 10^{3}$	$1.0448\times 10^{3}$	$1.2414\times 10^{3}$	$9.3332\times 10^{2}$	$9.1554\times 10^{2}$	$9.2857\times 10^{2}$	$1.0841\times 10^{3}$	$9.5045\times 10^{2}$
	Rank	5	8	6	9	3	1	2	7	4
f9	Mean	$2.0921\times 10^{3}$	$8.5424\times 10^{3}$	$1.2890\times 10^{3}$	$1.6320\times 10^{4}$	$2.5701\times 10^{3}$	$9.0000\times 10^{2}$	$9.0153\times 10^{2}$	$2.7133\times 10^{3}$	$9.0372\times 10^{2}$
	Std	$5.1909\times 10^{2}$	$3.4987\times 10^{3}$	$3.2215\times 10^{2}$	$5.1219\times 10^{3}$	$5.9288\times 10^{2}$	$3.9579\times 10^{-10}$	$1.5349\times 10^{0}$	$7.3316\times 10^{2}$	$4.0887\times 10^{0}$
	Best	$1.3943\times 10^{3}$	$2.0131\times 10^{3}$	$9.1332\times 10^{2}$	$4.1195\times 10^{3}$	$1.6094\times 10^{3}$	$9.0000\times 10^{2}$	$9.0000\times 10^{2}$	$1.8720\times 10^{3}$	$9.0009\times 10^{2}$
	worst	$3.6017\times 10^{3}$	$1.5099\times 10^{4}$	$2.2532\times 10^{3}$	$2.5334\times 10^{4}$	$3.7008\times 10^{3}$	$9.0000\times 10^{2}$	$9.0518\times 10^{2}$	$4.8336\times 10^{3}$	$9.1619\times 10^{2}$
	Rank	5	8	4	9	6	1	2	7	3
f10	Mean	$5.4428\times 10^{3}$	$6.6786\times 10^{3}$	$7.3517\times 10^{3}$	$1.0475\times 10^{4}$	$7.1391\times 10^{3}$	$5.9945\times 10^{3}$	$6.7033\times 10^{3}$	$1.0212\times 10^{4}$	$7.3919\times 10^{3}$
	Std	$8.4537\times 10^{2}$	$4.0448\times 10^{2}$	$1.0236\times 10^{3}$	$7.2303\times 10^{2}$	$9.0383\times 10^{2}$	$1.1474\times 10^{3}$	$1.2346\times 10^{3}$	$5.5449\times 10^{2}$	$6.3964\times 10^{2}$
	Best	$4.1871\times 10^{3}$	$5.7232\times 10^{3}$	$5.4957\times 10^{3}$	$8.8001\times 10^{3}$	$5.5404\times 10^{3}$	$3.2985\times 10^{3}$	$3.8165\times 10^{3}$	$8.7750\times 10^{3}$	$6.0425\times 10^{3}$
	worst	$8.5923\times 10^{3}$	$7.2649\times 10^{3}$	$8.8945\times 10^{3}$	$1.1958\times 10^{4}$	$8.6242\times 10^{3}$	$7.9918\times 10^{3}$	$9.4848\times 10^{3}$	$1.1478\times 10^{4}$	$8.6775\times 10^{3}$
	Rank	1	3	6	9	5	2	4	8	7
f11	Mean	$1.3776\times 10^{3}$	$1.2460\times 10^{3}$	$1.2842\times 10^{3}$	$1.3332\times 10^{3}$	$1.2581\times 10^{3}$	$1.2667\times 10^{3}$	$1.1551\times 10^{3}$	$1.4551\times 10^{3}$	$1.2144\times 10^{3}$
	Std	$7.1881\times 10^{1}$	$4.5107\times 10^{1}$	$7.1237\times 10^{1}$	$5.1060\times 10^{1}$	$3.5851\times 10^{1}$	$6.5590\times 10^{1}$	$1.6851\times 10^{1}$	$6.4511\times 10^{1}$	$4.5609\times 10^{1}$
	Best	$1.2169\times 10^{3}$	$1.1579\times 10^{3}$	$1.1970\times 10^{3}$	$1.2368\times 10^{3}$	$1.1918\times 10^{3}$	$1.1662\times 10^{3}$	$1.1351\times 10^{3}$	$1.3312\times 10^{3}$	$1.1592\times 10^{3}$
	worst	$1.5347\times 10^{3}$	$1.3637\times 10^{3}$	$1.5720\times 10^{3}$	$1.4417\times 10^{3}$	$1.3749\times 10^{3}$	$1.3863\times 10^{3}$	$1.2035\times 10^{3}$	$1.6176\times 10^{3}$	$1.4046\times 10^{3}$
	Rank	8	3	6	7	4	5	1	9	2
f12	Mean	$5.8577\times 10^{6}$	$1.5788\times 10^{6}$	$1.0036\times 10^{7}$	$5.6244\times 10^{6}$	$8.1952\times 10^{5}$	$5.8915\times 10^{6}$	$9.0488\times 10^{5}$	$2.1898\times 10^{7}$	$4.0561\times 10^{5}$
	Std	$4.7580\times 10^{6}$	$1.0194\times 10^{6}$	$7.7151\times 10^{6}$	$3.1970\times 10^{6}$	$4.5584\times 10^{5}$	$1.6130\times 10^{6}$	$7.3904\times 10^{5}$	$7.3514\times 10^{6}$	$2.7203\times 10^{5}$
	Best	$7.3578\times 10^{5}$	$2.8245\times 10^{5}$	$1.0169\times 10^{6}$	$1.2803\times 10^{6}$	$2.0038\times 10^{5}$	$3.0154\times 10^{6}$	$1.4892\times 10^{5}$	$1.3571\times 10^{7}$	$5.1911\times 10^{4}$
	worst	$1.8033\times 10^{7}$	$4.0421\times 10^{6}$	$3.3411\times 10^{7}$	$1.6059\times 10^{7}$	$2.0212\times 10^{6}$	$9.9646\times 10^{6}$	$2.7034\times 10^{6}$	$3.8307\times 10^{7}$	$9.6401\times 10^{5}$
	Rank	6	4	8	5	2	7	3	9	1
f13	Mean	$1.7414\times 10^{4}$	$4.2520\times 10^{3}$	$1.0169\times 10^{4}$	$9.8425\times 10^{3}$	$8.1426\times 10^{3}$	$4.8705\times 10^{3}$	$6.1566\times 10^{3}$	$1.1774\times 10^{5}$	$4.5047\times 10^{3}$
	Std	$1.1674\times 10^{4}$	$3.1625\times 10^{3}$	$8.6732\times 10^{3}$	$5.5341\times 10^{3}$	$2.5087\times 10^{3}$	$5.1252\times 10^{3}$	$6.2050\times 10^{3}$	$1.6058\times 10^{5}$	$2.6195\times 10^{3}$
	Best	$3.7376\times 10^{3}$	$1.7578\times 10^{3}$	$1.6743\times 10^{3}$	$3.3640\times 10^{3}$	$4.5889\times 10^{3}$	$1.5396\times 10^{3}$	$1.3506\times 10^{3}$	$6.1881\times 10^{3}$	$1.4730\times 10^{3}$
	worst	$4.9726\times 10^{4}$	$1.4037\times 10^{4}$	$3.9309\times 10^{4}$	$2.8170\times 10^{4}$	$1.6124\times 10^{4}$	$2.5262\times 10^{4}$	$2.8193\times 10^{4}$	$8.4269\times 10^{5}$	$1.2774\times 10^{4}$
	Rank	8	1	7	6	5	3	4	9	2
f14	Mean	$1.1682\times 10^{5}$	$1.4268\times 10^{5}$	$3.1980\times 10^{5}$	$1.5851\times 10^{5}$	$3.8799\times 10^{4}$	$4.2588\times 10^{5}$	$5.3294\times 10^{4}$	$3.3349\times 10^{5}$	$1.7719\times 10^{3}$
	Std	$1.0561\times 10^{5}$	$1.3360\times 10^{5}$	$1.9981\times 10^{5}$	$1.1438\times 10^{5}$	$3.1940\times 10^{4}$	$1.7013\times 10^{5}$	$5.4634\times 10^{4}$	$1.5431\times 10^{5}$	$2.2193\times 10^{2}$
	Best	$6.8061\times 10^{3}$	$8.3441\times 10^{3}$	$2.0148\times 10^{4}$	$3.4154\times 10^{4}$	$3.4779\times 10^{3}$	$1.9067\times 10^{5}$	$7.0031\times 10^{3}$	$7.7518\times 10^{4}$	$1.5677\times 10^{3}$
	worst	$4.4361\times 10^{5}$	$6.2922\times 10^{5}$	$7.1559\times 10^{5}$	$4.3660\times 10^{5}$	$1.3601\times 10^{5}$	$8.2133\times 10^{5}$	$2.6546\times 10^{5}$	$6.5551\times 10^{5}$	$2.8423\times 10^{3}$
	Rank	4	5	7	6	2	9	3	8	1
f15	Mean	$1.1278\times 10^{4}$	$1.1929\times 10^{4}$	$1.2752\times 10^{4}$	$1.0491\times 10^{4}$	$8.2297\times 10^{3}$	$7.7423\times 10^{3}$	$1.2486\times 10^{4}$	$2.8770\times 10^{4}$	$7.5446\times 10^{3}$
	Std	$6.2268\times 10^{3}$	$4.5186\times 10^{3}$	$9.3561\times 10^{3}$	$5.9240\times 10^{3}$	$5.2778\times 10^{3}$	$5.9051\times 10^{3}$	$6.6086\times 10^{3}$	$1.8430\times 10^{4}$	$4.9688\times 10^{3}$
	Best	$3.0327\times 10^{3}$	$2.4674\times 10^{3}$	$1.9178\times 10^{3}$	$2.2877\times 10^{3}$	$1.9563\times 10^{3}$	$1.6251\times 10^{3}$	$2.0892\times 10^{3}$	$5.3915\times 10^{3}$	$1.7696\times 10^{3}$
	worst	$2.4468\times 10^{4}$	$1.8447\times 10^{4}$	$3.3272\times 10^{4}$	$2.0625\times 10^{4}$	$2.0194\times 10^{4}$	$1.9578\times 10^{4}$	$2.1862\times 10^{4}$	$8.8338\times 10^{4}$	$1.8744\times 10^{4}$
	Rank	5	6	8	4	3	2	7	9	1
f16	Mean	$3.0020\times 10^{3}$	$3.2780\times 10^{3}$	$3.3400\times 10^{3}$	$3.7224\times 10^{3}$	$2.8388\times 10^{3}$	$2.7687\times 10^{3}$	$3.0898\times 10^{3}$	$3.2862\times 10^{3}$	$2.4984\times 10^{3}$
	Std	$3.0240\times 10^{2}$	$1.5662\times 10^{2}$	$4.8625\times 10^{2}$	$3.1986\times 10^{2}$	$3.9948\times 10^{2}$	$2.9186\times 10^{2}$	$3.5340\times 10^{2}$	$2.4612\times 10^{2}$	$3.1055\times 10^{2}$
	Best	$2.4367\times 10^{3}$	$2.8388\times 10^{3}$	$2.5467\times 10^{3}$	$2.9627\times 10^{3}$	$2.1550\times 10^{3}$	$2.3491\times 10^{3}$	$2.4027\times 10^{3}$	$2.8642\times 10^{3}$	$1.9862\times 10^{3}$
	worst	$3.8204\times 10^{3}$	$3.5138\times 10^{3}$	$4.4301\times 10^{3}$	$4.4963\times 10^{3}$	$3.4790\times 10^{3}$	$3.3663\times 10^{3}$	$3.7536\times 10^{3}$	$3.6891\times 10^{3}$	$3.0491\times 10^{3}$
	Rank	4	6	8	9	3	2	5	7	1
f17	Mean	$2.7794\times 10^{3}$	$2.9549\times 10^{3}$	$2.7329\times 10^{3}$	$3.0107\times 10^{3}$	$2.8159\times 10^{3}$	$2.5343\times 10^{3}$	$2.4769\times 10^{3}$	$2.7969\times 10^{3}$	$2.3917\times 10^{3}$
	Std	$1.7009\times 10^{2}$	$1.6356\times 10^{2}$	$3.0213\times 10^{2}$	$2.6708\times 10^{2}$	$2.5441\times 10^{2}$	$3.5127\times 10^{2}$	$2.9734\times 10^{2}$	$1.5610\times 10^{2}$	$2.1732\times 10^{2}$
	Best	$2.3698\times 10^{3}$	$2.4385\times 10^{3}$	$2.2737\times 10^{3}$	$2.3299\times 10^{3}$	$2.2706\times 10^{3}$	$1.9063\times 10^{3}$	$1.8388\times 10^{3}$	$2.3285\times 10^{3}$	$2.0444\times 10^{3}$
	worst	$3.1998\times 10^{3}$	$3.3204\times 10^{3}$	$3.4082\times 10^{3}$	$3.5028\times 10^{3}$	$3.2946\times 10^{3}$	$3.0461\times 10^{3}$	$3.2745\times 10^{3}$	$3.0516\times 10^{3}$	$2.8993\times 10^{3}$
	Rank	5	8	4	9	7	3	2	6	1
f18	Mean	$1.3168\times 10^{6}$	$2.3274\times 10^{6}$	$3.1848\times 10^{6}$	$1.1719\times 10^{6}$	$2.8211\times 10^{5}$	$1.2211\times 10^{6}$	$1.6611\times 10^{5}$	$2.6565\times 10^{6}$	$6.6698\times 10^{4}$
	Std	$8.8596\times 10^{5}$	$1.0787\times 10^{6}$	$2.8941\times 10^{6}$	$8.1308\times 10^{5}$	$2.6390\times 10^{5}$	$8.0903\times 10^{5}$	$1.0372\times 10^{5}$	$1.4496\times 10^{6}$	$3.5335\times 10^{4}$
	Best	$1.8359\times 10^{5}$	$1.3725\times 10^{5}$	$2.8172\times 10^{5}$	$1.2300\times 10^{5}$	$4.6932\times 10^{4}$	$1.6409\times 10^{5}$	$1.6027\times 10^{4}$	$4.5529\times 10^{5}$	$2.2638\times 10^{4}$
	worst	$3.5684\times 10^{6}$	$4.1591\times 10^{6}$	$1.2978\times 10^{7}$	$3.8033\times 10^{6}$	$1.3580\times 10^{6}$	$4.4310\times 10^{6}$	$4.9012\times 10^{5}$	$6.1353\times 10^{6}$	$1.9513\times 10^{5}$
	Rank	6	7	9	4	3	5	2	8	1
f19	Mean	$1.7959\times 10^{4}$	$2.1186\times 10^{4}$	$1.1577\times 10^{4}$	$1.7294\times 10^{4}$	$1.9166\times 10^{4}$	$1.0563\times 10^{4}$	$1.7909\times 10^{4}$	$6.8366\times 10^{4}$	$6.6223\times 10^{3}$
	Std	$1.2823\times 10^{4}$	$7.7152\times 10^{3}$	$1.1440\times 10^{4}$	$8.4022\times 10^{3}$	$8.6173\times 10^{3}$	$5.5266\times 10^{3}$	$1.2032\times 10^{4}$	$7.1467\times 10^{4}$	$6.2217\times 10^{3}$
	Best	$2.0960\times 10^{3}$	$5.4456\times 10^{3}$	$2.0134\times 10^{3}$	$3.8594\times 10^{3}$	$3.4594\times 10^{3}$	$1.9720\times 10^{3}$	$1.9802\times 10^{3}$	$6.1747\times 10^{3}$	$2.0538\times 10^{3}$
	worst	$4.2647\times 10^{4}$	$4.4140\times 10^{4}$	$4.3264\times 10^{4}$	$3.6661\times 10^{4}$	$3.4732\times 10^{4}$	$2.5985\times 10^{4}$	$4.3421\times 10^{4}$	$2.6633\times 10^{5}$	$2.4403\times 10^{4}$
	Rank	6	8	3	4	7	2	5	9	1
f20	Mean	$2.7211\times 10^{3}$	$3.0119\times 10^{3}$	$2.9511\times 10^{3}$	$3.1365\times 10^{3}$	$2.8158\times 10^{3}$	$2.6067\times 10^{3}$	$2.7918\times 10^{3}$	$2.8986\times 10^{3}$	$2.5400\times 10^{3}$
	Std	$3.0337\times 10^{2}$	$1.5173\times 10^{2}$	$3.6234\times 10^{2}$	$1.5789\times 10^{2}$	$2.5481\times 10^{2}$	$2.7453\times 10^{2}$	$3.3382\times 10^{2}$	$1.3140\times 10^{2}$	$1.7482\times 10^{2}$
	Best	$2.2041\times 10^{3}$	$2.6419\times 10^{3}$	$2.3364\times 10^{3}$	$2.7869\times 10^{3}$	$2.2899\times 10^{3}$	$2.1617\times 10^{3}$	$2.1838\times 10^{3}$	$2.6136\times 10^{3}$	$2.2064\times 10^{3}$
	worst	$3.6715\times 10^{3}$	$3.2873\times 10^{3}$	$3.5734\times 10^{3}$	$3.4772\times 10^{3}$	$3.3550\times 10^{3}$	$3.1812\times 10^{3}$	$3.5929\times 10^{3}$	$3.1231\times 10^{3}$	$2.8756\times 10^{3}$
	Rank	3	8	7	9	5	2	4	6	1
f21	Mean	$2.4262\times 10^{3}$	$2.5620\times 10^{3}$	$2.4664\times 10^{3}$	$2.6301\times 10^{3}$	$2.3961\times 10^{3}$	$2.3852\times 10^{3}$	$2.3874\times 10^{3}$	$2.5453\times 10^{3}$	$2.3816\times 10^{3}$
	Std	$1.8452\times 10^{1}$	$2.7775\times 10^{1}$	$4.5233\times 10^{1}$	$7.1129\times 10^{1}$	$2.0939\times 10^{1}$	$1.8649\times 10^{1}$	$2.5823\times 10^{1}$	$2.8392\times 10^{1}$	$1.6577\times 10^{1}$
	Best	$2.3893\times 10^{3}$	$2.4972\times 10^{3}$	$2.4062\times 10^{3}$	$2.5117\times 10^{3}$	$2.3676\times 10^{3}$	$2.3514\times 10^{3}$	$2.3488\times 10^{3}$	$2.4859\times 10^{3}$	$2.3466\times 10^{3}$
	worst	$2.4678\times 10^{3}$	$2.5990\times 10^{3}$	$2.5806\times 10^{3}$	$2.8147\times 10^{3}$	$2.4518\times 10^{3}$	$2.4373\times 10^{3}$	$2.4516\times 10^{3}$	$2.5964\times 10^{3}$	$2.4167\times 10^{3}$
	Rank	5	8	6	9	4	2	3	7	1
f22	Mean	$7.4430\times 10^{3}$	$8.4954\times 10^{3}$	$9.1383\times 10^{3}$	$1.1258\times 10^{4}$	$7.9960\times 10^{3}$	$7.9859\times 10^{3}$	$2.9314\times 10^{3}$	$1.1693\times 10^{4}$	$2.9125\times 10^{3}$
	Std	$1.9034\times 10^{3}$	$1.2188\times 10^{3}$	$1.0600\times 10^{3}$	$3.5924\times 10^{3}$	$1.8476\times 10^{3}$	$9.2166\times 10^{2}$	$1.9594\times 10^{3}$	$7.2462\times 10^{2}$	$1.9120\times 10^{3}$
	Best	$4.8824\times 10^{3}$	$2.3054\times 10^{3}$	$7.3560\times 10^{3}$	$2.3109\times 10^{3}$	$2.3000\times 10^{3}$	$6.3288\times 10^{3}$	$2.3000\times 10^{3}$	$9.4644\times 10^{3}$	$2.3000\times 10^{3}$
	worst	$1.4387\times 10^{4}$	$9.2556\times 10^{3}$	$1.1372\times 10^{4}$	$1.3274\times 10^{4}$	$1.0367\times 10^{4}$	$9.4726\times 10^{3}$	$9.6916\times 10^{3}$	$1.2999\times 10^{4}$	$9.8246\times 10^{3}$
	Rank	3	6	7	8	5	4	2	9	1
f23	Mean	$2.9288\times 10^{3}$	$3.0579\times 10^{3}$	$2.9015\times 10^{3}$	$3.1282\times 10^{3}$	$2.8473\times 10^{3}$	$2.8054\times 10^{3}$	$2.8144\times 10^{3}$	$2.9718\times 10^{3}$	$2.8063\times 10^{3}$
	Std	$3.8120\times 10^{1}$	$1.0091\times 10^{2}$	$4.7294\times 10^{1}$	$6.6643\times 10^{1}$	$3.5384\times 10^{1}$	$2.6692\times 10^{1}$	$2.1999\times 10^{1}$	$1.7124\times 10^{1}$	$1.9782\times 10^{1}$
	Best	$2.8323\times 10^{3}$	$2.7123\times 10^{3}$	$2.8228\times 10^{3}$	$2.9873\times 10^{3}$	$2.7840\times 10^{3}$	$2.7634\times 10^{3}$	$2.7728\times 10^{3}$	$2.9370\times 10^{3}$	$2.7684\times 10^{3}$
	worst	$2.9927\times 10^{3}$	$3.1520\times 10^{3}$	$3.0036\times 10^{3}$	$3.2697\times 10^{3}$	$2.9299\times 10^{3}$	$2.8782\times 10^{3}$	$2.8469\times 10^{3}$	$2.9985\times 10^{3}$	$2.8457\times 10^{3}$
	Rank	6	8	5	9	4	1	3	7	2
f24	Mean	$3.0674\times 10^{3}$	$3.2038\times 10^{3}$	$3.0485\times 10^{3}$	$3.3165\times 10^{3}$	$3.0244\times 10^{3}$	$2.9954\times 10^{3}$	$2.9932\times 10^{3}$	$3.1559\times 10^{3}$	$2.9689\times 10^{3}$
	Std	$3.5395\times 10^{1}$	$6.5500\times 10^{1}$	$3.4708\times 10^{1}$	$5.9760\times 10^{1}$	$4.9807\times 10^{1}$	$1.5981\times 10^{1}$	$2.1016\times 10^{1}$	$2.6502\times 10^{1}$	$1.6658\times 10^{1}$
	Best	$3.0128\times 10^{3}$	$3.0972\times 10^{3}$	$2.9799\times 10^{3}$	$3.2085\times 10^{3}$	$2.9559\times 10^{3}$	$2.9689\times 10^{3}$	$2.9557\times 10^{3}$	$3.1014\times 10^{3}$	$2.9452\times 10^{3}$
	worst	$3.1521\times 10^{3}$	$3.3140\times 10^{3}$	$3.1082\times 10^{3}$	$3.4864\times 10^{3}$	$3.1274\times 10^{3}$	$3.0240\times 10^{3}$	$3.0235\times 10^{3}$	$3.2074\times 10^{3}$	$3.0067\times 10^{3}$
	Rank	6	8	5	9	4	3	2	7	1
f25	Mean	$3.0572\times 10^{3}$	$3.0477\times 10^{3}$	$3.0567\times 10^{3}$	$3.1435\times 10^{3}$	$3.1879\times 10^{3}$	$3.0563\times 10^{3}$	$3.0557\times 10^{3}$	$3.0628\times 10^{3}$	$3.0574\times 10^{3}$
	Std	$2.7368\times 10^{1}$	$3.6079\times 10^{1}$	$2.9101\times 10^{1}$	$3.1306\times 10^{1}$	$8.4949\times 10^{1}$	$2.2705\times 10^{1}$	$3.0540\times 10^{1}$	$2.2620\times 10^{1}$	$3.3361\times 10^{1}$
	Best	$2.9917\times 10^{3}$	$2.9613\times 10^{3}$	$2.9936\times 10^{3}$	$3.0663\times 10^{3}$	$3.0914\times 10^{3}$	$3.0175\times 10^{3}$	$2.9824\times 10^{3}$	$3.0303\times 10^{3}$	$2.9617\times 10^{3}$
	worst	$3.1002\times 10^{3}$	$3.1088\times 10^{3}$	$3.1138\times 10^{3}$	$3.2054\times 10^{3}$	$3.4325\times 10^{3}$	$3.0849\times 10^{3}$	$3.1068\times 10^{3}$	$3.1139\times 10^{3}$	$3.1129\times 10^{3}$
	Rank	5	1	4	8	9	3	2	7	6
f26	Mean	$5.9586\times 10^{3}$	$4.6965\times 10^{3}$	$5.4346\times 10^{3}$	$8.6818\times 10^{3}$	$5.1758\times 10^{3}$	$4.5236\times 10^{3}$	$4.6064\times 10^{3}$	$6.1325\times 10^{3}$	$4.0951\times 10^{3}$
	Std	$3.7870\times 10^{2}$	$2.1076\times 10^{3}$	$3.7373\times 10^{2}$	$2.3816\times 10^{3}$	$2.8690\times 10^{2}$	$2.1804\times 10^{2}$	$2.0134\times 10^{2}$	$3.0192\times 10^{2}$	$8.2031\times 10^{2}$
	Best	$5.2400\times 10^{3}$	$2.9000\times 10^{3}$	$4.8906\times 10^{3}$	$2.9196\times 10^{3}$	$4.7017\times 10^{3}$	$4.1290\times 10^{3}$	$4.0992\times 10^{3}$	$5.5953\times 10^{3}$	$2.9000\times 10^{3}$
	worst	$6.8744\times 10^{3}$	$7.9068\times 10^{3}$	$6.2031\times 10^{3}$	$1.1678\times 10^{4}$	$6.0396\times 10^{3}$	$4.8818\times 10^{3}$	$5.1003\times 10^{3}$	$6.5820\times 10^{3}$	$5.0605\times 10^{3}$
	Rank	7	4	6	9	5	2	3	8	1
f27	Mean	$3.5828\times 10^{3}$	$3.5161\times 10^{3}$	$3.3580\times 10^{3}$	$3.6819\times 10^{3}$	$3.4838\times 10^{3}$	$3.4355\times 10^{3}$	$3.3105\times 10^{3}$	$3.4212\times 10^{3}$	$3.2812\times 10^{3}$
	Std	$7.7505\times 10^{1}$	$9.2434\times 10^{1}$	$5.2873\times 10^{1}$	$1.3388\times 10^{2}$	$5.7194\times 10^{1}$	$6.7949\times 10^{1}$	$3.8605\times 10^{1}$	$3.7182\times 10^{1}$	$5.3993\times 10^{1}$
	Best	$3.4281\times 10^{3}$	$3.3587\times 10^{3}$	$3.2640\times 10^{3}$	$3.4430\times 10^{3}$	$3.3490\times 10^{3}$	$3.3070\times 10^{3}$	$3.2498\times 10^{3}$	$3.3441\times 10^{3}$	$3.2195\times 10^{3}$
	worst	$3.7254\times 10^{3}$	$3.6628\times 10^{3}$	$3.4518\times 10^{3}$	$3.9265\times 10^{3}$	$3.5857\times 10^{3}$	$3.5668\times 10^{3}$	$3.4006\times 10^{3}$	$3.5032\times 10^{3}$	$3.4439\times 10^{3}$
	Rank	8	7	3	9	6	5	2	4	1
f28	Mean	$3.3435\times 10^{3}$	$3.3143\times 10^{3}$	$3.3446\times 10^{3}$	$3.4153\times 10^{3}$	$3.4186\times 10^{3}$	$3.2932\times 10^{3}$	$3.2998\times 10^{3}$	$3.3494\times 10^{3}$	$3.3113\times 10^{3}$
	Std	$2.5046\times 10^{1}$	$2.5544\times 10^{1}$	$2.4383\times 10^{1}$	$3.7895\times 10^{1}$	$7.3837\times 10^{1}$	$1.6948\times 10^{1}$	$1.7563\times 10^{1}$	$5.7451\times 10^{1}$	$4.0834\times 10^{1}$
	Best	$3.3095\times 10^{3}$	$3.2591\times 10^{3}$	$3.2981\times 10^{3}$	$3.3417\times 10^{3}$	$3.2612\times 10^{3}$	$3.2603\times 10^{3}$	$3.2589\times 10^{3}$	$3.2915\times 10^{3}$	$3.2594\times 10^{3}$
	worst	$3.4118\times 10^{3}$	$3.3651\times 10^{3}$	$3.4108\times 10^{3}$	$3.4937\times 10^{3}$	$3.6116\times 10^{3}$	$3.3229\times 10^{3}$	$3.3195\times 10^{3}$	$3.6077\times 10^{3}$	$3.4021\times 10^{3}$
	Rank	5	4	6	8	9	1	2	7	3
f29	Mean	$4.4027\times 10^{3}$	$4.3774\times 10^{3}$	$4.1388\times 10^{3}$	$4.7817\times 10^{3}$	$4.5204\times 10^{3}$	$3.6222\times 10^{3}$	$3.5269\times 10^{3}$	$4.4000\times 10^{3}$	$3.6724\times 10^{3}$
	Std	$2.8774\times 10^{2}$	$1.8934\times 10^{2}$	$3.4928\times 10^{2}$	$3.9454\times 10^{2}$	$4.0330\times 10^{2}$	$1.9440\times 10^{2}$	$2.1783\times 10^{2}$	$1.9667\times 10^{2}$	$1.4223\times 10^{2}$
	Best	$3.9134\times 10^{3}$	$3.9283\times 10^{3}$	$3.4334\times 10^{3}$	$4.1515\times 10^{3}$	$3.5015\times 10^{3}$	$3.3032\times 10^{3}$	$3.2274\times 10^{3}$	$4.0561\times 10^{3}$	$3.4293\times 10^{3}$
	worst	$5.0512\times 10^{3}$	$4.7494\times 10^{3}$	$4.9045\times 10^{3}$	$5.7578\times 10^{3}$	$5.1738\times 10^{3}$	$3.9844\times 10^{3}$	$4.0946\times 10^{3}$	$4.7760\times 10^{3}$	$4.0139\times 10^{3}$
	Rank	7	5	4	9	8	2	1	6	3
f30	Mean	$1.4976\times 10^{6}$	$1.0637\times 10^{6}$	$1.1342\times 10^{6}$	$1.0209\times 10^{6}$	$6.0115\times 10^{7}$	$1.2234\times 10^{6}$	$9.7951\times 10^{5}$	$7.4119\times 10^{6}$	$1.0353\times 10^{6}$
	Std	$5.6672\times 10^{5}$	$2.3848\times 10^{5}$	$3.2419\times 10^{5}$	$1.6670\times 10^{5}$	$1.8343\times 10^{7}$	$3.6134\times 10^{5}$	$1.9985\times 10^{5}$	$2.5736\times 10^{6}$	$2.0767\times 10^{5}$
	Best	$7.5737\times 10^{5}$	$7.0549\times 10^{5}$	$6.1958\times 10^{5}$	$8.0438\times 10^{5}$	$3.3613\times 10^{7}$	$8.6755\times 10^{5}$	$6.7309\times 10^{5}$	$3.0309\times 10^{6}$	$7.2459\times 10^{5}$
	worst	$3.0938\times 10^{6}$	$1.7750\times 10^{6}$	$1.7268\times 10^{6}$	$1.5958\times 10^{6}$	$1.0278\times 10^{8}$	$2.2910\times 10^{6}$	$1.4280\times 10^{6}$	$1.5819\times 10^{7}$	$1.5289\times 10^{6}$
	Rank	7	4	5	2	9	6	1	8	3
Total Rank		158	163	171	218	147	89	81	214	64
Final Rank		5	6	7	9	4	3	2	8	1

**Table 6:** Statistical Results of the 20-Dimensional Scenario (CEC2022).

f(x)	Index	IMGO	SO	ED	PGA	TTAO	HGWO	EIWOA	CFDA	MGO
f1	Mean	$3.0011\times 10^{2}$	$1.1404\times 10^{3}$	$4.2070\times 10^{3}$	$4.4007\times 10^{3}$	$3.1327\times 10^{2}$	$3.0000\times 10^{2}$	$1.3271\times 10^{3}$	$3.0000\times 10^{2}$	$6.8528\times 10^{3}$
	Std	$2.8254\times 10^{-1}$	$5.7782\times 10^{2}$	$1.6839\times 10^{3}$	$1.9472\times 10^{3}$	$1.0786\times 10^{1}$	$3.8281\times 10^{-6}$	$5.0637\times 10^{2}$	$2.1277\times 10^{-10}$	$2.4310\times 10^{3}$
	Best	$3.0000\times 10^{2}$	$4.5364\times 10^{2}$	$1.8438\times 10^{3}$	$1.2708\times 10^{3}$	$3.0061\times 10^{2}$	$3.0000\times 10^{2}$	$6.7308\times 10^{2}$	$3.0000\times 10^{2}$	$3.3610\times 10^{3}$
	worst	$3.0145\times 10^{2}$	$2.4395\times 10^{3}$	$8.6729\times 10^{3}$	$9.9134\times 10^{3}$	$3.4432\times 10^{2}$	$3.0000\times 10^{2}$	$2.6964\times 10^{3}$	$3.0000\times 10^{2}$	$1.2253\times 10^{4}$
	Rank	3	5	7	8	4	2	6	1	9
f2	Mean	$4.4457\times 10^{2}$	$4.4382\times 10^{2}$	$4.4245\times 10^{2}$	$4.5040\times 10^{2}$	$4.5908\times 10^{2}$	$4.5009\times 10^{2}$	$4.5142\times 10^{2}$	$4.4561\times 10^{2}$	$4.4399\times 10^{2}$
	Std	$1.5494\times 10^{1}$	$1.3878\times 10^{1}$	$1.3628\times 10^{1}$	$6.2258\times 10^{0}$	$1.3193\times 10^{1}$	$1.9871\times 10^{1}$	$7.0977\times 10^{0}$	$1.2151\times 10^{1}$	$6.6398\times 10^{0}$
	Best	$4.0000\times 10^{2}$	$4.0713\times 10^{2}$	$4.0707\times 10^{2}$	$4.4490\times 10^{2}$	$4.2509\times 10^{2}$	$4.0001\times 10^{2}$	$4.4908\times 10^{2}$	$4.0006\times 10^{2}$	$4.2564\times 10^{2}$
	worst	$4.6864\times 10^{2}$	$4.6943\times 10^{2}$	$4.4908\times 10^{2}$	$4.7322\times 10^{2}$	$4.7596\times 10^{2}$	$5.2557\times 10^{2}$	$4.7306\times 10^{2}$	$4.4908\times 10^{2}$	$4.4923\times 10^{2}$
	Rank	4	2	1	7	9	6	8	5	3
f3	Mean	$6.0000\times 10^{2}$	$6.0022\times 10^{2}$	$6.0000\times 10^{2}$	$6.0000\times 10^{2}$	$6.1031\times 10^{2}$	$6.0291\times 10^{2}$	$6.0000\times 10^{2}$	$6.0001\times 10^{2}$	$6.0000\times 10^{2}$
	Std	$7.6551\times 10^{-6}$	$2.8429\times 10^{-1}$	$3.3849\times 10^{-3}$	$1.2130\times 10^{-2}$	$7.0635\times 10^{0}$	$1.5662\times 10^{0}$	$0.0000\times 10^{0}$	$1.0849\times 10^{-2}$	$2.9399\times 10^{-5}$
	Best	$6.0000\times 10^{2}$	$6.0003\times 10^{2}$	$6.0000\times 10^{2}$	$6.0000\times 10^{2}$	$6.0138\times 10^{2}$	$6.0050\times 10^{2}$	$6.0000\times 10^{2}$	$6.0000\times 10^{2}$	$6.0000\times 10^{2}$
	worst	$6.0000\times 10^{2}$	$6.0115\times 10^{2}$	$6.0001\times 10^{2}$	$6.0006\times 10^{2}$	$6.3295\times 10^{2}$	$6.0789\times 10^{2}$	$6.0000\times 10^{2}$	$6.0004\times 10^{2}$	$6.0000\times 10^{2}$
	Rank	2	7	4	5	9	8	1	6	3
f4	Mean	$8.1977\times 10^{2}$	$8.2348\times 10^{2}$	$8.6058\times 10^{2}$	$8.4379\times 10^{2}$	$8.6600\times 10^{2}$	$8.2315\times 10^{2}$	$8.2499\times 10^{2}$	$8.2777\times 10^{2}$	$8.4098\times 10^{2}$
	Std	$6.1430\times 10^{0}$	$5.7755\times 10^{0}$	$1.2923\times 10^{1}$	$1.6134\times 10^{1}$	$1.0838\times 10^{1}$	$1.0068\times 10^{1}$	$7.8881\times 10^{0}$	$9.3559\times 10^{0}$	$1.0965\times 10^{1}$
	Best	$8.0895\times 10^{2}$	$8.1393\times 10^{2}$	$8.3872\times 10^{2}$	$8.1293\times 10^{2}$	$8.4678\times 10^{2}$	$8.0995\times 10^{2}$	$8.1293\times 10^{2}$	$8.1393\times 10^{2}$	$8.1730\times 10^{2}$
	worst	$8.3681\times 10^{2}$	$8.3582\times 10^{2}$	$8.8881\times 10^{2}$	$8.7636\times 10^{2}$	$8.9035\times 10^{2}$	$8.4975\times 10^{2}$	$8.4488\times 10^{2}$	$8.5772\times 10^{2}$	$8.7083\times 10^{2}$
	Rank	1	3	8	7	9	2	4	5	6
f5	Mean	$9.0000\times 10^{2}$	$9.5765\times 10^{2}$	$9.1115\times 10^{2}$	$9.0070\times 10^{2}$	$9.4798\times 10^{2}$	$9.5613\times 10^{2}$	$9.0000\times 10^{2}$	$9.0000\times 10^{2}$	$9.0681\times 10^{2}$
	Std	$1.6346\times 10^{-2}$	$5.8902\times 10^{1}$	$1.7289\times 10^{1}$	$1.5113\times 10^{0}$	$6.9606\times 10^{1}$	$3.2591\times 10^{1}$	$2.4932\times 10^{-7}$	$1.6346\times 10^{-2}$	$6.0190\times 10^{0}$
	Best	$9.0000\times 10^{2}$	$9.0000\times 10^{2}$	$9.0018\times 10^{2}$	$9.0000\times 10^{2}$	$9.0729\times 10^{2}$	$9.1390\times 10^{2}$	$9.0000\times 10^{2}$	$9.0000\times 10^{2}$	$9.0078\times 10^{2}$
	worst	$9.0009\times 10^{2}$	$1.0665\times 10^{3}$	$9.7818\times 10^{2}$	$9.0798\times 10^{2}$	$1.2072\times 10^{3}$	$1.0322\times 10^{3}$	$9.0000\times 10^{2}$	$9.0009\times 10^{2}$	$9.2449\times 10^{2}$
	Rank	2	9	6	4	7	8	1	3	5
f6	Mean	$2.8280\times 10^{3}$	$6.1400\times 10^{3}$	$6.9571\times 10^{3}$	$1.0186\times 10^{4}$	$4.7808\times 10^{3}$	$4.5926\times 10^{3}$	$5.0272\times 10^{3}$	$6.9122\times 10^{3}$	$3.2896\times 10^{4}$
	Std	$1.4754\times 10^{3}$	$5.4293\times 10^{3}$	$2.0381\times 10^{3}$	$8.1894\times 10^{3}$	$2.6876\times 10^{3}$	$3.4611\times 10^{3}$	$3.8853\times 10^{3}$	$4.8248\times 10^{3}$	$2.1494\times 10^{4}$
	Best	$1.8773\times 10^{3}$	$1.9229\times 10^{3}$	$2.8928\times 10^{3}$	$2.0035\times 10^{3}$	$1.8428\times 10^{3}$	$1.8415\times 10^{3}$	$1.8273\times 10^{3}$	$1.9334\times 10^{3}$	$8.0010\times 10^{3}$
	worst	$6.7033\times 10^{3}$	$2.0408\times 10^{4}$	$1.1797\times 10^{4}$	$2.5073\times 10^{4}$	$1.1848\times 10^{4}$	$1.3903\times 10^{4}$	$1.6694\times 10^{4}$	$1.7349\times 10^{4}$	$1.3263\times 10^{5}$
	Rank	1	5	7	8	3	2	4	6	9
f7	Mean	$2.0259\times 10^{3}$	$2.0484\times 10^{3}$	$2.0312\times 10^{3}$	$2.0527\times 10^{3}$	$2.0823\times 10^{3}$	$2.0454\times 10^{3}$	$2.0464\times 10^{3}$	$2.0439\times 10^{3}$	$2.0363\times 10^{3}$
	Std	$3.0801\times 10^{0}$	$1.4238\times 10^{1}$	$5.5792\times 10^{0}$	$2.4910\times 10^{1}$	$2.5152\times 10^{1}$	$1.0176\times 10^{1}$	$1.5285\times 10^{1}$	$5.2982\times 10^{0}$	$5.6574\times 10^{0}$
	Best	$2.0222\times 10^{3}$	$2.0260\times 10^{3}$	$2.0170\times 10^{3}$	$2.0245\times 10^{3}$	$2.0415\times 10^{3}$	$2.0250\times 10^{3}$	$2.0103\times 10^{3}$	$2.0373\times 10^{3}$	$2.0273\times 10^{3}$
	worst	$2.0325\times 10^{3}$	$2.0825\times 10^{3}$	$2.0439\times 10^{3}$	$2.1110\times 10^{3}$	$2.1303\times 10^{3}$	$2.0644\times 10^{3}$	$2.0852\times 10^{3}$	$2.0513\times 10^{3}$	$2.0509\times 10^{3}$
	Rank	1	7	2	8	9	5	6	4	3
f8	Mean	$2.2223\times 10^{3}$	$2.2241\times 10^{3}$	$2.2232\times 10^{3}$	$2.2224\times 10^{3}$	$2.2411\times 10^{3}$	$2.2218\times 10^{3}$	$2.2280\times 10^{3}$	$2.2223\times 10^{3}$	$2.2279\times 10^{3}$
	Std	$2.8942\times 10^{0}$	$3.3475\times 10^{0}$	$5.7151\times 10^{-1}$	$3.3585\times 10^{0}$	$2.5472\times 10^{1}$	$8.3672\times 10^{-1}$	$2.2287\times 10^{1}$	$7.7442\times 10^{-1}$	$1.3300\times 10^{0}$
	Best	$2.2082\times 10^{3}$	$2.2210\times 10^{3}$	$2.2221\times 10^{3}$	$2.2206\times 10^{3}$	$2.2276\times 10^{3}$	$2.2207\times 10^{3}$	$2.2208\times 10^{3}$	$2.2210\times 10^{3}$	$2.2250\times 10^{3}$
	worst	$2.2262\times 10^{3}$	$2.2376\times 10^{3}$	$2.2245\times 10^{3}$	$2.2393\times 10^{3}$	$2.3551\times 10^{3}$	$2.2235\times 10^{3}$	$2.3454\times 10^{3}$	$2.2237\times 10^{3}$	$2.2310\times 10^{3}$
	Rank	2	6	5	4	9	1	8	3	7
f9	Mean	$2.4808\times 10^{3}$	$2.4808\times 10^{3}$	$2.4794\times 10^{3}$	$2.4808\times 10^{3}$	$2.4808\times 10^{3}$	$2.4818\times 10^{3}$	$2.4849\times 10^{3}$	$2.4808\times 10^{3}$	$2.4808\times 10^{3}$
	Std	$4.7769\times 10^{-13}$	$2.2249\times 10^{-4}$	$7.4177\times 10^{0}$	$6.2248\times 10^{-4}$	$4.4032\times 10^{-5}$	$6.9898\times 10^{-1}$	$2.0218\times 10^{0}$	$2.6995\times 10^{-5}$	$3.2913\times 10^{-3}$
	Best	$2.4808\times 10^{3}$	$2.4808\times 10^{3}$	$2.4402\times 10^{3}$	$2.4808\times 10^{3}$	$2.4808\times 10^{3}$	$2.4810\times 10^{3}$	$2.4813\times 10^{3}$	$2.4808\times 10^{3}$	$2.4808\times 10^{3}$
	worst	$2.4808\times 10^{3}$	$2.4808\times 10^{3}$	$2.4808\times 10^{3}$	$2.4808\times 10^{3}$	$2.4808\times 10^{3}$	$2.4841\times 10^{3}$	$2.4900\times 10^{3}$	$2.4808\times 10^{3}$	$2.4808\times 10^{3}$
	Rank	2	5	1	6	4	8	9	3	7
f10	Mean	$2.5086\times 10^{3}$	$2.6957\times 10^{3}$	$2.4440\times 10^{3}$	$2.5414\times 10^{3}$	$2.9613\times 10^{3}$	$2.6595\times 10^{3}$	$2.6353\times 10^{3}$	$2.5110\times 10^{3}$	$2.5105\times 10^{3}$
	Std	$3.1273\times 10^{1}$	$1.6645\times 10^{2}$	$3.4961\times 10^{1}$	$1.5069\times 10^{2}$	$7.5631\times 10^{2}$	$2.4797\times 10^{2}$	$1.4141\times 10^{2}$	$3.8591\times 10^{1}$	$3.8131\times 10^{1}$
	Best	$2.5003\times 10^{3}$	$2.4205\times 10^{3}$	$2.4114\times 10^{3}$	$2.5004\times 10^{3}$	$2.5004\times 10^{3}$	$2.5005\times 10^{3}$	$2.5004\times 10^{3}$	$2.4493\times 10^{3}$	$2.5004\times 10^{3}$
	worst	$2.6248\times 10^{3}$	$3.0305\times 10^{3}$	$2.5364\times 10^{3}$	$3.3075\times 10^{3}$	$4.7190\times 10^{3}$	$3.3411\times 10^{3}$	$2.9092\times 10^{3}$	$2.6260\times 10^{3}$	$2.6663\times 10^{3}$
	Rank	2	8	1	5	9	7	6	4	3
f11	Mean	$2.9200\times 10^{3}$	$2.8900\times 10^{3}$	$2.7017\times 10^{3}$	$2.9238\times 10^{3}$	$2.8900\times 10^{3}$	$2.9000\times 10^{3}$	$2.9254\times 10^{3}$	$2.9033\times 10^{3}$	$2.9721\times 10^{3}$
	Std	$7.6112\times 10^{1}$	$5.4739\times 10^{1}$	$9.6089\times 10^{1}$	$5.1950\times 10^{1}$	$1.0864\times 10^{2}$	$5.3859\times 10^{-10}$	$8.7649\times 10^{1}$	$1.8257\times 10^{1}$	$1.2522\times 10^{2}$
	Best	$2.6000\times 10^{3}$	$2.6002\times 10^{3}$	$2.6024\times 10^{3}$	$2.9000\times 10^{3}$	$2.6000\times 10^{3}$	$2.9000\times 10^{3}$	$2.9000\times 10^{3}$	$2.9000\times 10^{3}$	$2.7010\times 10^{3}$
	worst	$3.0000\times 10^{3}$	$2.9004\times 10^{3}$	$2.9000\times 10^{3}$	$3.1126\times 10^{3}$	$3.0000\times 10^{3}$	$2.9000\times 10^{3}$	$3.3605\times 10^{3}$	$3.0000\times 10^{3}$	$3.2681\times 10^{3}$
	Rank	6	3	1	7	2	4	8	5	9
f12	Mean	$2.9370\times 10^{3}$	$2.9803\times 10^{3}$	$2.9536\times 10^{3}$	$2.9460\times 10^{3}$	$2.9634\times 10^{3}$	$2.9519\times 10^{3}$	$2.9546\times 10^{3}$	$2.9445\times 10^{3}$	$2.9414\times 10^{3}$
	Std	$3.6808\times 10^{0}$	$1.8340\times 10^{1}$	$5.8421\times 10^{0}$	$4.6895\times 10^{0}$	$1.6641\times 10^{1}$	$8.1115\times 10^{0}$	$1.2930\times 10^{1}$	$5.9687\times 10^{0}$	$3.5916\times 10^{0}$
	Best	$2.9313\times 10^{3}$	$2.9468\times 10^{3}$	$2.9400\times 10^{3}$	$2.9399\times 10^{3}$	$2.9434\times 10^{3}$	$2.9394\times 10^{3}$	$2.9396\times 10^{3}$	$2.9357\times 10^{3}$	$2.9353\times 10^{3}$
	worst	$2.9431\times 10^{3}$	$3.0317\times 10^{3}$	$2.9670\times 10^{3}$	$2.9607\times 10^{3}$	$3.0062\times 10^{3}$	$2.9727\times 10^{3}$	$2.9940\times 10^{3}$	$2.9581\times 10^{3}$	$2.9512\times 10^{3}$
	Rank	1	9	6	4	8	5	7	3	2
Total Rank		27	69	49	73	82	58	68	48	66
Final Rank		1	7	3	8	9	4	6	2	5

**Table 7:** Friedman’s test.

Algorithm	Ranking in Dimension								
	D = 30 (CEC2017)			D = 50 (CEC2017)			D = 20 (CEC2022)		
	Mean	Std	Best	Mean	Std	Best	Mean	Std	Best
SO	5.9310	5.8966	5.8621	5.4483	5.3793	5.8966	6.0909	5.9091	5.2727
ED	4.9655	5.0690	5.1552	5.6207	4.7586	5.3448	4.3636	4.8182	5.1818
PGA	6.5862	6.4483	4.8966	5.8966	6.5517	5.3103	6.0000	6.0909	5.2727
TTAO	7.1724	6.4828	7.2759	7.5172	6.5517	7.6552	6.6364	7.0000	6.8182
HGWO	5.4483	5.5172	5.5517	5.0690	5.0000	4.8621	4.7273	5.0909	5.4545
EIWOA	3.7586	4.3103	3.5690	3.0690	4.0345	3.4828	5.4545	5.0909	4.0000
CFDA	3.0000	3.8966	3.1379	2.7931	4.2414	2.6724	3.8636	3.2273	4.5000
MGO	6.4483	4.8966	7.1724	7.3793	5.0345	7.6897	5.7273	5.4545	6.3636
IMGO	1.6897	2.4828	2.3793	2.2069	3.4483	2.5862	2.1364	2.3182	2.1364
*p*-value	$6.4278\times 10^{-19}$	$1.7895\times 10^{-8}$	$3.9145\times 10^{-16}$	$3.2401\times 10^{-21}$	$3.2324\times 10^{-5}$	$1.5558\times 10^{-22}$	0.0035	0.0020	0.0048

**Table 8:** Results of WRST (mean).

IMGOvs	Dimension											
	30 (CEC2017)				50 (CEC2017)				20 (CEC2022)			
	*p* Value	R−	R+	+/=/−	*p* Value	R−	R+	+/=/−	*p* Value	R−	R+	+/=/−
SO	$2.77\times 10^{-3}$	54.69	410.31	27/1/1	$3.10\times 10^{-2}$	80.66	384.34	27/1/1	$9.33\times 10^{-2}$	100.67	359.42	9/2/1
ED	$4.91\times 10^{-2}$	111.45	353.55	22/4/3	$1.11\times 10^{-1}$	122.72	342.28	21/7/1	$5.90\times 10^{-2}$	141.58	312.83	8/1/3
PGA	$1.52\times 10^{-3}$	64.38	400.62	27/0/2	$7.24\times 10^{-2}$	99.17	365.83	23/3/3	$1.72\times 10^{-2}$	97.75	367.25	10/1/1
TTAO	$2.53\times 10^{-2}$	32.07	432.93	28/1/0	$3.30\times 10^{-2}$	33.45	431.55	27/2/0	$7.73\times 10^{-2}$	49.58	415.42	11/1/0
HGWO	$9.43\times 10^{-3}$	92.07	372.93	24/2/3	$7.97\times 10^{-2}$	141.59	323.41	22/5/2	$8.49\times 10^{-2}$	146.25	318.75	7/2/3
EIWOA	$6.65\times 10^{-2}$	208.21	256.59	16/7/6	$1.54\times 10^{-1}$	270.97	194.03	11/9/9	$7.72\times 10^{-2}$	143.08	297.92	8/1/3
CFDA	$1.62\times 10^{-1}$	228.31	235.83	13/12/4	$1.32\times 10^{-1}$	294.31	170.69	11/9/9	$1.51\times 10^{-1}$	190.75	260.83	6/4/2
MGO	$5.42\times 10^{-5}$	19.00	446.00	29/0/0	$1.57\times 10^{-2}$	37.34	427.66	28/1/0	$6.13\times 10^{-3}$	96.42	368.58	11/0/1

**Table 9:** Model Information.

NO	Engineering Problem	D	g	h	Reference
F101	Cantilever Beam	5	6	0	[33]
F102	Step-cone pulley	5	8	3	[34]
F103	Blending-pooling-separation	9	10	1	[35]
F104	Planetary gear train design optimization	38	0	32	[36]
F105	SOPWM for 3-level inverters	25	24	1	[37]
F106	Photovoltaic parameter extraction	5	0	0	[39]

**Table 10:** The results of all algorithms on engineering cases.

Datasets		IMGO	SO	ED	PGA	TTAO	HGWO	EIWOA	CFDA	MGO
F101	Mean	$1.3400\times 10^{0}$	$1.3400\times 10^{0}$	$1.3400\times 10^{0}$	$1.3400\times 10^{0}$	$1.3400\times 10^{0}$	$1.3400\times 10^{0}$	$1.3400\times 10^{0}$	$1.3400\times 10^{0}$	$1.3400\times 10^{0}$
	Std	$1.8011\times 10^{-10}$	$1.0861\times 10^{-6}$	$1.1013\times 10^{-6}$	$3.5844\times 10^{-5}$	$6.0377\times 10^{-6}$	$1.0570\times 10^{-5}$	$3.0521\times 10^{-7}$	$2.3393\times 10^{-6}$	$2.3833\times 10^{-6}$
	Best	$1.3400\times 10^{0}$	$1.3400\times 10^{0}$	$1.3400\times 10^{0}$	$1.3400\times 10^{0}$	$1.3400\times 10^{0}$	$1.3400\times 10^{0}$	$1.3400\times 10^{0}$	$1.3400\times 10^{0}$	$1.3400\times 10^{0}$
	Rank	1	3	4	9	7	8	2	6	5
F102	Mean	$1.6086\times 10^{1}$	$1.6096\times 10^{1}$	$1.6127\times 10^{1}$	$1.6984\times 10^{1}$	$1.6584\times 10^{1}$	$1.6881\times 10^{1}$	$1.6658\times 10^{1}$	$1.6086\times 10^{1}$	$1.6316\times 10^{1}$
	Std	$9.0216\times 10^{-15}$	$2.3143\times 10^{-2}$	$2.2767\times 10^{-2}$	$1.2077\times 10^{-1}$	$1.5023\times 10^{-1}$	$2.4074\times 10^{-1}$	$2.7091\times 10^{-1}$	$5.8922\times 10^{-14}$	$2.8653\times 10^{-1}$
	Best	$1.6086\times 10^{1}$	$1.6086\times 10^{1}$	$1.6097\times 10^{1}$	$1.6628\times 10^{1}$	$1.6344\times 10^{1}$	$1.6159\times 10^{1}$	$1.6086\times 10^{1}$	$1.6086\times 10^{1}$	$1.6086\times 10^{1}$
	Rank	1	3	4	9	6	8	7	2	5
F103	Mean	$5.2651\times 10^{-1}$	$5.2879\times 10^{-1}$	$5.2719\times 10^{-1}$	$5.3632\times 10^{-1}$	$5.3170\times 10^{-1}$	$5.3146\times 10^{-1}$	$5.3706\times 10^{-1}$	$5.3345\times 10^{-1}$	$5.2746\times 10^{-1}$
	Std	$1.0774\times 10^{-3}$	$3.1662\times 10^{-3}$	$1.4669\times 10^{-3}$	$4.8085\times 10^{-3}$	$3.9020\times 10^{-3}$	$4.1813\times 10^{-3}$	$0.0000\times 10^{0}$	$3.8998\times 10^{-3}$	$1.7901\times 10^{-3}$
	Best	$5.2577\times 10^{-1}$	$5.2577\times 10^{-1}$	$5.2559\times 10^{-1}$	$5.2735\times 10^{-1}$	$5.2628\times 10^{-1}$	$5.2735\times 10^{-1}$	$5.3706\times 10^{-1}$	$5.2735\times 10^{-1}$	$5.2573\times 10^{-1}$
	Rank	1	4	2	8	6	5	9	7	3
F104	Mean	$2.2306\times 10^{6}$	$1.6755\times 10^{7}$	$3.1862\times 10^{7}$	$2.5304\times 10^{7}$	$3.5806\times 10^{7}$	$4.8788\times 10^{7}$	$8.2489\times 10^{6}$	$3.8492\times 10^{6}$	$5.4721\times 10^{7}$
	Std	$7.1677\times 10^{5}$	$1.5047\times 10^{7}$	$1.3244\times 10^{7}$	$1.4173\times 10^{7}$	$1.7269\times 10^{7}$	$2.4249\times 10^{7}$	$5.9647\times 10^{6}$	$9.3137\times 10^{6}$	$2.1778\times 10^{7}$
	Best	$1.2113\times 10^{6}$	$2.3845\times 10^{6}$	$8.3863\times 10^{6}$	$3.8366\times 10^{6}$	$4.7578\times 10^{6}$	$2.4951\times 10^{6}$	$9.2477\times 10^{5}$	$7.1267\times 10^{5}$	$1.8073\times 10^{7}$
	Rank	1	4	6	5	7	8	3	2	9
F105	Mean	$1.0327\times 10^{0}$	$1.0772\times 10^{0}$	$1.1330\times 10^{0}$	$1.2840\times 10^{0}$	$1.0468\times 10^{0}$	$1.1650\times 10^{0}$	$1.4543\times 10^{0}$	$1.1312\times 10^{0}$	$1.0520\times 10^{0}$
	Std	$4.8709\times 10^{-2}$	$1.0530\times 10^{-1}$	$5.6981\times 10^{-2}$	$2.3795\times 10^{-1}$	$9.7593\times 10^{-2}$	$1.0514\times 10^{-1}$	$1.0133\times 10^{-1}$	$9.1610\times 10^{-2}$	$5.2447\times 10^{-2}$
	Best	$9.9577\times 10^{-1}$	$9.9587\times 10^{-1}$	$1.0206\times 10^{0}$	$9.9577\times 10^{-1}$	$9.9577\times 10^{-1}$	$9.9597\times 10^{-1}$	$1.1676\times 10^{0}$	$9.9578\times 10^{-1}$	$9.9579\times 10^{-1}$
	Rank	1	4	6	8	2	7	9	5	3
F106	Mean	$9.8602\times 10^{-4}$	$1.0425\times 10^{-3}$	$1.0007\times 10^{-3}$	$1.8753\times 10^{-3}$	$1.1724\times 10^{-3}$	$1.3504\times 10^{-3}$	$1.5515\times 10^{-3}$	$1.1551\times 10^{-3}$	$1.1344\times 10^{-3}$
	Std	$7.9683\times 10^{-12}$	$1.1401\times 10^{-4}$	$2.0675\times 10^{-5}$	$3.4971\times 10^{-4}$	$1.1281\times 10^{-4}$	$2.7968\times 10^{-4}$	$4.1712\times 10^{-4}$	$2.1097\times 10^{-4}$	$1.4135\times 10^{-4}$
	Best	$9.8602\times 10^{-4}$	$9.8602\times 10^{-4}$	$9.8720\times 10^{-4}$	$1.0116\times 10^{-3}$	$9.8616\times 10^{-4}$	$9.8629\times 10^{-4}$	$9.8833\times 10^{-4}$	$9.8602\times 10^{-4}$	$9.8731\times 10^{-4}$
	Rank	1	3	2	9	6	7	8	5	4
Total Rank		6	21	24	48	34	43	38	27	29
Final Rank		1	2	3	9	6	8	7	4	5

**Table 11:** Friedman test results for real-world engineering design problems.

Algorithm	Friedman	
	MeanRank	Rank
SO	4.9793	2
ED	4.9862	3
PGA	5.0415	9
TTAO	5.0092	6
HGWO	5.0300	8
EIWOA	5.0184	7
CFDA	4.9931	4
MGO	4.9977	5
IMGO	4.9447	1
*p*-value	$4.1116\times 10^{-4}$	

**Table 12:** Wilcoxon signed-rank test results for real-world engineering design problems.

IMGOvs	Score			
	*p* Value	R+	R−	+/=/−
SO	$2.85\times 10^{-2}$	99.00	359.17	4/1/1
ED	$2.29\times 10^{-3}$	54.33	397.00	5/0/1
PGA	$2.15\times 10^{-5}$	31.50	433.50	6/0/0
TTAO	$6.54\times 10^{-2}$	71.50	388.67	5/1/0
HGWO	$2.15\times 10^{-6}$	10.00	450.17	6/0/0
EIWOA	$2.35\times 10^{-6}$	14.50	450.50	6/0/0
CFDA	$1.63\times 10^{-1}$	84.00	381.00	5/1/0
MGO	$7.34\times 10^{-3}$	80.50	373.50	5/0/1

## Data Availability

The data used in this study are available from the corresponding author upon reasonable request.
